# Abstracts from the 5th International Scientific Conference on Exercise and Quality of Life: Novi Sad, Serbia. 11-13 April 2019

**DOI:** 10.1186/s13102-019-0119-7

**Published:** 2019-05-31

## Invited speakers

### S1 Model of children's comprehensive movement education in a family as a fundament of healthy, physically active, successful and long life

#### Wlodzimierz Starosta^1,2^ (wlodzimierz.starosta@insp.waw.pl)

##### ^1^International Association of Sport Kinetics; ^2^State Research Institute of Sport in Warsaw, Poland

The number of people who practices regular physical exercises in relatively low. **PURPOSE:** Building a new model for children movement education in a family. The proposed model is open and universal, can be modified by the parents in connection to financial possibilities and interest of a child. **METHODS:** Material collected on 2500 subjects of different age and with various methods: test global-movement coordination, the study of development movement abilities in ontogenesis, analysis contents of 29 sports as an element of a model for children movements development. **RESULTS:** The base the model are so willingly practiced by girls and boys of different countries. Suggested model was verified during 6 years on 2000 children. Part of the model was applied in ex-USSR, Sweden, Germany, Italy, Brazil and Uruguay. **CONCLUSIONS:** The suggested model is to be used not only in a family but also in the kindergarten, school, sports classes, and club, or any other institution connected with physical education. Realization of the model allows developing successfully movement coordination in a child during the most suitable age and with a wide range of means. It helps to develop versatile physical and movement abilities in a child that prepares it to active life, improves health, and physical fitness. It is a good base for top-level sport, sport for all or recreation.

### S2 Field-based tests for the assessment of physical fitness in youth practicing sports: a systematic review within the ESA program

#### Antonino Bianco (antonino.bianco@unipa.it)

##### Department of Psychology, Educational Science and Human Movement, University of Palermo, Italy

**PURPOSE:** To systematically review the field-based tests used in the literature to assess Physical Fitness (PF) in children and adolescents practicing sport within the European context. **METHODS:** PubMed and Scopus databases were adopted. **RESULTS:** A total of 123 articles were included in the final review. The adopted batteries were EUROFIT, KTK, National Federations’ batteries, HIRTZ, ALPHA, and BOT2. The others were generic batteries. Muscular strength/power was assessed through a variety of tests in 52 studies (67.5%). Among these, lower body strength was assessed through vertical jumps by 72.3% of them while the upper body strength was assessed through dynamometry in 14.5% of the studies and through medicine ball throw in 20.5% of the papers. A total of 55.3% of the studies assessed speed, through sprint of 5-40 or 60m; 4x10m sprint. 50% of studies assessing coordination used the KTK, and the other half used obstacle-run, walk-backward, plate-tapping, eye-hand-foot coordination. **CONCLUSION:** The present study provides a framework of the field-based tests used to assess PF in children and adolescents practicing sport across European countries. High heterogeneity was evidenced among the used tests for health- and skill-related fitness assessment. Ultimately, the review aims to suggest a new fitness test battery that will fit the needs of the consortium.

### S3 Exercise and quality of life in the elderly

#### James S. Skinner (jimskinnrphd@gmail.com)

##### Indiana University, Bloomington, USA

**PURPOSE**: This presentation summarizes the literature on exercise and quality of life (QoL) in the elderly. Aging is associated with a decline in volume and intensity of physical activity, resulting in a decrease in muscle size, strength, power, and aerobic performance. These factors contribute to a reduction in mobility, self-confidence, independence, and QoL. Regular participation in exercise can delay or prevent many of the declines associated with aging and has a positive effect on many factors associated with a reduced QoL. Active people also perceive that their QoL is higher than do sedentary people. **CONCLUSIONS**: Regular exercise improves psychological health and wellbeing. Examples of improvements include increased self-concept and self-esteem, reduced risk for clinical depression and anxiety, dementia and cognitive decline, and fear of falling. While exercise has been consistently shown to be beneficial, the effects are often moderate and variable. As a result, the optimal programs to improve QoL are not known. There are suggestions that group-based programs are better than home-based programs and that this is related to the social aspects of exercising in a group. Several studies suggest that moderate-intensity exercise is better than low- or high-intensity exercise. Nevertheless, many different types of exercise have been shown to be beneficial, including aerobic training, resistance or strength training, walking, hopping, swimming, aquatic exercise, as well as exercises to improve flexibility and balance.

### S4 Fitter kids for a healthier adult society

#### Francisco B. Ortega^1,2,3^ (ortegaf@ugr.es)

##### ^1^Co-Director of the PROFITH “PROmoting FITness and Health through physical activity” research group; ^2^Department of Physical Education and Sports, Faculty of Sport Sciences, University of Granada, Spain; ^3^Department of Biosciences and Nutrition at NOVUM, Karolinska Institutet, Huddinge, Sweden

From the landmark studies of Prof. Paffenbarger and Blair in the 70's and 80's to date, an enormous amount of evidence has accumulated supporting a strong link between the cardiorespiratory fitness (CRF), and more recently also muscular strength (MST), in adulthood and overall and specific morbidity and mortality. Several longitudinal studies also showed that physical fitness (both CRF and MST) in childhood and adolescence are associated with lower risk factors for cardiovascular diseases and other health outcomes. However, whether physical fitness assessment at these young ages directly predict future mortality and morbidity was unknown, since very large cohorts and with a very long follow-up are needed to test these hypotheses and it has been a lack of such studies. During my presentation in this scientific event, I will cover this topic, presenting the state of the art in relation to fitness in youth and future health. I will discuss not only the importance of fitness for physical health but also for better brain health, including both mental health and cognition/brain enhancement.

### S5 How can health literacy influence health status?

#### Jozsef Betlehem, Henrietta Banfai-Csonka, Attila Pandur, Bence Schiszler, Balint Banfai, Pongrac Acs, Balazs Radnai

##### Faculty of Health Sciences, University of Pecs, Hungary

###### **Correspondence:** Jozsef Betlehem (betlehem@etk.pte.hu)

**PURPOSE:** Health literacy-based interventions gain greater attention in each developed society. Our purpose was to conduct a systematic review and determine if health literacy might benefit health outcomes. **METHODS:** Data was collected from English-language reviews using PubMed between 2008-2018. The reviewers shared the work: abstracting article information; checking information for accuracy; deciding on overall appropriateness and synthesis. Keywords for search: health literacy, health status, review. **RESULTS:** The frequency of review articles in a correlation of health literacy and health status increased in the past ten years (PubMedsum:53;2009:4;2018:12). The review articles reported a clear relationship between health literacy and health status independently from the conceptual framework. A narrow and wide concept of health literacy can be observed. USA studies focus on functional health literacy influencing health status mainly while European studies include control over health and personal, social, environmental factors, too. Differences in health literacy level were consistently associated with increased hospitalizations, greater emergency care use, poorer ability to take medications appropriately, poorer ability to interpret labels and health messages, poorer overall health status, and even higher mortality. Using intervention and looking for their effect is one way of changing the health literacy level of society which is used widely recently. **CONCLUSION:** Low health literacy is associated with poorer health outcomes in all reviews and therefore it is a good predictor of health status.

### S6 Exercise and quality of life in healthy adolescents, women with eating disorders and female elite athletes

#### Jorunn Sundgot-Borgen (jorunn.sundgot-borgen@nih.no)

##### Department of Sports Medicine, The Norwegian School of Sport Sciences, Oslo, Norway

The link between exercise and quality of life is understudied in some groups. Among those are a) adolescents with body dissatisfaction, b) women with eating disorders and c) female elite athletes. A high prevalence of adolescents is struggling with body dissatisfaction and body image predicts health-related quality of life (HRQoL) more strongly than other variables. However, the long-term effect of school-based intervention studies aiming to improve body image and HRQoL is not known. Women with eating disorders report impaired quality of life (QoL) and well-being. However, the effect of different treatment interventions (including physical exercise) on QoL is inadequately addressed. Finally, evidence regarding QoL in female elite athletes is lacking. This presentation will focus on recent studies and findings regarding 1. The long-term effects of a school-based intervention study aiming to improve body image and HRQoL, 2. The effect of treatment (physical exercise and nutrition guidance) on eating disorder symptomatology and QoL in women with eating disorder and 3. QoL in elite female athletes who are mothers.

### S7 Impact of 5-2-1-0: a community-based childhood obesity prevention initiative

#### Dejan Magoc (dmagoc@stetson.edu)

##### Department of Health Sciences, Stetson University, DeLand, Florida, USA

‘5-2-1-0’ is a nationally recognized initiative with a goal to raise awareness about the importance and benefits of healthy eating and active living among children. **PURPOSE**: To improve healthy daily behaviors in four domains (5 servings or more of fruits and vegetables (FV); 2 hours or less of screen time (e.g., tablets, video games); 1 hour or more of physical activity (PA); 0 sugary drinks/more water) that have been demonstrated to impact overweight and obesity among children. **METHODS**: One-hundred-eighteen children (64 girls and 54 boys; age 8.6 ± 1.6 years; BMI 20.7 ± 9.2 kg/m^2^) enrolled in the summer camp and after-school programs in DeLand, Florida, participated in this quasi-experimental study design. Participants completed a questionnaire that assessed and tracked changes in their health behaviors in four, aforementioned, domains. **RESULTS**: Following a 12-week prevention initiative, participants significantly a) increased daily minutes of PA, t(117) = 2.2, p < 0.05; b) increased daily intake of FV, t(117) = 2.3, p < 0.05; and c) decreased daily usage of electronic devices, t(117) = 2.7, p < 0.05. **CONCLUSION**: A community-based awareness approach to improve healthy eating and active living was associated with improved child behavior changes. Longitudinal studies could provide a better image of how sustainable these changes become over time.

**ACKNOWLEDGMENTS**

Supported by the Community-Based Research Office, Stetson University.

### S8 Physical (in)activity in early school-age: what are we doing wrong?

#### Sanja Music Milanovic^1,2^ (sanja.music@hzjz.hr)

##### ^1^Croatian Institute of Public Health, Health Promotion Division, Zagreb, Croatia; ^2^School of Medicine, School of Public Health “Andrija Stampar”, University of Zagreb, Croatia

Physical inactivity is one of the key risk factors for the leading chronic non-communicable diseases. Physically inactive children are at higher risk of becoming inactive adults. In Croatia, at the age of 8, 89.8% of children reach the recommended 60 minutes of physical activity per day, by engaging in free outdoor play. However, at the age of 15, only 19% of adolescents reach these recommendations. What happens during this period? Why are our children becoming more and more inactive as they age? While this phenomenon has been observed worldwide and is often associated with various physical, social and psychological changes that occur with maturation, we have to ask ourselves what can be done to diminish this occurrence. The Croatian National Health Promotion Program „Healthy Living“ aims to promote healthy lifestyles by offering support for engaging in various forms of physical activity, in different settings throughout the lifespan.

### S9 The post-millennials' lifestyle and what physical education can do about it?

#### Visnja Djordjic (djordjicvisnja@gmail.com)

##### Faculty of Sport and Physical Education, University of Novi Sad, Serbia

Post-millennials, also known as Generation Z, are the first generation born in an entirely digital world. This demographic cohort, influenced strongly by technology and social media, includes people born 1997-present. Post-millennials share some distinctive lifestyle features in comparison to other generations: they are more sedentary, less active, more digitally dependent, and less exposed to the natural environment. Excessive use of electronic media, in particular, may affect overall health and wellbeing of children and youth. Possible health risks include obesity, sleep disorders, mental health issues, lack of physical fitness and motor competence, postural disorders, etc. At the same time, post-millennials are frequently described as multi-taskers, open-minded, diverse, global and health aware, which also has to be considered when designing quality physical education programs. If physical education is to remain an effective social strategy for increasing physical activity, it has to be shaped by the specific needs and interests of today’s youth. In that regard, a shift toward more holistic and culturally responsive physical education, with a focus on health, inclusion, and fitness might be necessary. Enhancing student’s perceived and actual motor competence through a variety of positive, personally relevant learning experiences, is vital for lifelong physical activity participation. Moreover, a whole-school approach to physical activity promotion, which provides a lot of opportunities for students to be active before, during, and after school hours, has been recommended.

### S10 Physical activity and motor skills in preschool children

#### Sanja Salaj (sanja.salaj@kif.hr)

##### Faculty of Kinesiology University of Zagreb, Croatia

Competence in the field of motor skills contributes to children's physical, cognitive and social development. Motor proficiency is positively associated with health benefits and negatively associated with obesity. Children with a higher level of motor skills tend to be more active than those with lower level motor skills. Different countries have set their recommendation on daily physical activity of preschool children from 60 to 180 minutes of moderate‐to‐vigorous physical activity, in some cases with more specific requirements of different structured and free play. Although often assumed that young children are very active throughout the day, recent studies indicate that young children spend a majority of the day in sedentary activities and spend <5% of the day in moderate and vigorous physical activity (MVPA). Reasons for low physical activity levels are not well understood, but one of the suggestions is a low level of motor proficiency. Few studies have examined the relationship between physical activity and motor skills in children and found a significant but small relationship of motor skills to physical activity in general, rather higher when compared to MVPA. There are other factors that influence physical activity in youth, but recent evidence suggests that motor skill learning may be important in promoting a physically active lifestyle in preschool children.

### S11 HEPA Europe – Before – Now – and ???

#### Finn Berggren^1,2,3^

##### ^1^Board member and co-founder, HEPA Europe; ^2^International Adviser/Past President and CEO; ^3^Gerlev P.E. & Sports Academy/Gerlev Center for Play and Move, Denmark

HEPA Europe is a European Network for the promotion of health-enhancing physical activity (PA) and founded in May 2005 in Gerlev (Denmark). The vision of the Network is to achieve better health through PA among all people in Europe. The Network has been growing every year and today there are 171 member-institutions and 3 individual members from 38 countries. Its goal is to strengthen and support efforts and actions that increase participation and improve the conditions favorable to a healthy lifestyle. The objectives are to: promote a better understanding of health-enhancing PA and give a stronger voice to PA promotion in health policy and in other relevant sectors in Europe, including support for workforce development; develop, support, and disseminate effective strategies and multi-sectoral approaches in the promotion of health-enhancing PA; foster the preservation and creation of social and physical environments as well as values and lifestyles supportive of health-enhancing PA; together with other relevant institutions and organizations, improve coordination in PA promotion across sectors and administrative structures. HEPA Europe is not just a network meeting annually but Working Groups are established based on a proposal by the Steering Committee to facilitate the implementation of specific projects and activities, as agreed in the program of work of the Network. Since 2005 the Network has published several scientific publications.

## Oral presentations

### O1 Hydrogen-rich water positively affects HOMA2 variables in physically inactive population

#### Darinka Korovljev, Dejan Javorac, Valdemar Stajer, Sergej M. Ostojic

##### Faculty of Sport and Physical Education, University of Novi Sad, Serbia

###### **Correspondence:** Sergej M. Ostojic (sergej.ostojic@chess.edu.rs)

Supplemental molecular hydrogen improves biomarkers of cardiometabolic health in physically active men and women yet no human studies evaluated its effects on steady state beta cell function and insulin sensitivity in non-active humans. **PURPOSE:** To evaluate the effects of 28-day intake of hydrogen-rich water (HRW) on Homeostasis Model Assessment **(**HOMA2) outcomes and body composition in a cohort of physically inactive men and women. **METHODS**: Twelve overweight inactive volunteers (7 women and 5 men; age 56.2 ± 10.0 years; BMI 37.7 ± 5.3 kg/m^2^) participated in this randomized, double-blind, placebo-controlled, crossover trial. Participants were allocated to receive either HRW (1 L/day) or placebo (tap water) for 28 days. HRW was produced by dissolving an effervescent magnesium tablet (HRW Natural Health Products Inc., New Westminster, Canada) into a cup of water (500 mL). **RESULTS:** Drinking HRW was superior to placebo to increase insulin sensitivity (for up to 11.1%; p < 0.05), and tended to reduce body fat at 28-day follow-up more as compared to placebo (- 4.2% vs. 0.6%; p = 0.09). **CONCLUSION:** The results of this trial nominate HRW as a possible nutritional strategy to improve insulin sensitivity in overweight inactive population. Registered at ClinicalTrials.gov (NCT03625362).

**ACKNOWLEDGMENTS**

Supported by the Serbian Ministry of Education, Science and Technological Development (175037), the Provincial Secretariat for Higher Education and Scientific Research (114-451-710), the Faculty of Sport and Physical Education, and HRW Natural Health Products Inc.

### O2 Physical fitness and body mass index in Algerian university students

#### Mohammed Zerf^1^, Hadjar Mohamed Kherfane^1^, Regig Madani^1^, SBA Bouabdellah^2^, Boras Fatima Zohra^1^, Gourari Benali^1^

##### ^1^Faculty of Sport and Physical Education, University of Mostaganem, Algeria; ^2^Faculty of Sport and Physical Education, University Hassiba Ben Bouali of Chlef, Algeria

###### **Correspondence:** Mohammed Zerf (biomeca.zerf@outlook.com)

Physical fitness is acknowledged to have profound effects on public health worldwide, with regular collecting data likely being crucial for addressing the problem and implementing different strategies to fight physical inactivity. **PURPOSE**: To determine basic physical fitness components and body mass index in university students in Algeria. **METHODS**: Two hundred healthy university students (20 to 24 years old), with no regular sports activities were randomly recruited for this study, during the academic year 2016-2017. They were tested in several basic physical fitness tests (Pushups. Sit-ups, Chin-Ups, and 1.5 Mile Run), and body mass index. **RESULTS**: Average values of fitness tests result showed a decreased level of physical fitness and a high incidence of overweight and obesity in University students. **CONCLUSION**: Low level of physical fitness and a high incidence of obesity in Algerian students is likely at least partially related to physical inactivity. Hence, there is evidence enough to justify the further development of public health policies to promote physical activity in Algerian student population.

### O3 The influence of exercise on the functional status and quality of life of patients that were surgically treated for breast cancer

#### Sanja D. Tomic^1^, Goran Malenkovic^1,2^, Slobodan V. Tomic^1^

##### ^1^University of Novi Sad, Faculty of Medicine, Department of Nursing, Novi Sad, Serbia; ^2^Institute for Oncology of Vojvodina in Sremska Kamenica, Serbia

###### **Correspondence:** Sanja D. Tomic (sanja.tomic@mf.uns.ac.rs)

Breast cancer surgery is connected with the risk of developing functional limitations that can negatively influence the quality of life. **PURPOSE**: Estimate the influence of early postoperative exercises on the quality of life 3 months after surgery. **METHODS**: The prospective study involved 149 patients, divided into groups according to surgical intervention. Group A consisted of 67 patients with an average age of 57.13 ± 13.72 group Q 82 patients with an average age of 58.34 ± 11.59. Assessment of the quality of life was done by the SF-36 questionnaire and functional testing before and 3 months after surgery. All data was input in the StatSoft software (Statistica 10.0 StatSoft, Inc., Dell, USA) and displayed as average values with standard deviations. Analysis has estimated the impact of the intervention comparing findings before and 3 months after surgery using t-test and Leven's test, a correlation was displayed by using Spearman's coefficient. **RESULTS**: After exercise mental, physical and general health in both groups of participants (MH 63.74% versus 49.70%, PH 66.99% versus 53.46%, GH 65.37% versus 51.58%) improved. **CONCLUSION**: Early postoperative exercises contribute to functional recovery and facilitate the increase in life quality.

### O4 Positive effects on muscular fitness after 10 weeks of ball game training in primary school children

#### Dejan Madic, Nebojsa Trajkovic, Danilo Radanovic, Drazenka Macak

##### Faculty of Sport and Physical Education, University of Novi Sad, Serbia

###### **Correspondence:** Dejan Madic (dekimadic@gmail.com)

A growing body of research has highlighted the health benefits of football training in children. It is unclear, however, whether different ball training at school can have similar favorable effects. **PURPOSE**: To determine the effects of a 10-week ball game training program on muscular fitness in children aged 12-14 years. **METHODS**: A total of 64 school children were randomized to ball game group (푛=32; age 13.26 ± 0.29; height 163.49 ± 9.97 cm; weight 50.29 ± 10.31 kg), or a control group (푛=32; age 13.45 ± 0.47; height 168.27 ± 9.30 cm; weight 55.08 ± 9.55 kg). The ball game group had twice per week (≈40min) small-sided games training (football, basketball, handball, volleyball) for 10 weeks. The control group attended their regular PE class twice per week. **RESULTS**: The groups did not differ significantly at baseline in any fitness performance measures. Significant Group by Time interactions were noted for vertical jump, standing long jump, medicine ball throw, bent arm hang and sit and reach, F = 12.121, 62.567, 19.917, 39.580, 5.276, respectively, p < 0.05, with ball game group making significantly greater improvement than the control group. **CONCLUSION**: A 10-week school-based ball game intervention was effective to improve muscular fitness in children aged 12-14.

### O5 Free-time activity, sustainability and healthy lifestyle along the river Tisza. marking cultural and nature conservation trails

#### Judit Raffai, Ester Gabric, Geza Cekus

##### Hungarian Language Teacher Training Faculty, University of Novi Sad, Serbia

###### **Correspondence:** Judit Raffai (raffaij6@gmail.com)

The present study elaborates upon the outcomes of the WATERTOUR (HUSRB/1602/31/0204) project from the aspects of the free-time activity, sustainability, and healthy lifestyle. **PURPOSE**: Local opportunities and actions will be discussed regarding the encouragement of a healthy lifestyle, which could offer recreational activities along the promotion of cultural and natural values. **PROJECT PRESENTATION**: According to the Statistical Office, in 2015 the female members of the society (above the age of 15) disposed of 6 hours of free time daily, while the male members 7. However, men tended to spend 30 minutes doing sports activities/spending time in nature, while women only 16. The present project aims to encourage tourism and sports activities along the river Tisza as well as to provide cultural and nature conservation trails. Our objective is to establish land trails along the river Tisza (Kanjiza, Novi Knezevac, Senta and Coka) that merge cultural, architectural, ethnographical and natural values which aim to attract various generations, families, offer spending quality time in nature. **CONCLUSION**: With the support of local municipalities, the established cultural and nature conservation trails along the river Tisza will increase the recreational opportunities while ensuring familiarization with local values.

### O6 Making a move: increasing physical activity using smartphones and android applications

#### Zolt Namestovski, Dijana Karuovic^2^, Cintija Kovac^1^, Lenke Major^1^, Molnar Gyorgy^3^

##### ^1^Hungarian Language Teacher Training Faculty, University of Novi Sad, Serbia; ^2^Technical faculty "Mihajlo Pupin", University of Novi Sad, Serbia; ^3^Budapest University of Technology and Economics, Hungary

###### **Correspondence:** Zolt Namestovski (zsolt.namesztovszki@magister.uns.ac.rs)

Mobile phones are becoming increasingly popular worldwide. According to the Statistical Office of the Republic of Serbia, in 2018, 93.0% of all households own a mobile phone and have Internet connection via mobile (smart) phones, while tablets (3G network) proved to be the most popular form of connection to the Internet, with 67.5% of households opting for this type of connection in 2018. **PURPOSE**: To report and review the possibilities and opportunities of smartphones and Android applications for increasing physical activities for students in Serbia and Hungary. **CASE PRESENTATION**: Smartphones incorporate many different sensors, which can be used to measure and boost regular physical activity (GPS sensors, vision sensors, audio sensors, light sensors, temperature sensors, direction sensors, and acceleration sensors). Moreover, there are specific additional services, such as social and online experience (competitions within the online community and competing with one's own results), notifications and gamification. **CONCLUSION**: Based on the literature review, categorization and reviewing mobile applications and a pilot survey among the students, it can be concluded that there is a wide range of opportunities to increase physical activities using smartphones and their Android applications.

### O7 The development of stress relief and problem resolution strategies among pre-service teachers via biological-cultural programs along the River Tisza

#### Valeria Pinter Krekity, Lenke Major, Zolt Namestovski, Rita Horak, Agnes Baganj

##### Hungarian Language Teacher Training Faculty, University of Novi Sad, Serbia

###### **Correspondence:** Zolt Namestovski (zsolt.namesztovszki@magister.uns.ac.rs)

The profession of teaching proves to be one of the most stressful occupations. The continuous effects of stress among pre-service teachers have a negative influence on their physical and mental health. **PURPOSE**: The present research aims to introduce a preventive program that offers participation in free-time, sports and cultural activities performed in nature. The program which aims to impact the participants’ complex, physical and mental health is based on pedagogical, methodological and health-developmental principles. **METHODS**: The Rahe-type Brief Stress and Coping Questionnaire were conducted among the students of the Hungarian Language Teacher Training Faculty, University of Novi Sad. **RESULTS**: According to the results, symptoms of severe depression and anxiety were detected. However, assistance in coping with stress in various life aspects is highly required. **CONCLUSION**: The psychological well-being of pre-service teachers and pre-school teachers are fundamentally influenced by stress and its coping mechanisms. Familiarization with the biological-cultural values along the river Tisza offers preventive, complex programs to establish direct contact with the natural and social environment as well as to ensure its sustainability. Meanwhile, it also enables pre-service teachers to acquire various stress-relief techniques and develop their stress coping mechanisms.

### O8 Variations in adiposity, body fat percentage, and muscular strength, according to physical activity level in young adults

#### Omer Barıs Kaya^1^, Mustafa Sogut^1^, Kubra Altunsoy^1^, Cain CT. Clark^2^

##### ^1^Faculty of Sport Sciences, Kırıkkale University, Turkey; ^2^Department of Sport, University Centre Hartpury (University of the West of England), Gloucestershire, UK

###### **Correspondence:** Mustafa Sogut

**PURPOSE**: The aims of this study were to examine the discrepancies between moderately physically active (MPA) and highly physically active (HPA) male (n=96, age=22.5±1.7 years) and female (n=85, age=21.3±1.6 years) young adults regarding various anthropometric adiposity indices (AIs), body fat percentage (BF%), and muscular strength, and to determine the associations between physical activity level (PAL) and the aforementioned variables. **METHODS**: Participants’ height, body mass, BF%, waist and hip circumferences, and maximal isometric grip strength was measured. They were dichotomized according to their PAL, estimated by the short version of the International Physical Activity Questionnaire (IPAQ), as MPA (≥600-3000 MET-min/week) and HPA (≥3000 MET-min/week). **RESULTS**: Participants in the HPA groups had significantly lower BF% (ES 0.67 ♂; 0.72 ♀), body mass (ES 0.46 ♂; 0.58 ♀), waist circumference (ES 0.55 ♂; 0.48 ♀), hip circumference (ES 0.46 ♂; 0.49 ♀), and BMI (ES 0.46 ♂; 0.59 ♀) than the participants in the MPA groups in both genders. Grip strength performances were comparable between groups. The PAL, regardless of gender, was found to be significantly and negatively correlated with all AIs and BF%. **CONCLUSION**: These findings suggest that high habitual physical activity level mediates body size and composition among young adults.

### O9 Agreement in estimates of body fat percentage between BIA and BMI-based body fat equations in female young adults

#### Kubra Altunsoy^1^, Mustafa Sogut^1^, Omer Barıs Kaya^1^, Cain CT. Clark^2^

##### ^1^Faculty of Sport Sciences, Kırıkkale University, Turkey; ^2^Department of Sport, University Centre Hartpury (University of the West of England), Gloucestershire, UK

###### **Correspondence:** Mustafa Sogut

**PURPOSE**: The purpose of this study was to compare the body fat percentage (BF%) values estimated with various body mass index (BMI)-based BF% equations and bioelectrical impedance (BIA). **METHODS**: One hundred and eighty-three female young adults (age=20.5±1.8 years, BMI=21.5±3.1kg/m2) participated in the study. Height and body mass were measured to calculate BMI. BF% was determined by BIA and predicted using BMI-based equations (BMIDE; BMIJA; BMIWO; BMIGA). **RESULTS**: Dependent t-test results revealed that there was no significant difference (P>0.05) in BF% between BIA and BMIJA. However, significant differences (P<0.01) were found between BIA and all other equations in BF%. The magnitude of difference, when compared to BIA, was trivial for BMIJA (ES 0.10), and small for BMIDE, BMIWO, and BMIGA (ES 0.24, 0.47 and 0.24, respectively). The standard error of estimate ranged from 3.85 (BMIJA) to 3.91% (BMIGA). Bland-Altman analysis indicated that the 95% limits of agreement were narrowest for BMIJA (±7.62%) and widest for BMIDE (±8.47%). **CONCLUSION**: These results highlight the practical usefulness of BMIJA equation in predicting BF% among female young adults when BIA, one of the most ubiquitous field techniques, is not available.

### O10 The pedagogical potential of a user-friendly specialized dictionary in function of adopting a healthy lifestyle

#### Mira Milic^1^, Filip Sadri^1^, Tatjana Glusac^2^

##### ^1^Faculty of Sport and Physical Education, University of Novi Sad, Serbia; ^2^Faculty of Law and Business Studies, Union University, Belgrade, Serbia

###### **Correspondence:** Mira Milic (mmilic@uns.ac.rs)

Even though specialized dictionaries provide abundant information, research findings indicate that their role in the teaching process has been neglected. Within the context of the current global domination of English and an increased need for linguistic standardization, special emphasis is placed on the use of specialized dictionaries in teaching vocabulary. **PURPOSE**: To analyze the pedagogical potential of a user-friendly specialized dictionary in function of adopting a healthy lifestyle. **METHOD**: A questionnaire-based research into dictionary use in ESP acquisition is conducted with 705 students of non-linguistic faculties of the University of Novi Sad. **RESULTS**: Quantitative research indicates students' insufficient knowledge not only of lexicographic conventions but also the criteria for dictionary quality assessment, whereas the qualitative analysis reveals a preference for online dictionaries and other user-friendly applications. **CONCLUSION**: Building on the hypothesis that well-conceived dictionaries can contribute to teaching an active lifestyle in non-English speaking regions, this research suggests the importance of quality terminological products and systematic training in dictionary use.

**ACKNOWLEDGMENTS**

This paper is a part of the research on Project No. 142-451-3684/2017-01/01, entitled Using Dictionaries in Teaching English for Specific Purposes in Tertiary Education, financially supported by the Secretariat for Higher Education and Scientific Research of the Autonomous Province of Vojvodina, Serbia.

### O11 Exercise-based treatment after an ACL injury and a meniscal surgical repair: A case report

#### Mario J. Roska^1^, Natasa Pencic^2^, Alessandro Ruspantini^3^

##### ^1^Corrective Exercise Center “Howfit”, Novi Sad, Serbia; ^2^Faculty of Sport and Physical Education, University of Novi Sad, Serbia; ^3^Aspire Academy, Doha, Qatar

###### **Correspondence:** Mario J. Roska (marioroska@gmail.com)

**PURPOSE:** In this case report we will demonstrate the effects of an exercise-based treatment after an ACL injury and a meniscal surgical repair in an elite UEFA Women's Champions League soccer goalkeeper with the main goal to successfully return to play. **CASE PRESENTATION:** The assessment consisted of two questionnaires for psychological readiness to return to play, physical readiness was assessed via muscular strength, agility, speed, cardio-respiratory fitness, coordination, and lower limb asymmetry via single-leg hop tests. The five-month exercise-based program consisted of aquatic exercises, gym therapy sessions and on-field (sport-specific) training. The final results showed an increase in psychological readiness to return to play by 60%, physical functions improved by 72%, and lower limb asymmetry was not higher than 10%. **CONCLUSION:** Exercise-based therapy has shown to be an effective way to return a female soccer goalkeeper to play and it should be considered as a treatment option for an athlete who suffered from an ACL injury or a surgical meniscus repair.

### O12 Link with motivation and psychological states of elite athletes

#### Georgiy Korobeynikov^1^, Mario Baic^2^, Lesia Korobeynikova^1^, Bahman Mirzaei^3^

##### ^1^Department of Biomechanics and Sport Metrology, National University of Physical Education and Sport, Ukraine; ^2^Faculty of Kinesiology, University of Zagreb, Croatia; ^3^Department of Exercise Physiology, Faculty of Sport Sciences, University of Guilan, Iran

###### **Correspondence:** Georgiy Korobeynikov (george.65.w@gmail.com)

Among different factors which influence to the effectiveness of sports activities the most characteristic is a psychical function of athletes. But, the formation of individual strategies of modern sports activity is corresponding to the motivation process. **PURPOSE**: The study of links between motivation and psychological states in elite athletes. **METHODS**: The 26 athlete's members of the National Team of Ukraine of Greco-Roman wrestling were studied. For the study of motivation to achieve was used the questionnaire (by Mehrabian A). All of the athletes were separated into three groups by level of corresponding motivation. The psychical states studied by an estimate of some methods. **RESULTS**: In athletes with a high level of motivation to achieve were observed the increasing meanings of stability of sensory-movement reaction. At the same time, the more improvement meanings of throughput of the visual analyzer are showed in athletes with an average level of motivation to achieve. **CONCLUSION**: Were revealed the states of relative comfort, desire to act, advance and find support in wrestlers of high-level motivation to achieve. But the low level of motivation to achieve related with a decline of capability and appearance of discomfort.

### O13 Review of qualitative studies analyzing bullying and harassment in sport: methods and research areas

#### Jolita Vveinhardt, Vilija B. Fominiene, Laima Jeseviciute-Ufartiene

##### Faculty of Sports Education, Lithuanian Sports University, Kaunas, Lithuania

###### **Correspondence:** Vilija B. Fominiene (vilija.fominiene@lsu.lt)

Each research area has its own specificity, and sport is unique due to the expression of aggression among sports participants. Despite a rather broad analysis of this area, there is still a lack of knowledge in discussing the phenomenon of bullying and harassment as an expression of aggression in sport, and this may be due to methods used in research. **PURPOSE:** Having systematized the qualitative studies on bullying and harassment in a sport as well as their selected qualitative research methodologies, to discuss the research areas of bullying and harassment. **METHODS:** The PRISMA method was used to select the target articles. Content analysis method was chosen for analyzing the results of the selected articles. **RESULTS:** For qualitative analysis, 23 scientific articles that met the eligibility criterion were selected. The qualitative content analysis of these articles revealed a variety of research elements in them, thus resulting in various research outcomes. **CONCLUSION:** Research results were divided into research areas: bullying and harassment as an object of cognition, the environment of bullying and harassment; bullying and harassment related to gender or age, bullying and harassment as a ritual for new team members; bullying and harassment prevention.

A**CKNOWLEDGMENTS**

Funded by the European Social Fund according to the activity ‘Improvement of researchers’ qualification by implementing world-class R&D projects of Measure No. 09.3.3-LMT-K-712.

### O14 Peer violence in grass-roots sports clubs – coaches’ perspective

#### Borislav Obradovic, Ivana Milovanovic, Roberto Roklicer, Nebojsa Maksimovic

##### Faculty of Sport and Physical Education, University of Novi Sad, Serbia

###### **Correspondence:** Borislav Obradovic (boriscons@yahoo.com)

The research is a part of project Sport against violence and exclusion funded by the Erasmus + Sport 2017 Program, which seeks to prevent peer violence and social exclusion through physical activity. **PURPOSE**: The paper presents results of focus-group discussion conducted with coaches in relation to recognition and prevention of peer violence among children in grass-roots sports clubs. **METHODS**: Focus-group discussion with coaches was conducted at the Faculty of Sport and Physical Education in Novi Sad. **RESULTS**: Participants emphasized that cases of social exclusion and violence among children are more an exception rather than an occurrence. However, they described cases of violence in details, pointing out that children and youth are surrounded by violence in society (at school, on the streets, in media, social networks). They also pointed out the misunderstanding in communication with parents, who interfere "too much" to coaches' work. They also emphasized the fact that parents of violent children do not admit that their children have committed violence, which is a major issue in the process of mediation between violent and "victim" child. **CONCLUSION**: Coaches believe that Faculties of Sports should somehow find a way and organize education of coaches, parents, and children in order to recognize and prevent violence. This kind of education would be a useful starting point in solving this challenging social issue.

### O15 “Governing” social exclusion and violence in the field of youth sport

#### Ivana Milovanovic, Zoran Milosevic, Dejan Madic, Darinka Korovljev, Patrik Drid

##### Faculty of Sport and Physical Education, University of Novi Sad, Serbia

###### **Correspondence:** Ivana Milovanovic (i.a.milovanovic@gmail.com)

The first part of the paper represents a summary of National Policies and Strategies which address the issue of Social Exclusion and peer violence in Serbia. The second part is an overview of Regulation of training, education, and employment of Sports Coaches, who work with children and youth in grass-roots sports clubs in Vojvodina. **PURPOSE**: To examine what policies and strategies are in place in Serbia and implementation of those documents by a government and civil sector organizations. By mapping coaches' educational background, the authors emphasized the need for higher education of coaches, with a special need to educate them in the field of prevention of social exclusion and violence among children. **METHODS**: The data were obtained mostly via online research. Authors conducted additional field research in a form of separate questionnaires delivered to specific national and local government bodies and sports clubs and federations, for the purpose of mapping the coaches on the ground. **RESULTS**: The strategies and policies which address the issue of social exclusion and violence among children and youth are in place. However, their full implementation hasn't seen as a priority on the ground. **CONCLUSION**: Social exclusion and peer violence should be addressed more intensive in terms of academic curricula for coaches and professors of PE and on the ground through prevention, recognition, and regulation of this negative social phenomenon.

### O16 Benchmarks to consider in terms of fitness profile of young volleyball players

#### Suncica Pocek, Dusko Cvijovic, Tatjana Trivic, Zoran Milosevic

##### Faculty of Sport and Physical Education, University of Novi Sad, Serbia

###### **Correspondence:** Suncica Pocek (suncicapocekfsfv@gmail.com)

**PURPOSE**: To examine the fitness profile of young female club volleyball players in terms of age categories and player specialization. **METHODS**: Thirty-five volleyball players were assessed for height, mass, spike reach, block reach, and body composition. Vertical jump, standing broad jump, spike and block jump was chosen as a measure of leg power. The running speed of players was evaluated with a 20 m sprint effort. The change of direction speed was evaluated with a T-test, Sprint 93639 m forward – backward and Sprint 93639 m with 1800 turns. Two-way ANOVA with LSD post hoc test was applied in order to examine statistically significant differences between team A (18.23 ± 1.71 years) and team B (14.38 ± 0.56 years), between positions and their interaction. **RESULTS**: Team A players demonstrated better performance in all conducted tests of motor abilities in comparison to young female players of Team B. According to the position in most of the motor tests applied there were no differences between players. **CONCLUSION**: Establishing comparative fitness measures by competition level and player position should help to create benchmarks by which incoming and current athletes can assess performance.

### O17 A low dose of caffeinated coffee doesn’t increase fat oxidation but decreases RPE during aerobic exercise

#### Karayigit Raci, Sahin Nese, Ersoz Gulfem

##### Faculty of Sport Sciences, Ankara University, Turkey

###### **Correspondence:** Karayigit Raci (racikarayigit@hotmail.com)

Caffeine has been shown to stimulate fat metabolism during exercise, but there is a scarce evidence investigating the effects of low dose caffeine. **PURPOSE**: To evaluate the effects of two different (1.5 – 3 mg/kg) low doses of caffeinated coffee on fat oxidation during low intensity exercise **METHODS**: Fourteen female volunteers (age 21.2 ± 2.4 years; BMI 28.7 ± 3.4 kg/m2; VO2max 42.2 ± 3.7 ml/min/kg) participated in this randomized, double-blind, placebo-controlled, crossover trial. Following to VO2max test, participants were submitted to either decaffeinated coffee (PLA) or very low dose caffeinated coffee (VLCOF) equal to 1.5 mg/kg or low dose caffeinated coffee (LCOF) equal to 3 mg/kg before 45 min to %50 Wattmax exercise (30 min) during which expired air was analyzed. Fat oxidation, RPE, heart rate, glucose, lactate, and RPE were measured at different time point throughout the test protocol. **RESULTS**: Fat oxidation, RPE, heart rate, glucose, lactate didn’t differ between trials (p>0.05) but LCOF significantly decreased RPE values compared to PLA and VLCOF trials (p<0.05). **CONCLUSION**: Results of this study showed that a low dose of caffeinated coffee may decrease RPE during low-intensity exercise. To increase fat oxidation during exercise may require to intake higher dose of caffeine. Future studies should investigate the effects of a moderate dose of caffeinated coffee on substrate oxidation during low and high-intensity aerobic exercise.

### O18 Effects of the group exercise program on the morphological status of female university students

#### Nebojsa Cokorilo¹, Milena Mikalacki¹, Milan Cvetkovic¹, Goran Dimitric^1^, Sasa Pantelic²

##### ^1^Faculty of Sport and Physical Education, University of Novi Sad, Serbia; ²Faculty of Sport and Physical Education, University of Nis, Serbia

###### **Correspondence:** Nebojsa Cokorilo (cokorilon@gmail.com)

**PURPOSE**: The objective of the research was to determine the effects of the “Body Workout” group exercises on morphological characteristics of female university students. **METHODS**: The sample of research participants was made up of female university students from the Faculty of Sports and Physical Education in Novi Sad that were actively included in the workout program. The total sample participating in the research comprised of 52 participants, 25 of which comprised the experimental group (22.11±0.89 years; 58.72±7.71 kg; 166.11±5.01 cm) while the remaining 27 of them comprised the control group (22.45±0.78 years; 61.51±9.21 kg; 169.51±5.04 cm). The experimental group followed the “Body Workout” model and the control group followed the regular curriculum for the university course of Fitness. **RESULTS**: According to the research results, significant effects were determined in the transformation of morphological characteristics among the members of the experimental group on the level of the entire system and the individual differences could be perceived in the final measuring in variables Circumference of the lower leg and Skinfolds between the members of the experimental group. **CONCLUSION**: It was determined that the experimental program of aerobic exercises produced the greatest effects on the reduction in subcutaneous fatty tissue.

### O19 The influence of pre-adolescent physical activity over trait of emotional intelligence, optimism, and pessimism

#### Alexandru Boncu (alexandru.boncu@e-uvt.ro)

##### Faculty of Physical Education and Sport, West University of Timisoara, Romania

About 13% to 20% of youth suffer from mental health disorders at least once in a lifetime. Although previous studies have explored the relationship between physical activity and mental health, little is known about the relationship between psychological factors leading to mental health problems and physical activity in pre-adolescents. **PURPOSE**: First objective was to compare two groups of pre-adolescents (engaged in organized weekly physical activity vs. not engaged in organized weekly physical activity) on a set of trait emotional intelligence factors (emotionality, well-being, self-control, sociability) and expectation for the future (optimism, pessimism). The second objective was to see the variance explained by physical activity on the same set of variables. **METHODS**: Five hundred and twelve pre-adolescents (55.9% of boys) participated in the study. Participants were asked to complete a series of questionnaires along with the question: Are you involved weekly in an organized physical activity? **RESULTS**: We found statistically significant differences in all variables analyzed between the group engaged in organized weekly physical activity compared to the group without physical activity practice. Physical activity explained the variance of several variables: emotionality, well-being, optimism, pessimism. **CONCLUSION**: Physical activity has proved to be an important factor not only directly for mental health problems but also indirect for psychological factors important for mental health.

### O20 Catastrophizing pain, disability, and depression in patients with chronic low back pain

#### Elena Sirbu^1^, Roxana-Ramona Onofrei^2^, Ana-Maria Vutan^1^

##### ^1^Faculty of Sport and Physical Education, West University of Timisoara, Romania; ^2^Faculty of Medicine, “Victor Babes” University of Medicine and Pharmacy of Timisoara, Romania

###### **Correspondence:** Elena Sirbu (elena_sarbu@yahoo.co.uk)

Chronic low back pain (CLBP) is defined as low back pain lasting more than 12 weeks affecting more than 50% of the general population. It is estimated that more than 70% of the population experience at least one episode of lower lumbar pain, at a certain moment of life. **PURPOSE**: The main purpose of this study was to investigate the pain intensity, identify the disability and depression levels in patients with chronic back pain and to correlate these variables. **METHODS**: 20 patients with CLBP completed the Pain Catastrophizing Scale, the Visual Analog Scale, the Oswestry Disability Questionnaire and Beck's Depression Inventory. The associations among pain intensity, disability, depression and catastrophizing were done by using MedCalc Statistical Software 17.9.7. **RESULTS**: The mean age of patients included in this study was 42 ± 9.14 and the mean disease duration (years) – 2.1 ± 1.62. The majority (70 %) of the participants were low catastrophizing to pain with low-intensity pain, disability and depression levels. Increased levels of catastrophic thinking to pain correlate significant with disability levels (r= 0.5, p=0.02) and depression intensity (r=0.71, p<0.001). **CONCLUSION**: A multidisciplinary approach is needed for patients with CLBP and should include physical, mental and social evaluation in order to offer an optimal treatment.

### O21 Physiological demands of junior soccer players of three different positional roles

#### Marko Erceg, Sasa Krstulovic, Goran Kuvacic

##### Faculty of Kinesiology, University of Split, Croatia

###### **Correspondence:** Goran Kuvacic (gorkuv@kifst.hr)

**PURPOSE**: This investigation aimed to evaluate the physiological demands of junior (U-19) soccer players of three different positional roles. **METHODS**: Junior soccer players (body height 181.9 cm; body weight 75.1 kg; BMI 22.7 kg/m^2^) were categorized in three positional roles as follows: midfielders (n=8), defenders (n=8), and attackers (n=6). For this study, several physiological parameters were assessed during and after the incremental exercise test. ANOVA with Tukey-Kramer Unequal N HSD posthoc test was used to determinate differences between team positions. **RESULTS**: The differences in the heart rate values at VO2max between the midfielders and defenders were statistically significant (p <0.05) as well as between the midfielders and the attackers. Midfielders had significantly higher values of relative VO2max (65.63±3.58 ml/min/kg) than defenders (62±5.06 ml/min/kg). The midfielders had higher values in all ventilation parameters assessed when compared to defenders and attackers. We found these differences between midfielders and the attackers in the forced expiratory volume in one second (FEV1; p <0.05). Furthermore, differences between the same groups exist in peak expiratory flow (PEF; p <0.01). Also, defenders had higher values when compared to attackers in PEF (p <0.05). No differences were found in resting and blood lactate 1-min after between observed groups. **CONCLUSION**: These results are consistent with previous research. Given the specificity of their position, midfielders have higher values of ventilation and spiroergometric parameters as expected.

### O22 Working in sports organizations: contemporary sociological perspectives and issues

#### Tea Gutovic^1^, Renata Relja^2^, Toni Popovic^2^

##### ^1^Faculty of Economics, University of Split, Croatia; ^2^Faculty of Humanities and Social Sciences, University of Split, Croatia

###### **Correspondence:** Tea Gutovic (tgutovic@gmail.com)

Sociology understands sport as a social phenomenon influenced by and influencing various social structures as well as shaping the lives of individuals involved in sports activities. There is almost no person in the modern world that is not somehow connected to the sports industry, as an active participant, player, coach, manager, or as a passive observant, consumer, part of the crowd. **PURPOSE**: To analyze working in the sport on a micro and macro sociological level, considering aspects of social structure and identity formation. **METHODS**: We systematically reviewed existing literature and research about the sports industry, sociology of work, entrepreneurship and education. Using the descriptive research method of the key theoretical concepts and empirical results in these disciplines, we constructed specific perspective of sport as a form of work in post-industrial society following its basic principles of flexibility, change, and innovation. **RESULTS**: On a macro level, working in a sports organization should be considered as a specific profession, within the duality of structure that both empowers and constraints it. On a micro level, we consider the processes of shaping athletic and coach identity and their mutual interactions. **CONCLUSION**: We argue that sport has to be investigated as a form of entrepreneurship and sports identity formed based on the factors of entrepreneurial culture.

### O23 Differences in strength dimensions between students of kinesiology and students of mathematics

#### Josip Cvenic (jcvenic@foozos.hr)

##### Faculty of Education, Josip Juraj Strossmayer University of Osijek, Croatia

Prescribing training intensity and volume is a key problem when designing resistance training programs. **PURPOSE**: The aim of the study was to determine the differences between students of kinesiology and students of mathematics with respect to gender in some dimensions of strength. **METHODS**: The sample consisted of 50 students of kinesiology from Faculty of education (37 males age 20.10±1.72; BH 181.8±6.0; BW 78.03±10.11 and 13 females age 19.07±1.25; BH 167.6±9.24; BW 59.92±7.82) and 62 students from the Department of mathematics (31 males age 18.92±0.67; BH 178.50±6.46; BW 78.43±22.24 and 31 females age 18.6±0.56; BH 165.9±7.2 and BW 60.56±10.49). As a measurement of muscle strength, a sample of variables was comprised of five tests: 1 RM bench press (1RMBP), 1 RM squat (1RMS) and 1-minute sit-up test (1SU), relative bench press (RBP=1 RMBP/BW) and relative squat (RS=1 RMS/BW. The basic descriptive parameters were calculated and the t-test was used for determining the difference between the observed variables. **RESULTS**: The significance of differences between the groups and gender was observed in all variables (p<0.00). **CONCLUSION**: The findings may contribute to a better understanding of the factors responsible for strength in the population of athletes (students of kinesiology) and non-athletes (students of mathematics), i.e. they may be useful to PE teachers to help in the planning the best program and training load distribution for their students.

### O24 Gender differences in unilateral horizontal drop jump performance in children

#### Marijo Bakovic, Sanja Ljubicic, Ljubomir Antekolovic

##### Faculty of Kinesiology, University of Zagreb, Croatia

###### **Correspondence:** Marijo Bakovic (marijo.bakovic@kif.hr)

There are a lot of studies about human (children and adults) bilateral vertical drop jump performance. On the other hand, there are not enough studies about unilateral horizontal drop jump performance. **PURPOSE**: To investigate the nature of force-kinetics gender differences during unilateral horizontal drop jump performance in children. **METHODS**: Forty-nine children (23 boys and 26 girls; age 8 and 9 years) performed 3 trials of unilateral horizontal drop jump performance on take-off leg. Data acquisition was done using a force platform (Quattro Jump 9290CD, Kistler, Switzerland). The height of drop jump was 15 cm, while the distance of the starting point to the middle of the force platform was 110 cm. Maximum force in eccentric and concentric muscle activity, as well as the maximum rate of force development were analyzed using body weight unit of measurement. T-test for the independent samples was used to calculate the statistical significance of gender differences. **RESULTS**: Although boys produced slightly higher maximum force in eccentric and girls in concentric muscle activity, the differences were not statically significant. The maximum rate of force development also showed no statistical difference. **CONCLUSION**: The results of this study showed no gender differences in force-kinetics variables between 8- and 9-year old children during unilateral horizontal drop jump performance. In further studies, it should be analyzed in older children closer to puberty.

### O25 Reliability of newly developed tests of pre-planned and non-planned agility for older persons: a pilot study

#### Ivan Peric^1^, Miodrag Spasic^2^, Damir Sekulic^2^

##### ^1^Faculty of Dental Medicine and Health, Josip Juraj Strossmayer University of Osijek, Croatia; ^2^Faculty of Kinesiology, University of Split, Croatia

###### **Correspondence:** Ivan Peric (iperic@fdmz.hr)

Studies in the field of agility have identified two different forms of agility, pre-planned-agility (PPA) and non-planned-agility (NPA). **PURPOSE**: The aim of this research was to examine the reliability of newly constructed tests of reactive and pre-planned agility for elderly persons. **METHODS**: Ten healthy volunteers (6 women and 4 men; age 61.10 ± 4.4 years; body height 166.90 ± 7.82 cm; body weight 77.63 ± 12.19 kg) participated in this pilot study throughout test-retest study design. Respondents were tested on newly constructed tests of reactive and pre-planned agility twice over two days. Tests involved several stop-and-go movement patterns with (for NPA), or without (for PPA) reaction to visual stimuli. **RESULTS**: The reliability of the PPA test was high (Intraclass correlation: 0.83; Coefficient of variation: 3%), while the reliability of NPA test was somewhat lower but still acceptable (Intraclass correlation: 0.52; Coefficient of variation; 6%). Pearson's correlation between PPA and NPA was numerically low (r = 0.51, p = 0.19) indicating that reactive- and pre-planned-agility should be observed as independent qualities sharing less than 25% of the common variance. **CONCLUSION**: The results of this pilot study indicated appropriate reliability of newly developed PPA and NPA tests.

### O26 Attitudes of pre-service teachers on the acquisition of competencies for teaching physical and health education

#### Biljana Trajkovski, Petra Pejic Papak, Tena Pejcic

##### Faculty of Teacher Education in Rijeka, University of Rijeka, Croatia

###### **Correspondence:** Biljana Trajkovski (biljana.trajkovski@uniri.hr)

The task of the course Kinesiological Methodics is to introduce the students, future teachers, to the legalities within the physical and health care area and to train them for practical, a creative performance of the teaching process and other processes of physical exercise with the pupils. The aim of this research was to examine the attitudes on the acquisition of competencies and the students' satisfaction with the teaching of Kinesiological Methodics during the studies as well as their opinion on their qualification for carrying out the teaching of primary education classes. A questionnaire was conducted on a sample of 136 students enrolled at the Faculty of Teacher Education. The results point to the pre-service teachers' attitude that the knowledge gained in the courses of Kinesiological Methodics will be helpful in their future occupation (F = 7.37, p <0.05). However, they also stated the importance of continuous training upon the completion of the studies (F = 5.56, p <0.05). It is concluded that the pre-service teachers show the tendency of satisfaction increase with the teaching of Kinesiological Methodics in senior years of study, but at the same time it is concerning that in more senior years of study there appears to exist a decrease in the self-assessment of their own competence for performing the teaching of primary education classes.

### O27 Relations between motor skills and health-related quality of life at 7-8 years old children

#### Szabolcs Halasi^1^, Josip Lepes^1^, Visnja Djordjic^2^, Damjan Jaksic^2^, Ferenc Ihasz^3^, Robert Papp^1^, Nevenka Zrnzevic^4^, Andrea Zivkovic-Vukovic^5^

##### ^1^Hungarian Language Teacher Training Faculty in Subotica, University of Novi Sad, Serbia; ^2^Faculty of Sport and Physical Education, University of Novi Sad, Serbia; ^3^Faculty of Health Sciences, University of Pecs, Hungary; ^4^Teacher Training Faculty, University of Pristina-Kosovska Mitrovica, Serbia; ^5^Faculty of Education, University of Novi Sad, Serbia

###### **Correspondence:** Szabolcs Halasi (sabolc.halasi@magister.uns.ac.rs)

Hypokinesia has a negative impact on motor skills, self-confidence, well being, friendly relations, and academic achievement. **PURPOSE**: The aim of the research was to characterize the relations between Health-related quality of life (HRQOL) and motor skills at early school age children in Northern Serbia. **METHODS**: The study included 214 children (113 boys, 101 girls) aged 7-8 years. Stratified random sampling was used in this cross-sectional study. Measuring HRQOL was with parent version of Kidscreen-27 questionnaire and evaluating the motor skills was with Test of Gross Motor Development-2 which was for the first time used in Serbia. **RESULTS**: With Spearman’s rank correlation significant positive relations of HRQOL domains, Physical Well-being (r=0.44; p≤0.01), Psychological Well-being (r=0.31; p≤0.01), School Environment (r=0.29; p≤0.01) with Locomotor subtest and positive correlation between Gross Motor Quotient with domain, Physical Well-being (r=0.31; p≤0.01) at boys, were established. **CONCLUSION**: Boys who achieved better results on tests of motor skills are physically more active, full of energy, enjoy life, emotionally balanced and feel better at school.

### O28 Effects of isoinertial training on the physical fitness of junior basketball players

#### Marko DM Stojanovic^1^, Jana Izovska^2^, Mladen Mikic^1^, Bogdan Belegisanin^1^, Aleksandar Karac^1^, Vuk Stevanovic^1,3^

##### ^1^Faculty of Sport and Physical Education, University of Novi Sad, Novi Sad, Serbia; ^2^Faculty of Physical Education and Sport, Charles University in Prague, Czech Republic; ^3^Institute for Medical Research, University of Belgrade, Serbia

###### **Correspondence:** Marko DM Stojanovic (marko.ns.stojanovic@gmail.com)

**PURPOSE**: The aim of this research was to determine the effects of an 8-week isoinertial training on the physical fitness of young basketball players. **METHODS**: Twenty-four male junior basketball players, (17.58±0.50 years) were divided into experimental (ECC; n=12) and control group (CON; n=12) which took strength training exercises on isoinertial training device and classic isodynamic strength training with free weights, respectively. During the experimental program, the ECC and CON groups had 12 equal-volume strength training sessions. A two-way analysis of variance was used to test the effects of isodynamic and isoinertial strength training on physical fitness. **RESULTS**: The findings revealed that isoinertial training brought about significantly better results in the squat jump (SJ), Countermovement jump (CMJ) and vertical jump (VJ), as well as in the 5m sprint test and agility tests (T-test and 505 test). No statistically significant differences were found in isometric strength and 20m run. **CONCLUSION**: In comparison with the traditional isodynamic training, isoinertial training proved superior in developing young basketball players explosive strength, speed and agility and likely should be regarded as a preferred method of strength training.

### O29 Female athletes in super league of Serbia: degree of athlete's satisfaction, the motivation for sports and competitive anxiety

#### Jovana Trbojevic^1^, Jelica Petrovic^2^

##### ^1^Matica Srpska, Novi Sad, Serbia; ^2^Department of Psychology, Faculty of Philosophy, University of Novi Sad, Serbia

###### **Correspondence:** Jovana Trbojevic (jovana.trbojevic88@gmail.com)

Athlete's satisfaction, the motivation for sports and competitive anxiety play an important role in sports achievement. **PURPOSE**: Determining the degree of athlete's satisfaction, the motivation for sports and competitive anxiety of female athletes who participate at the highest sports level in Serbia. **METHODS**: Thirty-six female athletes (handball players=12; volleyball players=11, soccer players=13) who participate at the highest sports level in Serbia (age: 16-27), completed Athlete's Satisfaction Questionnaire, Sport Motivation Scale-2 (dimensions: intrinsic/integrated motivation; extrinsic/introjected motivation; identified motivation and motivation), Competitive State Anxiety Inventory-2 (dimension: somatic and cognitive anxiety). **RESULTS**: Descriptive statistics show that female athletes achieve above theoretical average scores on athlete’s satisfaction (Mean=19.4), intrinsic/integrated motivation (Mean=18.33), identified motivation (Mean=10), somatic anxiety (Mean=12.94), and cognitive anxiety (Mean=13.69). Results of ANOVA show that there are significant differences between female handball, volleyball and soccer players in satisfaction (F(2.34)=3.608, p=0.039), intrinsic/integrated motivation (F(2.35)=4.016, p=0.027) and identified motivation (F(2.35)=4.192, p=0.024). Post hoc, Turkey HSD test shows that female soccer players are significantly more intrinsically motivated to do sports that handball players. On the other hand, female handball players are more satisfied then volleyball players. **CONCLUSION**: These results contribute to the theoretical and practical knowledge of elite female players and their individual dispositions have a significant role in sports results and the development of the sports itself.

### O30 Skeletal muscle tensiomyography: results from childhood to late adulthood

#### Bostjan Simunic, Rado Pisot

##### Science and Research Centre Koper, Institute for Kinesiology Research, Slovenia

###### **Correspondence:** Bostjan Simunic (bostjan.simunic@zrs-kp.si)

Tensiomyography is a noninvasive method to assess skeletal muscle mechanical contractile properties. **PURPOSE**: While there are numerous data on the fiber type composition of skeletal muscle in adults, little is known about the changes in fiber type composition and contractile properties in muscles during maturational growth. Furthermore, master athletes (MA) maintain high levels of physical activity during aging and are a unique population to study intrinsic aging. **METHODS**: Using tensiomyography we measured contraction time (Tc) in vastus lateralis (VL), gastrocnemius medialis (GM) and biceps femoris (BF) in children (N=107), adult nonathletes (N=379) and MA (N=327). Children were longitudinally followed for 6 yearly assessments, while adults were assessed in a cross-sectional design. **RESULTS**: Children had higher myosin heavy chain 1 proportion than adults until they reached 10 years. Children that were regularly involved in sport had lower BF Tc than sedentary children, but only after the age of 12, (13%; p<.001) and not in VL. In adults, we found age-related slowing of all observed muscles, where endurance MA had the longest Tc than non-athletes, while power MA had the shortest Tc. Furthermore, our data revealed longer Tc in endurance MA than in non-athletes with age, indicating that preferring regular endurance sports activity might accelerate the age-related slowing of skeletal muscles. **CONCLUSION**: The results demonstrate the importance of regular sports exercise for skeletal muscle development in childhood and adulthood.

### O31 Family and the creation of a child’s physical (body) capital

#### Sasa Pisot^1^, Rado Pisot^1,2^

##### ^1^Science and Research Centre Koper, Slovenia; ^2^Faculty of Sport, University of Ljubljana, Slovenia

###### **Correspondence:** Sasa Pisot (sasa.pisot@zrs-kp.si)

Parent’s role in supporting a child in the physical activity (PA) appears to be very important from the point of creation of an "embodied cultural capital" or physical (body) capital that begins at birth and lasts during all the processes of socialization. **PURPOSE**: From the point of physical capital’s accumulation, parent’s support and (non)engagement of parents in the child’s PA and his motor efficiency was examined in the frame of the project “Analyzing fundamental motor pattern, skeletal and muscle adaptation on specific sedentary lifestyle factors amongst 4 and 7-year-old children”. **METHODS**: Quantitative (questionnaires n = 90, a child’s motor efficiency test battery) and qualitative (n = 60 semi-structured interviews) analysis were carried out with parents and children. **RESULTS**: Four different groups on children's motor efficiency and family lifestyle showed that family praxis depending on many factors, including the socioeconomic status of the family and parent's "time free from economic necessity". **CONCLUSION**: Non-engagement of parents in the child’s PA could be treated as a lost time which gives even greater responsibility to parents to provide time for accumulation of body capital. Mothers are most likely the parent which ensure expected healthy family lifestyle. Since providing healthy nutrition for children falls within the mandate of maternity, the PA of children still mostly remains in the domain of fathers.

### O32 The factors of engaging in the hiking section as a sports recreation activity in high school

#### Ivan Vrbik, Andrea Vrbik, Mirna Andrijasevic

##### Faculty of Kinesiology, University of Zagreb, Croatia

###### **Correspondence:** Ivan Vrbik (ivan.vrbik@gmail.com)

**PURPOSE:** Managing and understanding the mechanisms underlying enrollment of students in extra-curricular activities could alleviate teacher’s (i.e. expert’s) approach to the students and facilitate their engagement in mentioned activities. Therefore, the main goal of this paper was to establish the factors influencing the choice of enrolling in the particular extra-curricular hiking section as a recreational activity. **METHODS:** The study was conducted on a sample of 40 high school students (15.3±0.66 years old). Anthropometrical variables (body height, body weight, BMI, body fat, and body muscle) were measured and students completed a short recreational preference questionnaire. Frequency tables were calculated for asked questions and after comparative analyses were made. Correlation between variables was done to establish the existence of significant correlations. **RESULTS:** Results show a negative correlation (r=-0.42; p<0.05) between attending PE classes and body fat and relatively high correlation (r=0.63; p<0.05) between attending PE classes and preference to outdoor activities, namely hiking. According to the results of correlation, the greatest influence on the interest to join hiking section have students' preference for outdoor activities (r=0.43; p<0.05). **CONCLUSION**: An increase in the outdoor time expenditure, firstly initiated by PE teachers (and hopefully later adopted as a way of frequent independent recreational and leisure time activity) could be the spot-on strategy for the increase in physical activity levels in school-aged children during after school hours.

### O33 “Sports School” 30 years old kinesiology model - a connection between science and practice

#### Dusan Stupar^1^, Dejan Madic^2^, Gustav Bala^2^, Boris Popovic^2^, Dragan Grujicic^3^, Dusan Kulic^4^, Aleksa Madic^2^

##### ^1^Faculty of Sport and Tourism, Educons University, Serbia; ^2^Faculty of Sport and Physical Education, University of Novi Sad, Serbia; ^3^Sports School, Novi Sad, Serbia; ^4^Elementary school "Bratstvo i jedinstvo", Vrbas, Serbia

###### **Correspondence:** Dusan Stupar (dusan9stupar@gmail.com)

**PURPOSE**: In recent years, there has been an increasing number of sports schools for children of pre-school and younger school age. This is an important fact because the children are exercising from the earliest ages, in the aim to develop their motoric, functional and psycho-physical abilities. The purpose of this paper is to describe the model, methods, principles and organizational structure of the "Sports School". **CASE PRESENTATION**: "Sports School“, held at the Faculty of Sport and Physical Education in Novi Sad, has existed for 30 years and it is one of the pioneers of this kind of activity for children. School is an example of a good combination of science and practice. All employees are highly educated professionals. The work program applies modern methods of exercise based on scientific research. These researches have shown that the positive effects on the transformation of certain anthropological dimensions have been achieved at different time points and with different generations of children. **CONCLUSION**: This model proved to be successful. He can be applied to all sports clubs and schools that work with children of preschool and early school age.

### O34 The food and physical activity carousel - a choice for healthy living among Timisoara west university female college students

#### Nicoleta Silvia Mirica, Cerasela Domokos, Martin Domokos, Eugen Bota, Cristian Negrea, Adrian Nagel

##### Faculty of Sport and Physical Education, West University Timisoara, Romania

###### **Correspondence:** Nicoleta Silvia Mirica (nicoleta.mirica@e-uvt.ro)

Good nutrition and physical activity are the main discussed lifestyle determinant factors. **PURPOSE**: The evaluation of nutritional and physical activity habits of the West University female students, participants at the general course of sports and physical activity. **METHODS**: 81 female volunteers (age: 20 ± 0.77 years) filled 2 questionnaires regarding nutrition and physical activity. A comparison with the Romanian guidelines has been performed. **RESULTS**: The questionnaire focused on monthly consumption of both main food groups and comfort foods and physical activity habits. The data (GraphPad Prism 5.0 and Microsoft-Excel 2007, statistically significance was considered for values of p<0.05) revealed extremely significant differences for all evaluated parameters. For all food groups the results indicated an extremely low monthly consumption comparing to the guidelines (fruit, vegetables/ dairy/comfort food: 22.06 ± 2.12/23.21 ± 2.11/21.32 ± 2.2 vs. 60-150 portions/month, p<0.001; meat and alternatives: 12.73 ± 1.46 vs. 60- 90 portions/month, p<0.001). Physical activity was at one third compared with the guidelines (p<0.001). **CONCLUSION**: The results indicated that youth is not enough to live a healthy life, therefore, is mandatory to tailor great improvements in both nutrition and physical activity in order to remain healthy for a longer period of time.

### O35 E-sports: definition and social implications

#### Dino Vukusic^1^, Marko Marelic^2^

##### ^1^Institute of Social Sciences “Ivo Pilar”, Zagreb, Croatia; ^2^School of Medicine, University of Zagreb, Croatia

###### **Correspondence:** Dino Vukusic (dino.vukusic@pilar.hr)

The final phase of the gaming industry’s development brought with it another phenomenon – e-sport. “Electronic sport” presupposes playing video games in a competitive setting, with emphasis on increased “institutionalization" of gaming activity through the organization of e-sports teams and official international competitions. In order to define e-sports, it's important to note that not every activity of playing video games can be described as an e-sport, but every e-sport is essentially playing video games. **PURPOSE:** This paper aims to present several dimensions of the "electronic sport" phenomenon, review the existing research in this field, compare various aspects of e-sports and "traditional" sports, and discuss the formation of a new subcultural group gathered around video games. **CASE PRESENTATION:** The key task of this paper is to examine the distance between e-sports and “traditional” sport and frame the socio-economic scale of the phenomenon. The final part of the paper will concentrate on examining the emergence of gaming culture, primarily through the prism of post subcultural theories. **CONCLUSION:** The interest in the phenomenon of electronic sport in all social sciences, including sociology, has increased over the past several years and terms and definitions used to describe it are still under construction. Therefore, a theoretical paper clarifying key characteristics is a vital step towards a further scientific upturn in studying e-sport.

### O36 Changing trend of motor abilities in professional male and female folk dancers

#### Goran Oreb^1^, Meta Zagorc^2^, Gordana Furjan-Mandic^1^

##### ^1^Faculty of Kinesiology, University of Zagreb, Croatia; ^2^Faculty of Sport, University of Ljubljana, Slovenia

###### **Correspondence:** Goran Oreb (goran.oreb@gmail.com)

Following a detailed analysis of motor abilities of professional folk dancers in 2012 (Oreb et al., 2013), the results of certain male and female dancers from the mentioned testing were compared with outcome values from September 1992, December 1992 and July 1993. **PURPOSE:** To determine the changing trend of motor abilities in male and female professional folk dancers within 20 years. **METHODS**: The sample of examinees included 6 male dancers (aged between 39 and 54) with between 21 and 33 years of dancing experience, and 9 female dancers (aged between 38 and 55;) with between 21 and 37 years of experience. The results were obtained upon comparing test results from September 1992 (No. 1) – initial testing; December 1992 (No. 2) – transitive testing; July 1993 (No. 3) – final testing, and September 2012 (No. 4) – initial testing. The measured results were analyzed via ‘trend analysis’. **RESULTS:** It should be emphasized that this analysis evaluated innate motor abilities that first deteriorate with age, but are relevant for dancing a success. The obtained results indicate a stagnation of almost all motor abilities. **CONCLUSION:** Within the analyzed 20 years, male and female dancers actively participated in the work, where the demands of the stage remained unchanged, while the dancers got older, which poses a chronic problem for training programming.

### O37 The evaluation of postural stability during the menstrual cycle in active women

#### Nese F. Sahin, Ozkan Guler, Azize Diedhiou, Gulfem Ersoz

##### Faculty of Sports Science, University of Ankara, Turkey

###### **Correspondence:** Ozkan Guler (ozkanguler@msn.com)

Several studies have demonstrated a higher incidence of sports-related injuries among female athletes compared to male counterparts. **PURPOSE**: The purpose of this study was to evaluate the change in static balance ability during the menstrual cycle among young active women. **METHODS**: The subjects were young active healthy women (n=19, 20.4±1.2 years) with regular exercise and a regular menstrual cycle. Menstrual cycle was divided into 3 phases. early menstrual phase, the follicular phase and mid-luteal phase of the menstrual cycle. The Biodex SD Balance System was used to determine to static balance ability. Static balance ability was measured by the postural stability index protocol. All subjects were evaluated with their eyes open and double leg without footwear. The subject was asked to step on the platform of the BSS and adopt a comfortable position while maintaining a stand position. To become familiar with postural stability testing on the BBS, subjects performed three practice testing sessions within a week. **RESULTS**: The results of this study indicate that there were no differences in anterior-posterior sway, medio-lateral sway and postural stability index among three phases in active young woman. **CONCLUSION**: In the consequence of this study, it has been found that postural stability index has not changed among three phases in active young woman.

### O38 Differences in game-related statistics for winning and losing teams related to changes in basketball rules

#### Mladen Mikic^1^, Igor Vuckovic^2^, Marko Stojanovic^1^

##### ^1^Faculty of Sport and Physical Education, University of Novi Sad, Serbia; ^2^Faculty of Physical Education and Sport, University of Banja Luka, Bosnia and Herzegovina

###### **Correspondence:** Mladen Mikic (mmmikac@gmail.com)

In 2014 FIBA has made a change to the official rules to speed up game-pace. The most important change is that after an offensive rebound shot clock shall be reset to 14 seconds. **PURPOSE**: To determine the differences in game-related statistics before and after changes of basketball rules. **METHODS**: Data were obtained from official websites of men's 2014 World Cup (38 games) and 2016 Olympic Tournament (76 games). Data consist of 15 parameters, and teams have been separated by success in the game. **RESULTS**: Winning teams at OT had significantly more ball possessions (p=0.050) and assist (p=0.002) in relation to the winning teams from WC, who had more turnovers (p=0.025). Losing teams at OT in comparison to teams from WC had significantly more ball possessions (p=0.039), successful 2-point shots (p=0.037), defensive rebounds (p=0.004) and unsuccessful free throws (p=0.039). Regardless of success, teams from the OT achieved a significantly higher number of ball possessions (p=0.005), defensive rebounds (p=0.014) and assists (p=0.004). **CONCLUSION**: Results of the analysis show that after changing the rules number of ball possessions has increased, the game has accelerated. Coaches should select and prepare players to be ready to play faster, for basketball in the future.

### O39 Sources of enjoyment and goal orientation among youth fitness members

#### Okan B. Micoogulları^1^, Nazif Kutay Erden^2^, Gunay Yıldızer^3^

##### ^1^Physical Education & Sports Department, Nevşehir Hacı Bektaş Veli University, Nevşehir, Turkey; ^2^Higher Education Council, Ankara, Turkey; ^3^Sport Science Faculty, Eskişehir Technical University, Eskişehir, Turkey

###### **Correspondence:** Okan B. Micoogulları (okanmicoogullari@gmail.com)

**PURPOSE:** The objectives of this study were to examine whether there are achievement goal orientation differences regarding enjoyment of and to assess which sources of enjoyment differentiate fitness members of different achievement goal orientation profiles. **METHODS:** Female (N=28) and male (N=77) fitness members aged 15 to 18 years completed measures sources of enjoyment in sport, and Task and Ego Orientation in Sport. **RESULTS:** Results of ANOVA revealed that those with high task/high ego, high task/moderate ego and moderate task/low ego profiles have significantly higher levels of enjoyment of fitness as compared to those with low task/moderate ego profiles. Analysis showed that four groups of members could be described by two discriminant functions. High task/high ego group is placed on the positive side of the function “achievement with intrinsic motivation” and they showed the greatest enjoyment of fitness center in other-referenced competency and recognition, effort expenditure, self-referenced competency, and positive parental involvement. Low task/moderate ego group significantly stands out on the positive side of the "achievement without effort" function and showed the greatest enjoyment only in other-referenced competency and recognition. **CONCLUSION:** This research gives an understanding of a conceptual link between achievement goal orientation and sources of enjoyment in fitness members.

### O40 Differences in competitive karate athletes within anthropometric characteristics

#### Sandra Vujkov^1^, Milenko Jankovic^2^, Zoran Milic^1^

##### ^1^College for Vocational Education of Preschool Teachers and Sport Coaches, Subotica, Serbia; ^2^College for Vocational Education of Preschool Teachers, Novi Sad, Serbia

###### **Correspondence:** Sandra Vujkov (sandravujkov@vsovsu.rs)

Contemporary Kata and Kumite disciplines have distinguished competition requirements. Top performance and sporting results in both disciplines are conditioned with a high level of technical performance, and anthropometric predispositions among others. **PURPOSE:** To investigate differences between Kata and Kumite athletes in chosen anthropometric parameters. **METHODS**: Forty male international level karate athletes (21 kata; age 21.0 ± 4.1 years; BMI 22.6 ± 1.7 kg/m^2^ and 19 Kumite athletes age 21.7 ± 3.0 years; BMI 22.7 ± 1.5 kg/m^2^) participated in cross-sectional study. Overall, 23 anthropometric variables (five longitudinal; four transversal; five circumferences; and nine skinfolds) were taken according to the International Biological Program standards. MANOVA and ANOVA were used to obtain the differences between competitive disciplines, with the significance level set to p ≤ 0.05. **RESULTS:** Kumite athletes showed to be significantly heavier, taller, had longer extremities (p ≤ 0.01) and with more pronounced circumference (p ≤ 0.05). Kata competitors had more pronounced subcutaneous adipose tissue (p < 0.05). **CONCLUSION:** Kumite longitudinal dimensionality shows a possibility of reaching opponents from greater distances, and Kata athletes need firm stances with a lower center of gravity. Kumite has additional weighing categories. Greater adipose tissue in kata athletes seems to have no fundamental reflections on performance. Knowledge in findings of athletes' characteristics should be implemented in sports performance planning.

### O41 The influence of motor abilities and morphological characteristics on the success in kayaking

#### Nikola Prlenda, Ivor Kajfez, Ivan Oreb, Zoran Spoljaric, Mario Jankovic

##### Faculty of Kinesiology, University of Zagreb, Zagreb, Croatia

###### **Correspondence:** Nikola Prlenda

**PURPOSE:** Motor abilities and morphological characteristics are some of the main factors influencing the result in professional kayakers. The question is how much are these abilities influencing the results in adult recreational sportsmen, i.e. is the quantity of acquired knowledge in basic rowing technique more important factor in the final test result. **METHODS**: The research was conducted on a sample of 27 female and 47 male students of the Faculty of Kinesiology in Zagreb. The average age was 21, 86 for female, and 21, 68 years for male students. Predictor variables were morphological characteristics tests and 10 motor abilities tests. As a criterion variable 50-meter sprint test was used. The test was applied after the six-day basic kayak technique learning program (9 hours). **RESULTS:** Through regression analysis, it was determined that there is no significant influence of morphological characteristics and motor abilities on the success in 50-meter kayak rowing. **CONCLUSION:** The research hasn't shown a significant correlation of morphological characteristics and motor abilities with the results achieved in kayak 50-meter sprint test, and it can be assumed that the results in the aforementioned test were influenced by the level of achieved rowing technique knowledge. Such result shows also the accessibility of kayaking to the wider population of recreational sportsmen, not only to the physically prepared individuals.

### O42 How strongly related are health status and subjective well-being among the employees of the Hungarian National Ambulance Service and the Hungarian Defense Forces?

#### Attila Pandur, Rajmund Marton, Balint Banfai, Henrietta Banfai-Csonka, Bence Schiszler, Jozsef Betlehem, Balazs Radnai

##### Faculty of Health Sciences, University of Pecs, Hungary

###### **Correspondence:** Attila Pandur (attila.pandur@etk.pte.hu)

**PURPOSE:** The increasing social role and responsibility of both the National Ambulance Service (NAS) and the Hungarian Defense Forces (HDF) may impact the workers physical and mental condition, which then affects their work. The purpose of our research was to assess the quality of life of paramedics and soldiers along the factors influencing it. **METHODS:** The anonymous, self-filled questionnaire used for the cross-sectional study contained EuroQol scale, Beck depression and Briere PTSD questionnaire, SF-36 and analyzed with SPSS 20.0. **RESULTS:** In the research 98 NAS and 76 HDF personnel participated. The relationship between the years spent in work and the quality of life showed positive results for both groups (p=0.009). The Beck depression questionnaire revealed that NAS workers are more depressed than those who are serving in the HDF (p<0.05). In all dimensions of the SF-36 questionnaire, the paramedics performed worse and those who were workers for more than 11.5 years in both groups rated their quality of life worse (p<0.05). **CONCLUSION:** The respondents have been subjected to increased physical wear, they fall victim to subsequent physical deterioration, mental decline. The risk of all factors will gradually increase with age, as well as with the number of years spent working.

### O43 Comparative study on health behavior and burnout of healthcare professionals working in clinical and preventive health care in Hungary

#### Krisztina Deutsch, Adel Siric

##### Institute of Emergency Care and Pedagogy of Health, Faculty of Health Sciences, University of Pécs, Hungary

###### **Correspondence:** Krisztina Deutsch (kriszta.csokasi@freemail.hu)

**PURPOSE:** Research related to health emphasizes that health care workers appear as role models in their work. The analysis and comparison of health behavior and burnout among health professionals in clinical care and prevention in Hungary. **METHODS**: The quantitative cross-sectional study was carried out between 01.02.2016 and 30.11.2016. During convenience sampling, 293 health workers were involved in the sample. Data were collected with self-compiled questionnaires including standardized question groups (SF-36, Maslach Burnout Inventory). Data were analyzed with SPSS 20.0 program, descriptive statistical analysis, Chi-square test, two-sample T-test. **RESULTS:** The health behavior of prevention professionals and clinicians show significant differences in the field of smoking (p=0,003), physical exercise (p=0,003) and the nutritional status (p=0,001). Examining burnout, a significant difference was not found in the incidence of emotional exhaustion between the two groups, however, in case of depersonalization, frequency (p<0,001) and intensity (p=0,046) showed a difference. The frequency of decrease in personal performance did not show a difference between the two groups (p=0,118), while in the case of intensity the difference was significant (p=0,004). **CONCLUSION:** In the case of the compared factors, prevention professionals had better health behavior. The examination if the incidence of burnout showed a significant increase compared to previous national studies, which calls attention to the importance of prevention and mental health.

### O44 Prefrontal cortex oxygenation is more affected by posture than cognitive load in young and older adults

#### Uros Marusic^1,2^, Rado Pisot^1,3^

##### ^1^Science and Research Centre Koper, Slovenia; ^2^Alma Mater Europaea – ECM, Slovenia; ^3^Faculty of Sport, University of Ljubljana, Slovenia

###### **Correspondence:** Uros Marusic (Sasa.Pisot@zrs-kp.si)

Normal aging triggers alternations in postural control, from an automatically processed to a more cortically controlled one. **PURPOSE:** to evaluate a postural-cognitive dual-task paradigm in young and older adults using functional Near-Infrared Spectroscopy (fNIRS). **METHODS:** Twenty healthy subjects were recruited for this study: ten younger (mean age = 23y) and ten older (mean age = 72y). The oxygenation level of the prefrontal cortex was measured with fNIRS and postural control was monitored at the same time using a force plate during four different conditions: normal quite stance and tandem position in both, normal and in dual-task conditions. **RESULTS:** Cognitive task while standing was better performed in the younger adults, who gave more correct answers in both dual-task conditions (p<0.05) while there was no difference between either age groups in terms of body sway parameters (p>0.05) or calculated dual-task effects (p>0.05). Cerebral oxygenation values increased significantly between the single-task stance and single-task tandem (p<0.05), and single-task stance and dual-task tandem (p<0.05). **CONCLUSIONS:** Even though there was a general lack of aging effect, the better cognitive performance under motor-cognitive conditions in young compared to older adults were observed. The current results point out that prefrontal cortex is influenced more strongly by postural than cognitive load.

### O45 Rupture and reconstruction of the anterior cruciate ligament

#### Andjelka Knezovic Svetec^1,2^

##### ^1^Rehamed Offenbach, Offenbach am Main, Germany; ^2^Faculty of Kinesiology, University of Zagreb, Croatia

###### **Correspondence:** Andjelka Knezovic Svetec (angela.knezovic.svetec@gmail.com)

Over the last decade, there has been an increase in the number of people who are recreationally or professionally engaged in physical activity. The consequence of an increase in physical activity and sports resulted in the higher number of injuries of the lower extremities, especially knee injuries. The rupture of the anterior cruciate ligament (ACL) is a particularly severe injury that can have a negative impact on the level of physical activity and impair quality of life of an individual. ACL rupture can be career-threatening for professional athletes. The surgeons mainly use two types of surgical techniques for reconstructing anterior cruciate ligament, patellar tendon autograft, and hamstring-gracilis tendon autograft. Even though the anterior cruciate ligament reconstruction surgery is considered to be a successful procedure, the rate of return to the professional sport after ACL reconstruction is relatively low. The most important goal after ACL surgery is returning athletes to training activity and finally to resume pre-injury fitness level.

### O46 Does exercise influence brain chemistry changes in healthy adults? a systematic review

#### Dragana Zanini, Vesna Seper, Dejan Javorac, Valdemar Stajer, Sergej M. Ostojic

##### Faculty of Sport and Physical Education, University of Novi Sad, Serbia

###### **Correspondence:** Dragana Zanini (zadragupsi@yahoo.com)

**PURPOSE**: Neuroimaging become widely used, contemporary research tool with the potential to shed light on an old question like does physical exercise can reduce cognitive decline and prevent malfunctioned brain chemistry processes which underlie various age-related diseases? In this review, the focus is put on investigating researches that used magnetic resonance spectroscopy (MRS) to determine whether, and in what direction, exercise can alter healthy brain metabolites? **METHODS**: Search is conducted in databases “PubMed” and “Cochrane Library" using keywords "magnetic resonance spectroscopy" AND "exercise" AND "healthy adults" Search was restricted to articles published in the English language in 10 last years and conducted on humans. **RESULTS**: Following criteria, it was extracted 13 studies. After the vigorous acute bout, results indicate increased brain lactate and gamma-aminobutyric acid (GABA). Results are inconsistent considering N-acetyl-aspartate (NAA) and glutamate-glutamine (Glx) level. Findings of this review indicate insignificance alteration of NAA level if measured in occipital cortex, in contrast to an increased level in frontal cortex and posterior cingulate cortex. Although there is a notion that exercise has widespread effects on the brain, a specific region of interest (ROI) could be more sensitive to neurometabolites changes than global measures. **CONCLUSION**: Due to a small number of studies, with different methodologies, there is no definitive conclusion toward the question of the impact of physical activity on brain chemistry in healthy adults.

### O47 Is the smartphone app accurate enough for static posture analysis?

#### Drazenka Macak, Dragan Marinkovic, Aleksa Madic, Nikola Andric, Nebojsa Trajkovic

##### Faculty of Sport and Physical Education, University of Novi Sad, Serbia

###### **Correspondence:** Nebojsa Trajkovic (nele_trajce@yahoo.com)

The accurate description of body posture represents a topic of interest for the scientists. Due to the fact that most reliable methods are expensive and often reserved for clinical or laboratory use, it is necessary to found low-cost photographic methods of assessing standing posture with satisfactory reliability. **PURPOSE**: The aim of this narrative review is to summarize the validity and reliability of smartphones/tablets apps for assessing standing posture. **METHOD**: PubMed, Scopus, and SportDiscus databases were used to perform this study. The following keywords were used: Posture, mobile touch screen devices, application, reliability, validity. Two reviewers independently screened the articles, extracted relevant data and assessed the methodological quality of included studies. **RESULTS**: A total of 124 articles were screened for eligibility with 12 articles finally included for review. The current literature contains numerous limitations and lack of consistency for reliability. **CONCLUSION**: Although several studies showed mobile touch screen devices are valid and reliable, many factors have to be taken into account before it can be considered for wider use. Moreover, further research is warranted in order to develop guidelines for wise use of apps. Nevertheless, photographic-based apps showed one strong advantage, they provide a permanent and printable record of measurement.

### O48 Postural stability in people with and without patellofemoral pain

#### Dragan Marinkovic, Borislav Obradovic, Darinka Korovljev

##### Faculty of Sport and Physical Education, University of Novi Sad, Serbia

###### **Correspondence:** Dragan Marinkovic (marinkovic@uns.ac.rs)

Patellofemoral pain (PFP) is one of the common conditions of persistent anterior knee pain and is frequently reported during physical activity in both athletes and sedentary individuals. **PURPOSE**: To compare static postural stability in subjects with and without patellofemoral pain syndrome (PFP). **METHODS**: Twenty-seven PFP patients (8 women and 19 men; age 27.4±2.6 years; BMI 22.3±2.4 kg/m2) and 26 controls (13 women and 13 men; age 28.0±3.6 years; BMI 22.2±2.8 kg/m2) participated in this cross-sectional study. The data from force plates used to calculate the stability of body during 30s double-leg stand upright quietly with eyes open. Postural stability was quantified in terms of area, anterior-posterior (AP), medial-lateral (ML), and displacements of the center of pressure (COP). **RESULTS**: T-test shows that PFP patients had significantly greater (ES = 1.48; p = 0.00) displacement center of pressure (COP) compared with the non-injured individuals. This study also indicates that individuals with PFP demonstrated significantly larger oscillation of postural sway (ES = 0.866; p = 0.03) in the anterior-posterior (ES = 1.37; p = 0.00), and medial (ES = 1.38; p = 0.00) directions compared with those in healthy group. **CONCLUSION**: Results hint that subjects without PFP were significantly greater in different parameters of postural stability than PFP patients. Future studies should exam and expose PFP patients to dynamic balance conditions to develop PFP training programs.

### O49 Effects of creatine supplementation and heavy resistance training on strength and morphological characteristics in college athletes

#### Dusan Rakonjac, Mila Vukadinović Jurisic, Jelena Obradovic

##### Faculty of Sport and Physical Education, University of Novi Sad, Serbia

###### **Correspondence:** Dusan Rakonjac (rakonjac992@gmail.com)

**PURPOSE**: Creatine supplementation has become a popular supplement and common practice among all level athletes. The purpose of this study was to examine the influence of creatine monohydrate in combination with heavy resistance training on strength and morphology. **METHODS**: Fourteen male athletes (22.3±1.1) were randomly assigned to 2 groups: experimental (N=9), who consumed 20 g/day creatine (Cr) for 1 week and 5 g/day Cr for 7 weeks, and control (N=5). The experimental program consisted of four training sessions per week and lasted 12 weeks. Strength and anthropometric tests were taken before and after the experimental program. Four tests for strength assessment were used: bench press, squat, shoulder press, and pull-ups; for assessment of anthropometric parameters following variables were used: body weight, chest, waist and upper leg circumference, upper arm circumference-relaxed, upper arm circumference-flexed, abdomen, and m. triceps brachii skinfold thickness. **RESULTS**: There was a significant difference between experimental and control group in the bench press and squat (p<0.01), but no significant difference in shoulder press and pull-ups were observed; and there was also a significant difference between groups in upper leg circumference, upper arm circumference-flexed, and m. triceps brachii skinfold thickness (p<0.01). **CONCLUSION**: Creatine supplementation with heavy resistance training is beneficial in enhancing maximal strength and voluminosity in young college athletes.

### O50 From freshman to the second year: changes in body composition of college students

#### Vesna Seper, Nebojsa Nesic

##### College of Applied Sciences “Lavoslav Ruzicka”, Vukovar, Croatia

###### **Correspondence:** Vesna Seper (vesna.seper@vevu.hr)

Entering college and beginning of study can be very stressful and subjected to change. The changes usually include physical activity and nutrition pattern, and body mass. **PURPOSE:** To evaluate changes in body composition over a one-year study. **METHODS**: A total of hundred and one participants (65 women and 36 men; age 18.9 ± 0.73 years; body mass index [BMI] 22.7 ± 3.1 kg/m^2^) participated in this randomized longitudinal research. Body composition was evaluated by bioimpedance analyzer at baseline (one week after classes began) and at one-year follow-up. **RESULTS:** Overall difference between initial and final measurement was established (p = 0.008). Variables contributed to difference the most were weight (mean difference + 1.02 kg, p = 0.02), percent body fat (mean difference + 0.75%, p = 0.02), BMI (mean difference +0.29 kg, p = 0.04) and waist to hip ratio (mean difference + 0.01 points, p = 0.003). Changes in lean body mass and soft lean mass were insignificant (p ≥ 0.05). Differences were also found regarding sex for weight (p = 0.001), lean body mass (p = 0.002), soft lean mass (p = 0.003) and BMI (p = 0.002). **CONCLUSION:** Changes in body composition of students one year upon entering the college were significant, and different for men and women.

### O51 Optimizing ice slurry ingestion for endurance performance in the heat: a meta-analysis

#### Yang Zhang (yzhang68@crimson.ua.edu)

##### Faculty for Sport and Physical Education, University of Montenegro, Montenegro

**PURPOSE:** Ice slurry ingestion is a simple cooling intervention purported to improve endurance performance. Despite its popularity in the field, a recent meta-analysis suggested this intervention has no performance effect. The aim of the present meta-analysis was to determine the effect of ice slurry ingestion on endurance performance in the heat. **METHODS**: Data for this meta-analysis were retrieved from the PubMed. Effect sizes were calculated as the standardized mean difference (Hedges’ g), and meta-analyses were completed using a random-effects model. A method-of-moments meta-regression was used to determined confounding factors. **RESULTS:** Sixteen studies using randomized controlled trials with a total of 152 subjects were included. Improvement in endurance performance in the heat was moderate: g=0.60 (95% confidence interval, 0.34-0.87, p<0.001). There was a significant dose effect associated with the endurance performance (p=0.0191); moreover, the performance effects of ice slurry ingestion were not influenced by the timing of ingestion or environmental conditions. **CONCLUSION:** These data support the ingestion of ice slurry during endurance events in the heat. To optimize this simple cooling strategy in the field, it is recommended to ingest no more than 10 g⋅kg−1 before or during exercise.

### O52 Jumping performance of female volleyball players

#### Roberto Roklicer, Dusko Cvijovic, Suncica Pocek

##### Faculty of Sport and Physical Education, University of Novi Sad, Serbia

###### **Correspondence:** Roberto Roklicer (roklicer.r@gmail.com)

Vertical jump is a fundamental part of the spike, the block and serve. Jump is also used when setting, because it reduces the flight time of the ball, speeds up the attack and makes it harder for the first line of defense – block to read through possibilities of attacking team. **PURPOSE:** To examine whether there were differences in jumping performance between playing positions. **METHODS:** Thirty-four players were assessed for height, mass, spike reach, block reach and vertical jump performance – countermovement jump with arms swing (CMJa), spike (SJ) and block jump (BJ). MANOVA with LSD post hoc test was applied in order to examine whether there were statistically significant differences between player positions of setter (175.43±5.50, 59.91±10.43), opposite (178.25±3.20, 66.15±5.14), middle blocker (181.67±4.59, 65.16±9.58), wing spiker (172.25±5.61, 62.35±13.72) and libero (171.60±1.52, 58.44±2.44) in vertical jumping performance. **RESULTS:** There were no statistically significant differences (F=1.38; p=0.15; partη^2^=0.21). **CONCLUSION:** Taking into account the importance of VJ in the game of volleyball it is reasonable to expect similar results between positions in relative values. However, in the absolute values expected differences of the vertical jump should be reflected by the best results of middle blockers and opposites, followed by wing spikers, setters, and liberos as a consequence of demands of the game and body height value of players chosen through selection. Given results could be explained by the subject sample of nonelite players.

### O53 Daily physical activity and low screen time associated with positive well-being among young adolescents in Kazakhstan

#### Kwok Ng^1,2^, Alina Cosma^3^, Shynar Abdrakhmanova^4^, Assel Adayeva^4^

##### ^1^Department of Educational Sciences and Psychology, University of Eastern Finland, Finland; ^2^Department of Physical Education and Sport Sciences, University of Limerick, Ireland; ^3^Department of Interdisciplinary Social Science, Utrecht University, Netherlands; ^4^National Center of Public Health, Almaty, Kazakhstan

###### **Correspondence:** Kwok Ng (kwok.ng@hbsc.org)

**PURPOSE**: To investigate the associations between daily physical activity (PA) and screen time behaviors (STB) with well-being among a nationally representative sample of young adolescents in Kazakhstan. **METHODS**: Young adolescents (n=4153, 49.3% girls, Mean _age_=12.93 SD_age_=1.64) across Kazakhstan formed a national representative sample for the 2017/18 Health Behavior in School-aged Children (HBSC) study. Survey items included self-reported moderate-to-vigorous PA (MVPA) levels in the past week and the number of STB hours viewing during weekdays. The independent variables were the WHO-5 Wellbeing Index and Somatic complaint checklist. **RESULTS**: Young adolescents who engaged with one hour of MVPA every day (OR=2.83, CI=2.4-3.4) and spent less than three hours of STB during weekdays (OR 1.2, CI=1.0-1.4) were more likely to report positive well-being when compared with not daily PA and three or more hours of STB, respectively. Furthermore, those who reported daily MVPA (OR=0.7, CI=0.6-0.8) and low levels of STB (OR=0.7, CI=0.6-0.8) were less likely to report weekly somatic complaints. **CONCLUSIONS**: The results from this study confirm previous studies that report associations between mental health and meeting recommendations for PA and STB.

## Poster presentations

### P1 Sensory stimulation during 45 minutes of bed rest experiments

#### Cecil Meulenberg, Sasa Bele, Masa Somen, Uros Marusic, Bostjan Simunic

##### Institute for Kinesiology Research, Science and Research Centre Koper, Slovenia

###### **Correspondence:** Bostjan Simunic (bostjan.simunic@zrs-kp.si)

An occupational day comes with exposure to stress, either mild or strong, and in various forms of both physical and/or psychologic character. Next, to that, modern life creates many habits that require attention late in the evening. **PURPOSE**: Often during preparatory times before partaking in bedtime, the accumulated stress and the corresponding physiological responses e.g., enhanced brain activity and diminished fitness, hinder or prevent people from falling asleep easily. Enriching the sleeping environment with incorporated devices for sensory stimulation to influence sleep and/or waking, is gaining attention. **METHODS**: The current study investigates whether audio therapy, chromotherapy, aromatherapy, or a combination are effective in influencing the arousal state of a person, both during relaxation and awakening phases, comparable to periods occurring before and after sleep, respectively. **RESULTS**: From the physiological recordings, the descriptive subjective experiences by the subjects, and the questionnaires, it is clear that compared to baseline all conditions induced less anxious post-experimental emotional states. **CONCLUSION**: The sensory stimulations are not all equally effective in inducing relaxation.

### P2 Analysis of endurance capability in 11-14-year-old boys and girls

#### Nikolett Schulteisz^1^, Judit Gangl^1^, Ferenc Ihasz^1^, Josip Lepes^2^, Szabolcs Halasi^2^, Robert Papp^2^

##### ^1^Faculty of Health Sciences, University of Pécs, Hungary; ^2^Hungarian Language Teacher Training Faculty in Subotica, University of Novi Sad, Serbia

###### **Correspondence:** Nikolett Schulteisz (nikolett.schulteisz@gmail.com)

Growth, maturation, and performance are unified processes. **PURPOSE**: The aim of the research was to characterize the physical development of elementary school girls and boys(11-14years), their body composition, the differences between the age-dependent averages of cardiorespiratory performance and their pattern. **METHODS**: Examinations in 3 elementary schools (147 boys, 163 girls) in Győr. The anthropometric, body composition and endurance characteristics in children were calculated with two-sample T-test, while the sections crossed in 20m shuttle run test. The difference among and between age groups, genders, and fat content categories and the differences in body height averages based on fat content categories were calculated with Repeated ANOVA, Post hoc. Tuckey HDS method, p<0,05. **RESULTS**: Among the examined characteristics of 11-12year-old children, we found a significant difference between the values for the different sections, in favor of girls. In the group of 12-13year-olds, a significant difference in relative fat, relative muscle was found, at the 13-14year-olds, with the exception of the shuttle run test. Significant differences were found between the average results per sections achieved by boys, between 11-13 and 11-14year-olds. **CONCLUSION**: The coordination of the highly complex processes mentioned above, can be facilitated to a large degree by the individually planned and regularly performed physical activity.

### P3 Toward recognition of aggressive behavior and social exclusion among children and youth

#### Patrik Drid, Tatjana Trivic, Dragan Marinkovic, Ivana Milovanovic

##### Faculty of Sport and Physical Education, University of Novi Sad, Serbia

###### **Correspondence:** Patrik Drid (patrikdrid@gmail.com)

The paper represents results of field research conducted with children aged 10 – 14, who attend primary school and children active in sports, in Novi Sad. **PURPOSE**: To determine the frequency of aggressive behavior and social exclusion through questionnaires and observation amongst youth. To determine the frequency of personal experience of peer violence or social exclusion of the subjects and to determine correlations between measures of aggressive behavior and social exclusion. **METHODS**: The survey covered 118 respondents, 41 athletes, and 77 students. The average age in both groups of participants is 12.3 years. **RESULTS**: The frequency of aggressive behavior is 32% in the sample (38/118), with no significant gender differences: 34.5% of boys and 31% of girls. **CONCLUSION**: General satisfaction with the climate in class is in a positive correlation with intrinsic motivation, self-assessment, optimism, and hope: those students who are intrinsically motivated to engage in sports are more satisfied with their class, but they also assess themselves with high scores on self-assessment, hope and optimism scales. Also, there are significant differences in the manifestation of aggressive behavior among students and athletes.

**ACKNOWLEDGMENTS**

Supported by the Serbian Ministry of Education, Science and Technological Development (179011), Provincial Secretariat for Higher Education and Scientific Research (142-451-2473) and Faculty of Sport and Physical Education.

### P4 Somatotypes of elite male and female cadet sambo athletes

#### Tatjana Trivic^1^, Sergey Tabakov^2^, Damjan Jakisic^1^, Zeljko Krneta^1^, Valdemar Stajer^1^, Milorad Bejatovic^3^, Patrik Drid^1^

##### ^1^Faculty of Sport and Physical Education, University of Novi Sad, Serbia; ^2^Russian State University of Physical Education, Sports and Tourism, Moscow, Russia; ^3^University Business Academy in Novi Sad, Faculty of Law, Serbia

###### **Correspondence:** Tatjana Trivic (ttrivic@yahoo.com)

During the International Olympic Committee’s Executive Board meeting held in Tokyo, Japan on 30 November 2018, provisional recognition was granted to the International SAMBO Federation (FIAS) pursuant to Rule 25 of the Olympic Charter. **PURPOSE**: The aim of the present study was to determine somatotype and anthropometric profiles of elite cadet sambo athletes divided by weight categories. **METHODS**: A total of 96 elite cadet sambo athletes from 28 countries, participants of the World Cadet Sambo Championships 2018 participated in the study (40 females and 56 males from 10 weight categories). Anthropometrical variables were taken in order to calculate somatotypes. **RESULTS:** A typical somatotype in male sambo athletes was endomorphic mesomorph with indicating a predominance of musculoskeletal tissue, while female athletes were classified as endomorphic mesomorph in relation with weight division. **CONCLUSION:** Future research should attend to these differences by category to generate appropriated conclusions about the role of anthropometric characteristics in sambo performance. It is necessary to reduce the number of weight categories.

**ACKNOWLEDGMENTS**

Supported by the Serbian Ministry of Education, Science and Technological Development (179011), Provincial Secretariat for Higher Education and Scientific Research (142-451-2473) and Faculty of Sport and Physical Education.

### P5 Physical activity mediates the association between perceived stress and subjective well-being among university students

#### Valerii Olefir^1^, Viktor Plokhikh^2^, Valerii Bosniuk^3^

##### ^1^Department of General Psychology, Faculty of Psychology, V. N. Karazin Kharkiv National University, Ukraine; ^2^Department of Developmental Psychology and Social Communications, South Ukrainian National Pedagogical University, Odesa, Ukraine; ^3^Department of Psychology of Activities in Special Conditions, National University of Civil Defense of Ukraine, Kharkov, Ukraine

###### **Correspondence:** Valerii Olefir (vaolefir@gmail.com)

Physical activity improves subjective well-being; however, how physical activity mediates perceived stress has not been studied. **PURPOSE**: The present study investigated whether physical activity buffers the association between perceived stress and subjective well-being. **METHODS**: A sample of 300 university students (65.3% women) with a mean age of 19.63 ± 2.13 years participated in this study. The questionnaires consisted of socio-demographic characteristics, The Satisfaction with Life Scale (5 items), Subjective Happiness Scale (4 items) and Perceived Stress Scale (10 items). In addition, physical activity was measured, which was based on regular exercises in the sports sections. Data were analyzed using Mplus 7.2. **RESULTS**: The study revealed that students who were more physically active, compared to those who were less active, were less susceptible to stress and experienced higher levels of subjective well-being. The findings also indicated that physical activity buffers the association between perceived stress and subjective well-being (indirect effect = -0.029, SE =0.014, p=0.035) **CONCLUSION**: This study concluded that a physically active lifestyle could mediate the negative effects of stress and improve the level of subjective well-being.

### P6 The effects of the comprehensive rehabilitation method on strength outcomes in ACL patients

#### Irina Roy^1^, Andrey Rusanov^1^, Lyudmila Kravchuk^2^, Оlga Rusanovа^2^

##### ^1^Institute of Traumatology and Orthopedics, National Academy of Medical Sciences, Kiev, Ukraine; ^2^National University of Physical Education and Sports of Ukraine, Kiev, Ukraine

###### **Correspondence:** Andrey Rusanov (rusanova2080@gmail.com)

**PURPOSE**: The aim of this study was to determine the effectiveness of a novel rehabilitation protocol on quadriceps muscle strength in ACL patients. Experimental protocol consisted of stabilographic platform workout, remedial gymnastics and therapeutic massage aimed to increase knee range of motion and post-isometric relaxation as well as knee mechanotherapy with workout machines. **METHODS**: All patients (52) underwent the rehabilitation treatment in the rehabilitation department of ITO NAMS of Ukraine and were operated in the hospitals of the institute. According to the proposed program, 21 patients (experimental group) underwent a novel rehabilitation protocol after ACL reconstruction while 31 patients received the traditional program of physical rehabilitation. **RESULTS**: In the early postoperative period (from the 3rd to the 16th week after surgery), the average statistic of muscle strength of the affected limb in experimental group was significantly (p <0.05) higher than the control group's patients/ In the control group, according to the dynamometry, there was a significant decrease in the injured vs non-injured leg strength (p <0,05). When performing reduction - on 25,02%, withdrawal - by 26,45%, bending in the knee joint - by 58,64% and extension - by 67,63%. **CONCLUSION**: The use of the experimental program increased the effectiveness of ACL-patients rehabilitation.

### P7 Differences in parameters of functional and motor capacities in football players of different age category and position

#### Ivan Mikulic, Matko Galic, Stipo Dajakovic, Jere Gulin

##### Faculty of Kinesiology, University of Zagreb, Croatia

###### **Correspondence:** Stipo Dajakovic (stipo.dajakovic@kif.hr)

Football is a highly complex activity where an important component of the developmental path of every football player are technical-tactical and strength and conditioning coaches. Their role is reaching every player's potentials by using various training and selection methods. PURPOSE: The aim of the study was to determine differences in parameters of functional and motor capacities between football players of different age categories and positions. METHODS: Fifty-one lower league Football Club "HAŠK" player (age: 11 to 32) was divided in groups according to age category: young pioneers (n=12), older pioneers (n=17), cadets (n=10), juniors (n=6), seniors (n=6); and playing position: goalkeeper (n=6), central defender (n=11), full-back (n=9), midfielder (n=14), forward (n=11). Participants performed standing long jump test, Sargent test, sprint 20m, 9-3-6-3-9 sprint test, Ajax test 5x10m, and beep test. RESULTS: Univariate analysis of variance (ANOVA) for independent samples revealed statistically significant differences in all motor and functional ability tests (standing long jump test, Sargent test, sprint 20m, 9-3-6-3-9 sprint test, Ajax test 5x10m, p=0,000; and beep test, p=0,002), between players of different age categories. CONCLUSION: We conclude that appropriate assessment of football-specific motor abilities provides valuable information for coaches on player's fitness and allows a higher quality of training planning and programming, as well as proper development of the muscular-skeletal system of younger age groups.

### P8 Exercise for cognitive health as a proxy for the social inclusion of older people: a systematic review

#### Stevo Popovic, Dusko Bjelica, Bojan Masanovic, Jovan Gardasevic

##### Faculty for Sport and Physical Education, University of Montenegro, Montenegro

###### **Correspondence:** Stevo Popovic (stevop@ucg.ac.me)

Some previous studies suggest social isolation is associated with poor cognitive health in later life, while cognitive outcomes may increase the social inclusion of older people and cognitive health may be an important aspect of healthy aging. **PURPOSE**: The goal of this study is to review the available literature on research related to the relationship of exercise for cognitive health and social inclusion of the elderly. **METHODS**: Specific keywords "exercise", "cognitive", "health", "social", "inclusion", "elderly", and "people" were used to search relevant electronic databases, such as PubMed, Web of Science and Scopus. The research was conducted according to PRISMA guidelines. **RESULTS**: Studies that fit the inclusion criteria such as containing the data with the publication time that was from 2008 and later, and describing various exercise related to an improvement of cognitive health, were reviewed. Results have shown that beneficial effects of exercise on cognitive health of older people have been observed. The dose of exercise is mostly recommended to be on the level of an hour a day, and three times a week, while the most applicable are aerobic, strength training exercises and yoga, and its combination, all in order to increase cognitive health. **CONCLUSION**: The findings suggest that various exercise may benefit global-cognition, attention and cognitive control, as well as improve the social inclusion of older people.

### P9 Impact of professional sports engagement on the extension of life: a systematic review

#### Dusko Bjelica, Stevo Popovic, Bojan Masanovic, Jovan Gardasevic

##### Faculty for Sport and Physical Education, University of Montenegro, Montenegro

###### **Correspondence:** Dusko Bjelica (sportmont@t-com.me)

**PURPOSE**: The present study aims to review the available literature on research related to professional sports engagement on the extension of life to summarize the potential risk factors associated with longevity and trends and causes of mortality among professional sportsmen. **METHODS**: Specific keywords "professional", "sport", "sportsmen", "athletes", "death", "longevity", and "mortality" were used to search relevant electronic databases, such as PubMed, Web of Science and Scopus, as well as some international journals and proceedings of Public Health and Medicine in Sport. The research was conducted according to PRISMA (Preferred Reporting Items for Systematic reviews and Meta-analyses) guidelines. **RESULTS**: Studies that fit all inclusion criteria such as containing the data with the publication time that was between 2000 and 2018, subjects that were professional sportsmen and the goals that were related to longevity and mortality were reviewed. Results have shown that some team sports (football and basketball) and individual sport (cycling), as well as professional sportsmen from mixed and sports with endurance, had strong relationships with longevity. **CONCLUSION**: Professional sports engagement is favorable to lifespan longevity, while the potential risk factors associated with mortality are not securely recognized and sports achievement, playing position and handedness may affect it.

### P10 Upper respiratory tract infections in water sports elite athletes in Uzbekistan

#### Fikrat Kerimov^1^, Jamshid Umarov^1^, Abdurakhim Toychiev^2^, Svetlana Osipova^2^

##### ^1^Faculty of All-Around Sports, Uzbek State University of Physical Education and Sport, Chirchik, Uzbekistan; ^2^Research Institute of Epidemiology, Microbiology and Infectious Diseases, Tashkent, Uzbekistan

###### **Correspondence:** Svetlana Osipova (svetosip7@mail.ru)

Upper respiratory tract infections (URTI) are common diseases in athletes. Water sports are characterized by specific features that can increase susceptibility to URTI. **PURPOSE**: To detect the prevalence of URTI in swimmers and athletes engaged in synchronized swimming as well as serum 25(OH)-vitamin D and total serum IgE level. **METHODS**: Study participants included 20 elite athletes engaged in synchronized swimming and 20 elite female swimmers at the age of 19-24 years. Control group included 30 healthy individuals of the same sex and age. Morbidity, frequency, and duration of URTI were determined by self-reported questionnaire and medical history. Total serum IgE (HUMAN kit, Germany) and serum level of 25(OH)-vitamin D (DIAsource kit Belgium) were determined by ELISA. **RESULTS**: Prevalence of URTI morbidity and vitamin D deficiency/insufficiency depends on the season but in every season the highest values were observed in athletes (P<0.05). Both groups of athletes were characterized by an elevated level of total serum IgE, more expressed in synchronized swimmers (P<0.05). **CONCLUSION**: Increase of URTI morbidity in athletes seems to depend on vitamin D deficiency/insufficiency and elevated IgE concentration. Monitoring and correction of vitamin D level are advisable. Elevated IgE concentration can be mediated by exposure to chronic low dose of chlorine and other environmental allergens.

### P11 The difference in motives for exercise participation between men and women

#### Damir Pekas^1^, Mario Baic^1^, Miroslav Zecic^1^, Nebojsa Trajkovic^2^

##### ^1^Faculty of Kinesiology, University in Zagreb, Croatia; ^2^Faculty of Sport and Physical Education, University of Novi Sad, Serbia

###### **Correspondence:** Damir Pekas (damir.pekas@kif.hr)

One of the key issues in physical activity research is the determination of motives for exercise participation man and woman. **PURPOSE**: The aim of this research was to determine the gender differences in motives for exercise participation in men and women. **METHODS**: The participants were 138 men and 80 women involved in organized physical activities through different fitness training centers in Zagreb. Campbell’s Questionnaire with 13 items for motivation was used in order to determine the motives for exercising in people aged 18 to 76 years. Differences in the motivation of men and women were tested by the Mann-Whitney test. **RESULTS**: Statistically significant gender differences were found in four factors of motivation: to look good (p=0.02), to relax (p=0.01), to control or lose weight (p=0.01) and to seek adventure and excitement (p=0.02). Additionally, men and women differ according to education, job and their willingness to pay for exercising. **CONCLUSION**: Men and women show differences in motives for exercise, especially concerning the exercise organized in fitness training centers.

### P12 Genders differences in the level of motor coordination of students of prepubertal age

#### Zivan Milosevic, Maja Batez, Marijana Simic, Filip Sadri

##### Faculty of Sport and Physical Education, University of Novi Sad, Serbia

###### **Correspondence:** Zivan Milosevic (zivanmilosevic991@gmail.com)

Sedentary behavior patterns of children negatively reflect on their motor coordination and a low level of motor coordination can negatively affect student participation in physical activities. **PURPOSE**: This study was conducted to evaluate the differences in the level of motor coordination between genders. **METHODS**: The sample of respondents consisted of 243 students (boys 114, girls 129, aged 9-10). Motor coordination was measured with a body coordination battery test (KTK - Körperkoordinations Test für Kinder) which consists of four motor tasks who testing balance, rhythm, lateral movement, speed, and agility. A Mann-Whitney test analysis was used to determine the differences in the degree of motor coordination. **RESULTS**: The results showed that there are statistically significant differences in total motor coordination (p =0.02), as well in the test hoping for height (p=0.04) in the benefit of boys. **CONCLUSION**: In general, better results of boys in the gross motor coordination can be explained by the influence of biological and environmental factors. It can be assumed that gender differences are conditioned by different interests in physical activity, where boys are more likely to determine for sports such as football and basketball in which coordination is developed, while girls are more likely to determine for sports such as gymnastics and ballet in which most develops flexibility.

### P13 Social support in relation to quality of life of children

#### Nebojsa Maksimovic, Zoran Milosevic, Ivana Milovanovic, Radenko Matic, Damjan Jaksic, Dusan Corilic

##### Faculty of Sport and Physical Education, University of Novi Sad, Serbia

###### **Correspondence:** Radenko Matic (radenkomatic@uns.ac.rs)

Social support affects the quality of life in children. **PURPOSE:** Research aim was to examine the differences between social support to children and some characteristics of the quality of life of children. **METHODS:** Study enclosed children aged 11-12 years (N=933), 468 boys and 465 girls. The assessment of social support included the attitudes of children with support from parents, classmates, teachers and closest friends using the SSSC questionnaire. The quality of life of children is determined by gender, living place, SES, school success, level of physical activity (using the International Physical Activity Questionnaire). **RESULTS:** The methods of multivariate and univariate statistics were used to examine the significance of differences, which were found in factors: gender, school success and physical activity on level p<0.01, (F=3659.39; P=0.00). In all dimensions, girls had a better level than boys. In terms of school success, the results showed that children with low and excellent success have a higher level in all dimensions of social support than children with intermediate success. Results showed that high-level children' physical activity is followed by social support of parents. Differences in living place and SES haven't been confirmed. **CONCLUSION:** Research showed that social support by parents and gender are factors which determinate the quality of life of children.

### P14 Age-related differences in motor coordination and balance skills of young school-age children

#### Adriana Ljubojevic, Kenan Omicevic, Snezana Bijelic, Tamara Karalic

##### Faculty of Physical Education and Sport, University of Banja Luka, Bosnia and Herzegovina

###### **Correspondence:** Adriana Ljubojevic (adriana.ljubojevic@ffvs.unibl.org)

A professionally guided development of motor skills, most often, begins starting at school, within physical education classes. PE teachers of young school-age children have to synchronize motor tasks with the developmental characteristics and needs of a specific age. **PURPOSE**: To determine age-related differences in motor coordination and balance skills of young school-age children. **METHODS**: The sample of respondents consisted of 60 subjects aged 7 and 9 years old (30 seven-year-old pupils and 30 nine-year-old pupils) who attend Second Elementary School in Zivinice in Bosnia and Herzegovina. The motor coordination was assessed by standardized tests: obstacle course backward, the "figure eight" with bending, crawling through and jumping over, side steps, zigzag run with two balls and dribbling. Tests that measure balance were: standing on two legs on a balance beam, across, eyes open; standing on two legs on a balance beam, along, eyes open; walking forward on a low beam and flamingo balance test. **RESULTS**: Analysis of T-tests for independent samples show statistically significant differences in all coordination tests between seven-year-olds and nine-year-olds, while no significant age-related differences were detected in balance tests. **CONCLUSION**: The results are important in terms of planning and programming age-appropriate movement games for physical education classes.

### P15 Physical fitness differences among different competitive-level basketball players

#### Bojan Masanovic^1^, Zoran Milosevic^2^, Marin Corluka^3^, Velisa Vukasevic^4^

##### ^1^Faculty for Sport and Physical Education, University of Montenegro, Montenegro; ^2^Faculty of Sport and Physical Education, University of Novi Sad, Serbia; ^3^Faculty of Mathematics and Science Education, University of Mostar, Bosnia and Herzegovina; ^4^Basketball Club Vizura, Belgrade, Serbia

###### **Correspondence:** Bojan Masanovic (bojanma@ucg.ac.me)

Some previous studies suggest that motor ability have an impact on the performance of playing tasks. **PURPOSE**: This study examined the physical differences in adult male basketball players of different competitive level. **METHODS**: Forty-eight males were enrolled in the study, divided into two groups: twenty-four senior players from the Super League and twenty-four senior players from the Second Basketball League of Serbia. Standardized tests for the assessment of motor abilities were used. The differences were determined by a t-test for small independent samples. **RESULTS**: The results showed that a significant difference in favor players from a higher competitive level was found for six tests among the group: vertical jump (Sargent), standing triple jump, sit-ups for 30 seconds, running 4x5 meters, T-test and 20 suicides in 20 minutes, while the difference was not observed for three tests: 20m high start running, distance ball throwing and push-ups. **CONCLUSION**: Basketball players physical fitness can differentiate between competitive level. These findings may give coaches better working knowledge about the characteristics of top basketball players, and suggest them on which variables to focus their attention during training and in the process of talent identification.

### P16 Nine months of developmental gymnastics program improves locomotor skills in pre-school children

#### Danilo Radanovic, Nebojsa Trajkovic, Boris Popovic, Dejan Madic

##### Faculty of Sport and Physical Education, University of Novi Sad, Serbia

###### **Correspondence:** Danilo Radanovic (radanilo17@yahoo.com)

Fundamental motor skills are the foundation for more complex and specialized motor skills. That is why developing these skills in early childhood is so important. **PURPOSE**: The aim of this research was to examine the effects of developmental gymnastics program in pre-school children. **METHODS**: Sample consisted of 112 preschool children (66 boys; age 4.42 ± 0.38 years). There were 38 children (28 boys; age 4.57 ± 0.37 years) in the experimental group, which was involved in the programmed exercise of developmental gymnastics in a sports school. Training was two times a week in a duration of one hour. Control group consisted of 74 children (38 boys; age 4.34 ± 0.37 years) from the Preschool Institution „Radosno detinjstvo“, Novi Sad, Serbia. Only healthy children with signed parent approval were tested. Three locomotor skills (run, hop and horizontal jump) were assessed using Test of Gross Motor Development, second edition (TGMD-2). **RESULTS**: The results implicate statistically significant larger improvement (p < 0.05) of the experimental group in all three locomotor skills (run, hop and horizontal jump for 4.7 %, 19.7 % and 39.7 %, respectively) compared to control group. **CONCLUSION**: The experimental treatment used during the period of 9 months enabled children of the gymnastics group to reach higher levels of all three mentioned skills.

### P17 Effects of treatment on the endurance trunk in men with postural imbalance

#### Tijana Scepanovic, Dejan Madic, Branka Protic-Gava

##### Faculty of Sport and Physical Education, University of Novi Sad, Serbia

###### **Correspondence:** Tijana Scepanovic (tijanascepanovic021@gmail.com)

Core treatment improves endurance trunk in people with Chronic low back pain but no research was performed on postural imbalances. **PURPOSE**: The objective of the present study is to evaluate the effect of 6-week core treatment on trunk endurance performance men with postural imbalances. **METHODS**: This study is quasi-experimental study in which 138 male subjects with sagittal plane postural disorders at the age 20±0.5 years ([experimental group: n=73; BMI=23.27±2.48] [control group: n=65; BMI=23.91±2.35]). The experimental group received a lumbar core stabilization training for six weeks. The study of endurance trunk was evaluated in accordance with various clinical tests such as Back extensor test - Sorensen (BET), Abdominal flexor endurance test (FET) and Double leg lowering test (DLL). **RESULTS**: Positive effects were obtained after the application of the core treatment in favor of the experimental group in all three analyzed tests (p = 0.00). On initial testing in the FET and DLL test, the control group had better results. On final testing, the experimental group has statistically better results in the BET and FET test. **CONCLUSION**: This study concludes that it is possible to improve in the lower trunk endurance performance and affect postural imbalances after 6-weeks.

### P18 Test-retest reliability of change of direction speed tests in adolescent volleyball players

#### Milos Ignjatovic^1^, Vladan Milic^2^, Nebojsa Trajkovic^3^

##### ^1^Elementary school “Kralj Aleksandar I”, Gornji Milanovac, Serbia; ^2^Department of Sport and Physical Education, State University of Novi Pazar, Serbia; ^3^Faculty of Sport and Physical Education, University of Novi Sad, Serbia

###### **Correspondence:** Milos Ignjatovic (milosskomi@yahoo.com)

Volleyball is an intermittent sport in which players are involved in defensive and offensive activities where power, strength, agility, speed, and change of direction speed (COD) are required. **PURPOSE:** The aim of this paper was to determine the test-retest reliability of COD speed tests in adolescent volleyball players. **METHODS:** Twenty-eight adolescent volleyball players (15+0.6 years) gave their consent to be part of this study. For this purpose, the subjects participating in the study took different COD tests: sprint with 90 turns, sprint 9-3-6-3-9 m with 180 turns, and sprint 9-3-6-3-9 m with backward and forward running (SBF). Systematic bias was investigated using a t-test and the relative reliability was determined by calculating ICC. **RESULTS:** There were no significant differences (p>0.05) between tests and retest for all tests. The test-retest methods reliability coefficients varied between 0.89 and 0.97. Of all the COD speed tests, the SBF had the greatest ICC (0.976). The within-subject variation (CV%) ranged between 2.4 and 4.6%. **CONCLUSION:** It can be concluded that of all COD speed tests used in this study, the SBF was the most reliable test for estimating the COD speed in adolescent volleyball players.

### P19 Electrical myostimulation in patients with quadriceps muscle hypotrophy after an arthroscopic restoration of ACL

#### Lyudmila Kravchuk^1^, Zinchenko Vitaly^2^, Andrey Rusanov^2^, Olga Rusanova^1^

##### ^1^National University of Physical Education and Sports of Ukraine, Kiev, Ukraine; ^2^Institute of Traumatology and Orthopedics of the National Academy of Medical Sciences, Kiev, Ukraine

###### **Correspondence:** Lyudmila Kravchuk (kravchukwww@gmail.com)

One of the frequent complications after arthroscopic anterior cruciate ligament (ACL) repair is a hypotrophy of quadriceps muscle (QM). **PURPOSE**: To assess the effectiveness of electromyostimulation on the restoration of the QM after ACL arthroscopy. **METHODS**: 60 patients in the early postoperative period (3-4 weeks) were examined, and were divided into 2 groups: control group (CG, n = 30) and main group (MG, n = 30). CG took a course of remedial exercises to restore the strength QM, while MG underwent a course of electromyostimulation procedures for m. rectus femoris. Efficiency was assessed using DIGITAL M-TEST computer electroneuromyography (EMG). **RESULTS**: Before rehabilitation EMG average amplitude for m. rectus femoris of the injured limb showed unreliable differences between groups (MG 87.5 ± 6 mcV vs. CG 89.2 ± 7 mcV; p > 0.05). EMG amplitude for m. rectus femoris at 14-day follow-up slightly increased in CG (94.5 ± 6 mcV), while values showed a marked increase in MG (126.5 ± 8 mcV), (p ≤ 0.05). **CONCLUSION**: Recovery of the bioelectrical activity of muscles after the electromyostimulation is more effective than after using the physical exercise.

### P20 Differences in motor abilities of children between 7 and 9 years of age according to spinal deformities

#### Branka Protic-Gava, Milan Kojic, Zoran Milosevic, Nebojsa Maksimovic, Tijana Scepanovic

##### Faculty of Sport and Physical Education, University of Novi Sad, Serbia

###### **Correspondence:** Branka Protic-Gava (brankapg@gmail.com)

The lack of reports in the available literature on the quality of spine posture and relation to the motor abilities among children in early childhood prompted us to investigate this. **PURPOSE**: To analyze differences in motor abilities of children with various spinal deformities. **METHODS**: A sample of 79 both gender children participated in this cross-sectional study (50 males 8.09±0.62; 29 females 7.91±0.63 decimal years). On the basis of the posture index, the total sample is divided into three groups (good, poor and very bad spine posture). All motor ability tests were performed in accordance with the EUROFIT battery of tests for physical readiness. The spine posture was assessed based on the evaluation of 2D Posture Analysis by Fröner (Contemplas). Spinal posture differences examined using the Kruskal Wallis Test, p < 0.05. **RESULTS**: There are statistically significant differences in motor abilities among children with different sagittal plane deformities. Children with good posture had better results: obstacle and slalom course [χ² =7.005, df=2, ρ=0.030]; plate tapping test (χ²=9.534, df=2, ρ=0.009); standing horizontal jump [χ² =5.978, df=2, ρ=0.050]; sit-and-reach (χ² =7.276, df=2, ρ=0.026); 20-m dash (χ²=9.127, df=2, ρ=0.010). No significant differences were detected in the coronal plane. **CONCLUSION**: Children with good spine posture displayed better motor abilities than their peers.

### P21 Comparison of two metabolic risk scores in obese adolescents – whether the inclusion of cardiorespiratory fitness matters? A pilot study

#### Lavinia La Grasta Sabolic^1^, Maja Cigrovski Berkovic^2^, Borislav Valjan^1^, Marija Pozgaj Sepec^1^, Gordana Stipancic^1^

##### ^1^Department of Pediatrics, Sestre Milosrdnice University Hospital Center, Zagreb, Croatia; ^2^Department of Internal Medicine, Sestre Milosrdnice University Hospital Center, Zagreb, Croatia

###### **Correspondence:** Lavinia La Grasta Sabolic (lavinia.la.grasta@zg.t-com.hr)

Various approaches are used for calculation of cardiometabolic risk in youth, including continuous scores (cMS), which are commonly expressed as sum of age and sex-specific z-scores for waist circumference (WC), triglycerides (TG), high-density cholesterol (HDL), blood glucose (BG) and systolic blood pressure (SBP). Recently, a novel, easier to calculate continuous pediatric risk score (PsiMS) has been developed. Purpose: To investigate the correlation between PsiMS and cMS scores and to explore whether the inclusion of cardiorespiratory fitness (CRF) alters the score. Methods: Data on 30 obese adolescents (BMI ≥95th percentile, mean age 13.7 years, 15 female/15 male) were retrospectively analyzed. PsiMS and cMS scores were calculated using the same set of variables (WC, TG, HDL, BG, SBP), with or without CRF included. Results: PsiMS score calculated as (2xWC/height) + (BG (mmol/l)/5.6) + (TG (mmol/l)/1.7) + (SBP (mmHg)/130) - (HDL (mmol/l)/1.02) correlated well with cMS score (Spearman's rank order correlation coefficient 0.80, p<0.01). A very high correlation was found between PsiMS scores calculated with/without CRF (0.99, p<0.01). Conclusion: PsiMS score represents a practical and accurate score for evaluation of cardiometabolic risk in obese youth. The inclusion of CRF in PsiMS score does not change the risk score.

### P22 Study regarding the flow state in yoga and Pilates

#### Simona Petracovschi (simona.petracovschi@e-uvt.ro)

##### Faculty of Physical Education and Sport, West University of Timisoara, Romania

.

### P23 Optimization of movement activity and the mental state of students by dance aerobics

#### Natalia Chuprun, Sergey Zakopaylo, Mykola Shulga, Alexander Gordienko

##### Pereiaslav-Khmelnytskyi Hryhorii Skovoroda State Pedagogical University, Pereiaslav-Khmelnytskyi, Ukraine

###### **Correspondence:** Natalia Chuprun (chuprunn@ukr.net)

**PURPOSE**: To test the effectiveness of dance aerobics to optimize the motor activity and the psychophysical state of female students. **METHODS**: Students of the I-II courses have taken part in the experiment of the pedagogical faculty with a total of 60 subjects. The number of training hours for dance aerobics was 64 hours. Medical and biological: body weight (kg), BPM and BPD (mmHg), ChSS in a state of rest and the Stange test. All the data obtained by the study were processed by the procedures of descriptive and comparative statistical methods. **RESULTS**: During the studying, and especially the examination time, students experience strong psycho-emotional stress and the physical state becomes worse. Comparative analysis of data confirmed the effectiveness of dance aerobics tools not only in the absence of negative changes during the examination session, but also improvement of the psychophysical state (state of health by the method of SHAN in kg - 3,8 points, EG1 - 4,3 points, EG2 - 4,5 points, ЕG3 - 4.8 points) and the level of somatic health of students (in Kg 0.23 ± 0.04 points, ЕG1 8.78 ± 0.50 points, ЕG2 8.77 ± 0.61 points, ЕG3 11, 65 ± 0.55 points). **CONCLUSIONS**: The use of dance aerobics has a positive effect on the psychophysical state of female students and the optimization of their physical activity.

### P24 The effects of a semi-annual physical exercise program on the fat tissue of pre-school children

#### Zeljko Krneta, Filip Sadri

##### Faculty of Sport and Physical Education, University of Novi Sad, Serbia

###### **Correspondence:** Zeljko Krneta (krnetafsfv@gmail.com)

**PURPOSE**: The aim of the research was to determine whether organized physical exercise can influence the reduction of fat tissue in pre-school children. **METHODS**: The research involved 201 pre-school age children. The experimental group consisted of 109 children (5.76±81) who had additional physical activity over a period of 6 months, 2 times a week for 60 minutes. The control group consisted of 92 children (5.62±.57) who did not have additional physical activity. Data was collected for height, waist circumference, subcutaneous fat tissue on the forearm, abdomen, and back. From the above measures, Waist to Body Height Ratio (WtHR) and Sum of Subcutaneous Fat Measures (SFM) was calculated. The t-test for dependent samples was used to test the difference between the average measurement values. **RESULTS**: A statistically significant reduction in the average values of WtHR (p=.005; Mdf=.006) and SFM (p=.002; Mdf=12.62) was determined in the experimental group. The control group showed a statistically significant increase in the mean of SFM (t=-2.14; p=.035; Mdf=-12.12), while changes in the WtHR were not statistically significant. **CONCLUSION**: The results indicate that relatively short, organized, and professionally led additional physical activity can contribute to the reduction of the amount of subcutaneous and visceral fat in preschool children.

**Acknowledgments**

Supported by the Serbian Ministry of Education, Science and Technological Development (179011).

### P25 Dynamics of improving coordinating abilities in children 6–9 years

#### Leonid Volkov^1^, Volodymyr Volkov^2^, Natalia Chuprun^1^

##### ^1^Pereiaslav-Khmelnytskyi Hryhorii Skovoroda State Pedagogical University, Pereiaslav-Khmelnytskyi, Ukraine; ^2^National Pedagogical Dragomanov University, Kiev, Ukraine

###### **Correspondence:** Natalia Chuprun (chuprunn@ukr.net)

Explore the dynamics of development of coordination abilities in children 6-9 years. The experiment involved 278 elementary school students. Pedagogical testing: holding posture with open eyes (c); shuttle running 3x10m., s; running on the spot during 10s, 30s, 60s, (s); walking in the straight line without a visual guide, (sm); throwing rings on a tripod from a distance of 1.5m, 2.5m, 3.5m, (number of hits); game task "Transmit telegram", (one point); three moves forward with the exit (s); carpal dynamometry in full force and 50% of maximal power, (kg). Favorable periods were identified for the formation of the main components of coordination abilities in younger schoolchildren, namely: the differentiation of muscular efforts – in boys from 7 to 9 years old, in girls from 6 to 8 years old (P <0.05) sense of balance – in boys from 7 to 8, in girls from 6 to 9 years old (P <0.05) sense of rhythm – in boys from 6 to 7, in girls from 7 to 9 years old (P <0.05) accuracy of movements – in boys from 6 to 8, in girls from 8 to 9 years old (P <0.05) spatial orientation – in boys from 8 to 9 years old, in girls from 6 to 7 years old (P <0.05).

### P26 Trends and prevalence between body mass index and physical activity among students in Novi Sad

#### Dusica Rakic^1^, Branislava Teofilovic^1^, Ljiljana Suvajdzic ^1^, Aleksandar Takaci^2^

##### ^1^Faculty of Medicine, University of Novi Sad, Serbia; ^2^Faculty of Technology, University of Novi Sad, Serbia

###### **Correspondence:** Dusica Rakic (dusica.rakic@mf.uns.ac.rs)

**PURPOSE**: The aim of this study is to determine the correlation between nutritional status and physical activity among students in Novi Sad. **METHODS**: This research has been conducted among students of the University of Novi Sad in 2013 and 2018. The study included 860 students in 2013 and 514 students in 2018 (aged between 19 and 24) and both genders were equally represented. For the assessment of nutritional status Body Mass Index - BMI was used. An original anonymous questionnaire was designed for this study. Statistical analysis was performed in SPSS20. **RESULTS:** A significant decrease was observed in the prevalence of overweight and obese students (from 21.8% to 14,4%) during the five years. In both studies, male students had a greater tendency to be overweight and obese and sex differences were statistically significant (p <0.001). In 2013 male students were more physically active compared to female students and sex differences were significant (p<0.001). BMI was positively correlated with a sedentary lifestyle (p<0.05). Overweight and obese students spent more than 1h in front of the monitors and statistical significance was p <0.001. **CONCLUSION** The prevention of overweight and obesity should be directed to increasing the level of physical activity and reducing the sedentary lifestyle.

### P27 Influence of the programmed annual cycle of exercises on the motor status of children aged 4 to 5

#### Maja Hecimovic^1^, Katarina Ohnjec^2^, Iva Kanjugovic^3^

##### ^1^“Sportska piramida”, Zagreb, Croatia; ^2^Faculty of Kinesiology University of Zagreb, Croatia; ^3^RK “Lokomotiva” Zagreb, Croatia

###### **Correspondence:** Katarina Ohnjec (katarina.ohnjec@kif.hr)

Physical fitness during childhood and adolescence is a powerful marker of future health. **PURPOSE**: Analyze the impact of a one-year training program on changes in the motor status of kindergarten children. Examine the differences between boys and girls in some motor skills at different periods of the program. **METHODS**: Ninety-six children, aged four to five (41 girls and 55 boys) participated in this research. Participants were tested in four variables: shuttle run test, handgrip strength, sit up and sit and reach. The measurement was carried out at three different points: initial, transitional and final. T-test for dependent samples and ANOVA were applied for the analysis of differences. **RESULTS**: Differences in the results of the initial and final measurement in all four variables were obtained. There are differences in the initial measurement of the agility test in boys and girls (F=4.00; p<0.05), and at all points of flexibility measurement (F1=13.76; F2=17.18; F3=19.16; p<0.01). In the second and third test, the difference was not obtained. **CONCLUSION**: The motor status of children was affected by the program throughout the training phases. Differences in the shuttle run test (agility, coordination, speed) between boys and girls were obtained in the beginning. The initial difference in flexibility remained also in the final measurement.

### P28 Do the stroke index and a number of breaths affect the swimming results in butterfly-technique?

#### Goran Dimitric, Nebojsa Cokorilo, Goran Vasic, Milorad Jaksic, Milica Blagojevic

##### Faculty of Sport and Physical Education, University of Novi Sad, Serbia

###### **Correspondence:** Goran Dimitric (dimitric@uns.ac.rs)

**PURPOSE**: The purpose of this study was to find out if stroke index and a number of breaths affect the swimming results on 50m and 100m butterfly stroke in short and long pool. **METHODS**: Study group consisted of 62 finalists, aged 19 ± 2 at the Serbian national championship on 50m and 100m, butterfly stroke on a short and long course. Swimming time was measured, whereas strokes and the number of breaths were counted. The collected data were processed using SPSS package; the descriptive statistics and linear regression results are showed. **RESULTS**: On this sample, linear regression showed that stroke index significantly affects swimming results on 50m and 100m butterfly stroke in short and long pools, but it didn't show significant effects of the number of breaths on the results on the same distances. **CONCLUSION**: Overall, these results showed that the stroke index on 50m and 100m butterfly significantly affects the swimming results, i.e. the bigger stroke index the better swimming result. This suggests that the swimming results will be improved by focusing on the swimming technique.

### P29 The relationship between physical inactivity and body mass index among students in Novi Sad

#### Branislava Teofilovic^1^, Dusica Rakic^1^, Nevena Grujic Letic^1^, Emilia Gligoric^1^, Aleksandar Takaci^2^, Daniela Kenjeric^3^

##### ^1^Faculty of Medicine, University of Novi Sad, Serbia; ^2^Faculty of Technology, University of Novi Sad, Serbia; ^3^Faculty of Food Technology Osijek, Josip Juraj Strossmayer University of Osijek, Croatia

###### **Correspondence:** Branislava Teofilovic (branislava.teofilovic@mf.uns.ac.rs)

**PURPOSE**: The aim of this paper was to determine the connection between body mass index and level of physical inactivity among students in Novi Sad. **METHODS**: The research was conducted in 2018 (April-May) and included 514 students. Students aged from 19 to 24 (133 males and 381 females) and were from 4 faculties whose study programs contain nutritional items. Nutrition status was assessed based on body mass index (BMI). The original anonymous questionnaire was used. Statistical processing was done in SPSS20. **RESULTS**: More than two-thirds of students (80%) had a good BMI. 14.4% were overweight and obese, and 5.6% of students suffered from malnutrition. Male students had a greater tendency to be overweight and obese and sex differences were statistically significant (p <0.01). Majority of students were physically active more than 1h during the day and overweight and obese students were the most active, with statistical significance (p<0.05). Over 80% of students spent more than 1h in front of the monitors. **CONCLUSION**: Preventive programs should focus on increasing the level of physical activity and reducing sedentary lifestyles, and this will play a key role in preventing obesity.

### P30 Antioxidant extracts of willow bark as potential ingredients of weight management products

#### Emilia Gligoric, Ljiljana Suvajdzic, Branislava Teofilovic, Nevena Grujic-Letic

##### Faculty of Medicine, University of Novi Sad, Serbia

###### **Correspondence:** Emilia Gligoric (emilia.sefer@mf.uns.ac.rs)

.

### P31 Effects of differently designed training and strength on the improvement of correlations between extensor and flexor strength in knee crank

#### Dzenana Imamovic, Nedim Covic

##### Faculty of Sports and Physical Education, University of Sarajevo, Bosnia and Herzegovina

###### **Correspondence:** Dzenana Imamovic (imamovicdzenana@gmail.com)

**PURPOSE**: The aim of this research was to determine the effects of differently designed training and absolute strength on the improvement of correlations between extensor and flexor strength in knee crank. **METHODS**: There was a prospective randomized study made which included 44 students aged between 19 and 23 divided into two groups. Group 1 (n=22) was made of examinees that realized development program of no dominant strength through skill training and proprioceptive training (15 weeks, 3 times a week) while group 2 (n=22) had a program focused to develop absolute strength (15 weeks, 3 times a week). **RESULTS**: High difference effect was noted for dominant leg P=0.001 (52.31±6.43% in initial measurement and 58.55±6.20% in final measurement). Improvement in a proportion of extensor and flexor strength of no dominant knee was significantly higher in the group that developed absolute strength (F3,40=.059; p=.809; η²p=.001), while dominant leg did not have a notable difference in effects of two applied programs. **CONCLUSION:** Training of strength in no dominant side of the body, as well as absolute strength training, had an impact on extensor and flexor strength improvement in knee area as well as in-between proportion strength in both legs. Absolute strength training was more efficient to improve the strength of no dominant side when it comes to training of skills and proprioception.

### P32 Forest aerial adventure parks of Romania: new possibilities to develop the human physical capacity

#### Bela Jozsef Balla, Julianna Boros-Balint

##### University of Babes-Bolyai, Faculty of Physical Education and Sport, Cluj-Napoca, Romania

###### **Correspondence:** Bela Jozsef Balla (balla_bela_jozsef@yahoo.com)

One of the primary goals of physical education is to improve human physical capacity. The physical capacity is a dynamic potential of a person, which is composed of the level of motor abilities and motor skills acquired during life. School-based physical education plays an important role in the development of motor capacity, but it is by far not enough to achieve the goal of physical education. **PURPOSE**: The purpose of this study is to map the adventure parks of Romania and to examine (by reviewing the literature) the effect of climbing on the motor capacity of the participants. We would like to examine the most common obstacles in adventure parks and determine what physical abilities are needed to complete them. **CASE PRESENTATION**: In Romania, the first forest adventure park was built in 2006 and by now, their number has reached thirty. Their main characteristic is that the climbers, by relying on their own physical and mental abilities, try to cross obstacles of various difficulty, height, and length. **CONCLUSIONS**: Moving activities provided by forest adventure parks have become easily available to more and more people. Climbing in adventure parks (and climbing in general) can be an excellent alternative to improving motor capacity, as it uses and develops the body in a versatile way.

### P33 The relation between dynamic balance and level of Alpine skiing knowledge

#### Vjekoslav Cigrovski, Tomislav Rupcic, Mateja Ocic, Ivan Bon, Vedran Dukaric

##### Faculty of Kinesiology, University of Zagreb, Croatia

###### **Correspondence:** Ivan Bon (ivan.bon@kif.hr)

The well performed alpine ski turn is the one during which a skier maintains optimal central position during all phases of a turn. This is the reason why balance is incorporated in the process of alpine ski learning as well as training from the beginning phases. Lack of balance increases the risk of injuries and has detrimental effects on ski technique. **PURPOSE**: We aimed to investigate if there is a correlation between laboratory tested dynamic balance and level of alpine skiing knowledge. **METHODS**: This research included 91 participants (27 females and 64 males; average age 22±3 years). Participants (178.04±7.71cm, 76.93±12.88kg) were students of Faculty of Kinesiology, and ski novices. Balance assessment was performed in laboratory conditions on the constructed instrument. After balance testing, participants were included in 10-days structured alpine ski school. On the last day, alpine skiing knowledge of each participant was assessed by independent judges on a scale 1–5. **RESULTS**: Dynamic balance test conducted in laboratory conditions did not show significant correlation with alpine skiing knowledge assessment (r=0.01, p=0.91). **CONCLUSION**: Balance is not the only motor ability crucial for the learning process of alpine skiing. A probable reason for such a result is the unreliability of laboratory test used for assessment of balance. Balance is one of the major contributors in alpine skiing knowledge but needs to be assessed in more specific conditions.

### P34 Differences in body composition between top marathon runners and 10,000-meter runners

#### Ilona Mihajlovic, Nikola Radulovic, Milan Solaja

##### Faculty of Sport and Physical Education, University of Novi Sad, Serbia

###### **Correspondence:** Nikola Radulovic (nikolardulovicfsfv@gmail.com)

There is no doubt that morphological characteristics are an important factor of success in sports activities. **PURPOSE**: To determine the differences in some anthropometric characteristics of top-ranking 10,000-meter runners and marathon runners. **METHODS**: The sample of respondents consisted of 30 finalists on the 10,000-meter run and 30 best marathon runners at the Summer Olympic Games in Rio de Janeiro in 2016. Data were downloaded from the official website. Body weight (BW), body height (BH) and body mass (BMI) index were used to assess the anthropometric characteristics. The data were processed by using ANOVA and MANOVA. **RESULTS**: There is no statistically significant difference at the level p=.75 between marathon runners and 10,000-meter runners. It can also be observed that statistically significant differences do not exist in any single variable between these two groups of respondents. **CONCLUSION**: The results of this study showed no statistically significant differences between top marathon runners and 10,000-meter runners in body composition. Even the marathon is four times longer than the distance of 10,000 meters, both athletic disciplines are of long-distance nature and competitors are similar in regards to anthropometric characteristics.

### P35 Predictability of abdominal obesity indicators for assessing general obesity in adolescents

#### Rada Rakic^1^, Tatjana Pavlica^1^, Otilija Herubel^2^

##### ^1^Department for Biology and Ecology, Faculty of Sciences, University of Novi Sad, Serbia; ^2^Primary School "Prva vojvodjanska brigada", Novi Sad, Serbia

###### **Correspondence:** Rada Rakic (rada.rakic@dbe.uns.ac.rs)

The indicators of abdominal obesity show various discriminative power for predicting general obesity. **PURPOSE**: To assess discriminative power of waist circumference (WC), waist-to-hip ratio (WHR), waist-to-height ratio (WHtR), conicity index (CI) and abdominal volume index (AVI) for assessing general obesity. **METHODS**: 265 boys and 253 girls aged 11-15 were surveyed. General obesity was assessed using the cut-off values of the body mass index proposed by the International Obesity Task Force. **RESULTS**: waist circumference and WHtR and AVI indices proved to be excellent indicators of general obesity in both boys and girls (AUC ranging 0.943- 0.974 in boys and 0.930- 0.935 in girls). The WHR and CI indices proved as very good predictors in boys (0.805 and 0.804, respectively) and good predictors in case of girls (0.784 and 0.741, respectively). The optimal cut-offs of WC, WHR, WHtR, CI and AVI were 81.50 cm, 0.898, 0.486, 1.24 and 12.88 cm^2^ in boys and 77.7 cm, 0.900, 0.453, 1.22 and 12.11 cm^2^ in girls. **CONCLUSION**: WHtR, AVI, and WC are found to be more superior than WHR and CI for assessing general obesity of boys and girls.

### P36 A longitudinal study of domain-specific self-perceptions in student-athletes

#### Tatjana Tubic (tubic@uns.ac.rs)

##### Faculty of Sport and Physical Education, University of Novi Sad, Serbia

Previous cross-sectional studies suggest that students' self-perception may be predictive for a wide range of activities, including academic achievement and active lifestyle. **PURPOSE**: The aim of this longitudinal study was twofold: a) to examine student-athletes' domain-specific self-perceptions change over four-year education, and b) to examine whether their self-perception differs by gender, and/or sports-related variables. METHODS: A total of 42 students (25 males and 17 females) completed the Self-Perception Profile for College Students, as well as the background questions related to gender and sports experience, at the beginning of studies and four years later, at the end of their studies on the Faculty of Sport and Physical Education, University of Novi Sad. **RESULTS**: The repeated MANOVA indicates that the impact of between subjects’ variables (gender, individual/team sport, and level of sports engagement) on within subjects’ variables (domain-specific self-perceptions) across the two-time points are not statistically significant. Paired sample t-tests for each domain of self-perception separately show that student-athletes at the end of studies had higher perceptions of their Scholastic Competence (t(41)=-3.006, p=.005) and Physical Appearance (t(41)=-2.573, p=.014) than four years before, while Global Self-Worth (t(41)=3.021, p=.004) decreases during the same time. **CONCLUSION**: This research highlights the importance of developing effective strategies for enhancing relevant specific domains of self-perception required for future professional challenges.

### P37 Effects of indoor rowing exercise on balance, flexibility, reaction time and muscle strength in older adults

#### Khaothin Thawichai, Rachnavy Pornthep

##### School of Sports Science, Suranaree University of Technology, Nakhon Ratchasima, Thailand

###### **Correspondence:** Khaothin Thawichai (thawichai.khaothin@gmail.com)

Among the numerous physical activities, rowing is a whole-body strength-endurance sport with an aerobic and anaerobic component. There are very few studies that focus on indoor rowing exercise on balance, flexibility, reaction time and muscle strength in older adults. **PURPOSE**: To examine the effects of indoor rowing exercise on balance, flexibility, reaction time and muscle strength in older adults. **METHODS**: The participants were purposive random sampling collected from active lifestyle older adults in Phoklang Nursing Home, Muang, Nakhon Ratchasima, Thailand (male: 10; female: 10). All participants were tested for body weight, height, body mass index, and physical fitness component included balance, flexibility, reaction times, and muscular strength before and after 8-weeks indoor rowing exercise. The rowing ergometer was set up 5 days a week; each session included a 10-minute warm-up, a 20-minute rowing exercise, and a 10-minute cooling-down period. Exercise intensity was 40%–50% of 1 RM during the 2-week adaptation period and 50%–60% during the next 6-weeks. **RESULTS**: The results of this study show that balance, flexibility, reaction times, and muscular strength after 8-week indoor rowing exercise were significant differences higher than their baseline. **CONCLUSION**: The finding indicates that indoor rowing exercises more benefit on balance, flexibility, reaction time and muscle strength in older adults.

### P38 The quasi-experiment study on the health empowering program mentoring behavior modification toward physical wellness in young adults through group advisor service

#### Chantima Weerapon (thawichai.khaothin@gmail.com)

##### School of Sports Science, Suranaree University of Technology, Nakhon Ratchasima, Thailand

This quasi-experiment study evaluated the effectiveness of empowering young adults for behavior modification through group advisory service via counselor and consultant using the Health Empowerment Program (HEP). The aim of this study was to determine the comparison outcome on the causes and effects of the changes in student's physical fitness between groups in the period of 12 weeks. The subjects were the students from the Suranaree University of Technology, Thailand. The sample included 60 over-weight students, with equal-size for control and experimental group of 30 subjects. The experimental group received HEP service sessions intended to modify health behaviors, exercise routines, and food consumption habits. The control group lived on its own routine lifestyle. The results from the study suggested that the self-efficacy empowering program generated by the advisory services in a group of undergraduate students, with a tendency towards obesity relating to over consuming nutrition, can effectively modify lifestyles behaviors, including exercise, food consumption habits, and wellness.

### P39 Volleyball as a one-year optional activity of the final grade male students in elementary schools

#### Milovan Ljubojevic, Ivan Vasiljevic, Rajko Milasinovic, Danilo Bojanic

##### Faculty for Sport and Physical Education, University of Montenegro, Montenegro

###### **Correspondence:** Milovan Ljubojevic (milovan.lj@ucg.ac.me)

**PURPOSE**: Efficiency of physical education can be improved with real, professional, and economic planning and monitoring of education effects, as well as with an increase in the weekly number of classes. **METHODS**: The study was conducted for one school year on the sample of a total of 43 male students, divided into two groups. The control group (22 students) consisted of students who attended only physical education classes. The experimental group (21 students) consisted of students who, in addition to physical education classes, attended an additional two classes of volleyball during a week. Motor space was monitored through 8 variables of Eurofit battery tests. **RESULTS**: After the conducted study, results showed that the students from the control group had better results in 6 out of the total 8 motor tests. In test "pull-up endurance" and "pin running on 10x5m" no significantly better results were observed. The students of the experimental group achieved better results in 5 out of the total 8 tests. The improvement was not observed in the tests "seated forward bend", "pull-up endurance" and "pin running on 10x5m". **CONCLUSION**: The analysis of the results showed that the experimental volleyball program had some influence, but not to the extent that it was expected from two additional hours of volleyball in physical education.

### P40 Benefits of a controlled physical activity program

#### Ivan Bon, Mateja Ocic, Vedran Dukaric, Damir Knjaz

##### Faculty of Kinesiology, University of Zagreb, Croatia

###### **Correspondence:** Ivan Bon (ivan.bon@kif.hr)

Diabetes and obesity-related health issues are exponentially growing during the last century. Sedentary and fast living life affects the quality of life. Half of the top 10 global causes of death are related to low daily activity. It is proven that active adults have a lower mortality rate, coronary heart diseases, high blood pressure, risk of cardiac attack, diabetes, colorectal and breast cancer. WHO states that regular and controlled by expert physical activity is one of the best prevention tools for above mentioned chronical illnesses. **PURPOSE**: Aim of this research is to determine whether continuous and programmed physical exercise affects morphological and motor abilities condition of physically inactive individuals. **METHODS**: This research included 229 previously inactive individuals (177 female and 52 male; age of 49.12 ± 9.38). All participant went through testing of morphology status (weight, body fat percentage, muscle mass) and motor abilities (push-ups, squats, lunges, wall seat, plank) before and after programmed physical exercise (3 months duration, 24 training sessions). **RESULTS**: Results of morphological status after programmed physical exercise significantly improved (p=0.00). Results of motor abilities also showed a significant difference between initial and final physical condition (p=0.00). **CONCLUSION**: Results of this research showed that with programmed and systematic physical exercise twice per week it is possible to significantly improve body composition and physical condition of previously inactive individuals.

### P41 Testing the influence of destination image and sports event quality on athletes intentions’ for repeated destination visit: the cases of three World Sambo Championships in Novi Sad, Serbia

#### Radenko Matic^1^, Ivana Milovanovic^1^, Kostas Alexandris^2^, Nebojsa Maksimovic^1^, Stevo Popovic^3^, Rajko Bujovic^4^, Patrik Drid^1^

##### ^1^Faculty of Sport and Physical Education, University of Novi Sad, Serbia; ^2^Laboratory of Sport, Tourism and Recreation Management, Aristotle University of Thessaloniki, Greece; ^3^Faculty for Sport and Physical Education, University of Montenegro, Montenegro; ^4^Faculty of Business Studies, Mediterranean University Montenegro, Podgorica, Montenegro

###### **Correspondence:** Radenko Matic (radenkomatic@uns.ac.rs)

**PURPOSE:** This study aimed to test the influence of destination image and event quality on athletes' intentions for repeated destination visit with the mediation role of destination quality. **METHODS:** The study included N=350 elite sambo athletes from World Sambo Championships, Novi Sad, Serbia (2017-2018). Destination image was measured with two dimensions: affective and cognitive. Event quality was measured with 12 items, destination quality with 10 items and behavioral intentions with 3 items (Alexandris et al., 2018). **RESULTS:** The Structural Equation Modeling analysis run with AMOS showed acceptable fit: χ^2^ = 8205, df = 1980, χ^2^/df = 4.14, CFI = 0.92, NFI= 0.91, and RMSEA = 0.07, which predicted 84% of variance intentions to revisit (R^2^=0.84). Destination loyalty is related to the core dimension of event quality at all levels of sports experiences. Though related to an affective image, still these relationships are mediated by destination quality. **CONCLUSION:** Research shows that sports event from its opening through a complete organization phase contributes to destination loyalty.

### P42 Biomechanical comparison of single- and double- leg landings during 3-point jump shot in basketball

#### Rachnavy Pornthep^1^, Khaothin Thawichai^1^, Rittiwat Wacharee^2^

##### ^1^School of Sports Science, Suranaree University of Technology, Nakhon Ratchasima, Thailand; ^2^Faculty of Sport and Physical Education, Srinakharinwirot University, Bangkok, Thailand

###### **Correspondence:** Rachnavy Pornthep (rachnavy@sut.ac.th)

Knee injuries are common in basketball players, especially ACL injuries, which are considered serious injuries for athletes. **PURPOSE:** To compare the biomechanical characteristics of single-leg landing and double-leg landing during basketball 3-point jump shot. **METHODS:** Ten male basketball athletes ages 18-30 participated in the study. A set of spherical retro-reflective markers placed on their body segments to measure movement performance. The landing motion was measured using the QTM™ motion system. Participants performed single-leg and double-leg 3 point jump shot landing tests. Subjects landed on Kistler force plate during all performance trials. Paired t-tests were used to compare the kinematic and kinetic variables of the knee joint and ground-reaction forces between the single-leg and double-leg jump shot during the landing. **RESULTS:** Statistically significant differences between groups were found for the vertical ground reaction force, knee joint angles, knee valgus angle and knee angular velocity. For the single leg during landing, the subjects’ knees were less flexed and less angular velocity but greater knee valgus angle and vertical ground reaction force (p<0.05). **CONCLUSION:** Increased knee valgus angle during single-leg 3 point jump shot landing in basketball may reflect the greater ACL injury. It suggests that Injury prevention should be landing with a good technique.

### P43 Model of work – the factor in swimming instruction effectiveness

#### Drazen Rastovski, Jurica Lovrincevic

##### Faculty of Education, J. J. University Osijek, Croatia

###### **Correspondence:** Drazen Rastovski (drastovski@foozos.hr)

**PURPOSE**: To determine the effectiveness of particular models of teaching non-swimmers and compare the tested population of 201 children aged 9 to 11 years. **METHODS**: The sample was divided into two subgroups in which the children were taught how to swim using the same program, but a variety of models. The first model (1) was a model of teaching non-swimmers in the summer school for a period of 14 days, while the other model (2) was implemented within the school program and lasted 6 days. The number of lessons for the swimming instruction for both groups was the same (20 hours). To determine the effectiveness of these models a statistical software package STATISTICA was used. To compare the progress of the groups we used the Wilcoxon Matched Pairs Test and for comparison between the groups Mann-Whitney U-test P<0.05. **RESULTS**: The research showed that both programs yielded significant progress but it was also established that type 1 instruction in which children were taught to swim during summer school was more successful. **CONCLUSION**: Both programs produce good results and are suitable for conducting swimming instructions. We recommend model 1, where swimming instructions are completed during summer school. Model 2 of swimming instructions is conducted during school in nature and it is completed in six days and obviously in lack of time for even better implementation.

### P44 Leisure time physical activity among children in Vojvodina

#### Sonja Cankovic^1,2^, Vesna Mijatovic Jovanovic^1,2^, Natasa Dragnic^1,2^, Ivana Radic^1,2^, Tanja Tomasevic^1,2^, Dusan Cankovic^1,2^, Sanja Harhaji^1,2^

##### ^1^Faculty of Medicine, University of Novi Sad, Serbia; ^2^Institute of Public Health of Vojvodina, Novi Sad, Serbia

###### **Correspondence:** Sonja Cankovic (sonja.cankovic@mf.uns.ac.rs)

According to the World Health Organization children and youth should accumulate at least 60 minutes of moderate to vigorous intensity physical activity daily. Physical inactivity is considered as one of the major risk factors for many chronic diseases. **PURPOSE**: To analyze the prevalence of physical inactivity in leisure time among children in Vojvodina and to determine the association between socio-demographic factors and physical inactivity. **METHODS**: Data were obtained from the National Health Survey Serbia, cross-sectional study conducted in 2013. The sample included 332 children aged 7-14 in Vojvodina, 47.3% males and 52.7% females. Physical inactivity was examined as having physical exercise/sports in leisure time 1-2 times per week or less. **RESULTS**: During leisure time, only 37.7% of children were having physical exercise/sports almost every day, almost two-fifths (38.6%) were physically active only 1-2 times per week and one-fourth (23.8%) were rarely or never physically active. Girls had almost two times higher odds for physical inactivity (OR=1.88; 95%CI=1.19-2.97) than boys and children from rural area**s** (OR=1.73; 95%CI=1.01-2.74) compared to urban. There was no influence of other variables (age and household wealth). **CONCLUSION**: The results show a high level of leisure time physical inactivity among children in Vojvodina, particularly among girls and children from rural area**s**.

### P45 Morphological changes in boys with special needs after physical education

#### Danilo Bojanic, M Ljubojevic, R Milasinović, I Vasiljevic

##### Faculty for Sport and Physical Education, University of Montenegro, Montenegro

###### **Correspondence:** Danilo Bojanic (danilo.bo@ucg.ac.me)

The need which man has for physical activity is one of the requirements for survival. Physical education as an integral part of the educational process in schools has the basic task of using positive kinesiological operators to influence positive transformation processes. The population of students with special needs represents one of the links in the chain of complex educational and systematic social influence in physical education to which new generations are subjected. **PURPOSE**: This research was aimed at showing how and in what way different kinesiological operators can influence the transformation of the anthropological status of students with special needs. **METHODS**: The research has been conducted on the students of the Center for Children and Youth with Special Needs in Mostar, the Center Los Rosales Mostar and the Facilities for daily living for children with special needs in Niksic. The sample consisted of 46 boys. Nine variables were tested: breast circumference, upper arm circumference, abdominal volume, upper leg circumference, lower leg circumference, scapula skin fold, upper arm skin fold, abdomen skin fold, lower leg skin fold. **RESULTS**: The applied curriculum of physical education and sports contributed to the change in tested parameters. **CONCLUSION**: The obtained results should contribute to better planning, programming, directing and controlling the effects of physical education and sports on the population of students with special.

### P46 A socio-demographic aspect of cycling as transportation and its relation to health

#### Vesna Mijatovic Jovanovic^1,2^, Dragana Milijasevic^1,2^, Ivana Radic^1,2^, Sonja Cankovic^1,2^, Sanja Harhaji^1,2^, Tanja Tomasevic^1,2^

##### ^1^Faculty of Medicine, University of Novi Sad, Serbia; ^2^Institute of Public Health of Vojvodina, Novi Sad, Serbia

###### **Correspondence:** Vesna Mijatovic Jovanovic (vesna.mijatovic-jovanovic@mf.uns.ac.rs)

Using a bicycle for transportation can contribute to an increase in total physical activity and thus a beneficial effect on health. **PURPOSE**: To determine the socio-demographic differences in cycling during transports in the working population in Vojvodina as well as to assess its association with health. **METHODS**: Data from the National Health Survey in Serbia 2013 was analyzed on a sample of 2527 participants aged 20-65 years in Vojvodina. **RESULTS**: The third of participants (35.2%) used a bicycle for transport at least 5 times a week, 11.6% - 3 to 4 times, while 43.0% never used it. Among those who have never ridden a bicycle, were a significantly more urban population (46.2%; p<0.001), the category of rich (55.9%; p<0.001) and participants with high education (57.4%; p<0.001). Analysis showed that participants who did not ride a bicycle had a 2.3 times higher chance of assessing their health as poor (OR=2.3; 95% CI=1.55-3.36) compared to those who were cycling more than 150 minutes a week. **CONCLUSION**: Bicycle as a means of transport in Vojvodina is significantly less used by respondents in the urban area as well as persons of higher social status. People who do not cycle have a significantly higher chance of assessing their health as poor.

### P47 Physical activity and self-rated health: a population-based study in Vojvodina

#### Ivana Radic^1,2^, Mirjana Martinov Cvejin^1^, Sanja Harhaji^1,2^, Sonja Cankovic^1,2^, Natasa Dragnic^1,2^, Dusan Cankovic^1,2^

##### ^1^Faculty of Medicine, University of Novi Sad, Serbia; ^2^Institute of Public Health of Vojvodina, Serbia

###### **Correspondence:** Ivana Radic (ivana.radic@mf.uns.ac.rs)

Physical activity has many documented health benefits and can be undertaken in different domains of life. **PURPOSE**: To estimate the association between physical activity in different domains with self-rated health in the adult population. **METHODS**: The study is part of the National Health Survey Serbia, a cross-sectional study conducted in the year 2013 by the Ministry of Health of Republic of Serbia. The sample included 3633 people aged 20 and more from Vojvodina. Levels of physical activity in leisure time, at work, walking and cycling for transportation were assessed by questionnaire. Logistic regression was used to estimate the association between good self-rated health and physical activity variables. **RESULTS**: Bivariate analysis indicated that higher levels of physical activity in all three domains (leisure time, transportation, work) were associated with a better assessment of health. Multivariable logistic regression analysis showed that leisure-time physical activity (OR=1.65) and walking for transportation (OR=2.65) 150 minutes and more weekly were important predictors of good self-rated health. Participants who were physically active at work were 1.31 times more likely to assess their health as good/very good compared to those who were sedentary. **CONCLUSION**: Physical activity in leisure time, at work and walking for transportation are associated with good self-rated health, after adjustment for age, gender, and educational level.

### P48 Influences of occupational therapy as curricular activity on to psycho-physical development of hearing-impaired students

#### Rusu Oana (broana@uaic.ro)

##### Faculty of Physical Education and Sport, "Alexandru Ioan Cuza" University of Iasi, Romania

**PURPOSE**: We intend to identify the effects of occupational therapy means on self-esteem and on the psycho-motor development level for hearing**-**impaired students. **METHODS**: Two instruments (test-retest at an 18-month interval) have been used for the hearing**-**impaired pupils of the "Vasile Pavelcu" Technological Special High School in Iasi: the scale of multidimensional attitudes for self-esteem E.T.E.S. (N=45 subjects, 24 boys and 21 girls, aged between 13 and 18) in elementary and high school, each groups of 15 subjects; the test Ozeretski – Guillmain (N= 45 subjects: 25 boys and 20 girls, aged between 8 and 12) in primary and elementary school. The individual test applied for assessment of psycho-motor development level. The subjects for whom we have assessed the self-esteem attended activities that included pantomime/puppet theater, manual and sports skills activities. The subjects for whom we have assessed the psycho-motor development level have attended the following activities: manual skills, painting, and sports activities. All activities were attended following a preset graph. **RESULTS**: There is a significant statistical difference to subjects in the phase of retesting to the testing phase for all the variables taken into account: social self-esteem, prospective self-esteem, the total level of psychomotor development. **CONCLUSIONS**: Self-esteem and the level of psycho-motor development are two variables that can improve the psycho-physical state of children with hearing impairment through occupational therapy.

### P49 Effects of four-month football training on the motor skills of the younger school-age children

#### Vedran Gajski, Zvonimir Tomac, Hrvoje Ajman

##### Faculty of Education, J.J. Strossmayer University of Osijek, Croatia

###### **Correspondence:** Zvonimir Tomac (ztomac@foozos.hr)

It is recommended to encourage children from their early school age to various sports activities to improve their growth in general. **PURPOSE**: The influence of the four-month football-specific training on functional and motor abilities of the boys aged 7 to 10 years were tested. **METHODS**: A study sample was formed of 18boys. A sample of variables was made of two morphological tests, six motor skills tests and one test of functional capacities. The four-month program of football has consisted of 53 practices, 4 times per week averagely. Wilcoxon matched pairs test was used for estimating differences between initial and final measures. **RESULTS**: The results of the study determined statistically significant positive effects on the morphological characteristics and functional capacities of participants. Also significant positive effects were marked on the motor skills, balance (T=12.00; Z=3.05, p=0.00), explosive power of lower extremities (T=24.50; Z=2.66, p=0.01) and coordination (T=18.00; Z=2.77, p=0.01), and in aerobic tests (T=200, Z=3.64, p=0.00). Statistically significant positive effects were not determined in agility, flexibility and repetitive power of the front side of the trunk. **CONCLUSION**: Various extracurricular activities improve student development and enhance their growth and development. This study points out the important role of sports activities for children due to a positive influence not only on motor skills but also on growth, development, socialization and physical health.

### P50 Effect of exercise on menopausal symptoms and sexual functions

#### Annamaria Pakai^1^, Andras Olah^1^, Pongrac Acs^1^, Timea Csakvari^1^, Novak Evelin^2^

##### ^1^Faculty of Health Sciences, University of Pecs, Hungary; ^2^Heart Institute, University of Pecs, Hungary

###### **Correspondence:** Annamaria Pakai (annamaria.pakai@etk.pte.hu)

**PURPOSE**: The aim of this study is to assess the habits of women with post-menopause, symptoms related to lack of exercise and presence of sexual dysfunctions. **METHODS**: A descriptive, quantitative study was carried out in Baranya county, Hungary between August 2017. – January 2018. Our target group consisted of post-menopausal women between the age of 40-65, with an active sexual life (N=102). Standardized questionnaires (FSFI, MENQOL, Godin-Shephard leisure) were used for data collecting. Descriptive statistics, correlation, regression analysis, χ^2^– and independent samples t-test were calculated with SPSS 20.0 software (p<0.05). **RESULTS**: Mean age was 50.56±4.35 years, 55.58% of women had reached the post-menopausal stage. Mean BMI was 25.93±4.06 kg/m^2^. 47.06% of women exercises regularly, they spend 4.27±2.8 hours/week on average. Mean value of Godin-Shephard leisure scale was 28.02 ±18.68 among women with an active life. Respondents got 26.88±5.34 points on FSFI questionnaire, 47.06% of them mentions some kind of sexual disorder. FSFI values are higher among women who exercise and the risk of developing sexual dysfunction is lower (p<0.05). **CONCLUSIONS**: Exercise can be a contributing factor to both physical and mental health of post-menopausal women. An active way of life has a beneficial effect on sexual functions, it reduces the number of menopausal symptoms and their intensity, reacting to the development of sexual dysfunction as well.

### P51 Influence of extracurricular and out-of-school sports activities on elementary school students

#### Tihomir Vidranski, P Otkovic, M Hrga

##### Faculty of Education, University of Josip Juraj Strossmayer, Osijek, Croatia

###### **Correspondence:** P Otkovic (potkovic@foozos.hr)

The active leisure time for students can be supplemented by attending extracurricular and out of school sports activities. **PURPOSE**: To determine if attending extracurricular and out-of-school sports activities influence students’ motor skills. **METHODS**: 94 students (47 male) from first and third grade (45 from first and 49 from third grade) were tested in three motor skill tests according to CROFIT: run across with sponges (MAGPRP); bend the upper body from sitting position (MFLPRU); raising of the torso from a lying position (MRSPTL). The height and weight, as well as the body mass index, were also measured. Out of the total sample, 25 students were in the group engaged in extracurricular and out of school activities (G1), while 23 students were in the group engaged only in out of school activities (G2). In the group engaged only in extracurricular activities (G3) were 23 pupils. There were 23 pupils in the group (G4) which was not engaged in either extracurricular or out of school activities. **RESULTS**: G1 performed better in all motor skills tests and results were superior as compared to G4. G2 performed better than G4 and G3 achieved better results in MAGPRP then group G4. **CONCLUSION**: Additional physical activities have great impacts on the development of motor skills for elementary school pupils.

### P52 A pilot study about the development of visual-motor memory and segmental coordination at physical education students from Timişoara

#### Mihaela-Liana Faur, Corina Pantea

##### Faculty of Physical Education and Sport, West University Timisoara, Romania

###### **Correspondence:** Mihaela-Liana Faur (mihaelafaur@yahoo.com)

**PURPOSE**: The study aims to find solutions in order to improve the educational activities in vocational colleges – field of Physical Education and Sport. **METHODS**: The study included 43 physical-education students. They were subjected to two tests. The memory test consists of an eight times exercise which every student had to memorize and to reproduce it correctly. The segmental coordination test consists of a two eight times exercise. All tests are done practically. **RESULTS**: At final testing (Tf) at the memory test 4 female students (22.2%) performed accurately compared with 2 (11.1%) at initial testing (Ti) and 8 of them scored at the second attempt. The segmental coordination test requires more precise control of the movements. There were students who needed 10 to 12 attempts for the correct execution of the structure at Ti as well as at Tf. None performed well at first attempt. At the motor memory test, 6 male students out of 25 (24%) executed correctly at first attempt at Tf compared to 2 students (8%) at Ti. At Tf for segmental coordination, none of the students performed well on the first attempt. **CONCLUSION**: In order to intervene effectively in the process of motor learning and memory stimulation, the activities should be varied and able to apply in a creative way.

### P53 Twelve weeks of in-season FIFA 11+ program improves physical performance in adolescent soccer players

#### Marko Gusic^1^, Nebojsa Trajkovic^1^, Dejan Madic^1^, Slavko Molnar^1^, Slobodan Andrasic^2^, Ivica Franciskovic^3^

##### ^1^Faculty of Sport and Physical Education, University of Novi Sad, Serbia; ^2^Faculty of Economics, University of Novi Sad, Serbia; ^3^Football club Spartak Subotica, Serbia

###### **Correspondence:** Marko Gusic (gusicmarko@yahoo.com)

Effects of FIFA11+ prevention warm-up program on the reduction of injuries are well documented. However, studies concerning the benefits of this program on physical performance are limited. **PURPOSE**: This study aims to investigate the effect of in-season FIFA11+ program on speed, change of direction speed and vertical jump performance in adolescent male soccer players. **METHODS**: Forty-eight male soccer players (mean ± SD: age = 17.79 ± 1.18 years, height = 176.17 ± 7.12 cm, mass = 63.35 ± 9.01 kg, experience = 7.76 ± 1.36 years) from FC Spartak Subotica participated in this study and were divided into two groups, FIFA11+ and control. The experimental group performed the FIFA11+ program three times per week for 12 weeks whereas the control group just performed their regular warm-up program. **RESULTS**: FIFA11+ group showed improvements in vertical jump performance, however with only significant improvement in CMJ (p=0.01). Intervention group improved 5m sprint (p=0.05), 10m sprint (p=0.01) and 20-m sprint (p=0.01). The intervention group also improved their performance on Illinois test (p=0.01). No changes were found in the control group. **CONCLUSION**: The results suggest that FIFA11+ program can be used as an effective conditioning means for improving physical performance in adolescent soccer players.

### P54 The relationship between repeated sprint ability and aerobic capacity in adolescent soccer players

#### Slavko Molnar^1^, Marko Gusic^1^, Slobodan Andrasic^2^, Nebojsa Trajkovic^1^, Danilo Radanovic^1^, Milan Cvetkovic^1^

##### ^1^Faculty of Sport and Physical Education, University of Novi Sad, Serbia; ^2^Faculty of Economics, University of Novi Sad, Serbia

###### **Correspondence:** Slavko Molnar (molslavko@gmail.com)

There is a considerably higher amount of research dealing with repeated sprint ability (RSA) and aerobic capacity in soccer recently. However, due to numerous RSA tests and VO2max determination tests, there is a contradiction in the past years. **PURPOSE**: The aim of this study was to determine the relationship between RSA and aerobic capacity in adolescent soccer players. **METHODS**: Ninety-two well-trained adolescent soccer players (age:16.6±1.3yr; height:180.49±5.3cm; mass:70.12±7.3kg) were involved in this study. They were tested for RSA test consisted of 6x40m(20m+20m) shuttle sprints separated by 20s of passive recovery and the VO2max was calculated during the 30–15 Intermittent Fitness Test. The fastest time in a single sprint (RSAbest), mean time (RSAmean) and percent decrement (RSAdec) during the RSA test were determined. The relationships between VO2max and RSA performance were calculated using Pearson's correlation coefficient. **RESULTS**: We found moderate correlations for VO2max and RSAmean (r=-0.534; p=0.001) and significant correlation between VO2max and RSAbest (r=-0.366; p=0.022). However, no relationship was found between VO2max and RSAdec. **CONCLUSION**: Results from the correlation analyses suggest that VO2max could be considered as an important determinant of RSA performance in adolescent soccer players. Our findings suggest that to improve RSA, adolescent soccer players could benefit from training for aerobic capacity improvement.

### P55 Training process in the development of strength in gymnasts

#### Ikonomi Edison, Kapedani Kujtim

##### Sports Department, Faculty of Movement Sciences, Sports University of Tirana, Albania

###### **Correspondence:** Ikonomi Edison (edisikon@yahoo.com)

Strength is one of the most important motor skills for gymnasts. It is characterized by muscle features to develop strain while performing gymnastics exercises. In this study, we analyzed thirty-two male gymnasts from five sports clubs in different cities of Albania, who were members of the gymnastics competition at the national level. Strength was evaluated with six tests for the period from November 2017 (T.I.) to November 2018 (T.F.). Strength was developed with a dynamic and static method. The test results are presented: (a) legs flexion upon the upper body during 30 sec. T.I. 18.25 ± 3.007 vs T.F. 19.3 ± 3.08 no. of reps., *p* ≤ 0.001; (b) upper body extension upon the legs during 30 sec. T.I. 21.7 ± 2.12 vs. T.F. 23.03 ± 2.22 no. of reps., *p* ≤ 0.002; (c) Deep squat one leg during 30 sec. T.I. 16.7. ± 2.11 vs. T.F. 18.2 ± 2.33 no. of reps., *p* ≤ 0.001; (d) broad jump T.I. 253.44 ±14.723 vs T.F. 257.07 ± 14.12 cm, *p* ≤ 0.005; (e) running 30 meters T.I. 4.276 ± 0.18 vs T.F. 4.105. ± 0.1674 s, *p* ≤ 0.001; and (f) stand L in parallel T.I. 39±6.283 vs. T.F. 43.81± 5.313 s, *p* ≤ 0.001. Tests have shown changes in the level of development of this ability. Changes were found in 71.9% subjects while 9 athletes (28.1%) demonstrated low levels of strength. High level of strength raises performance in training gymnasts and their results in competitions. In a long and hard process of prepare the gymnasts, qualitative systematic testing of strength is available for examination of their situation in each training period.

### P56 Changes in maximum oxygen consumption of wrestlers

#### Kapedani Kujtim, Ikonomi Edison

##### Sports Department, Faculty Movement Sciences, Sports University of Tirana, Albania

###### **Correspondence:** Kapedani Kujtim (kujtimkapedani@yahoo.com)

**PURPOSE:** In this study, we present the progress of the development of VO2max team "Tirana wrestling sport" during the three months annual training process. **METHODS:** A total of 18 wrestlers involved in this study, with their desire. The data presented are related to some anthropometric measurements (age, weight, height, body mass index.) and measurements of VO2max on the ergo-metric "Monarch" bicycle. For this, it formulated a genuine program to increase the level of VO2max, where the wrestlers will be trained for 12 weeks, with three training sessions a week, 15-20 minutes, performing exercises and aerobic endurance mostly outdoor jogging. The data subjected to statistical processing using the IBM SPSS Statistics software 22 method. The statistical techniques used include: general descriptive analysis, evaluation of data distribution and search hypothesis search through differential techniques between two measurements, using T-test. **RESULTS:** Seeing the results a significant improvement was observed from the first measurement to the second and mainly to the VO2max indicators and specifically: VO2max in l min. with improvement margin of 0.34 l / min. (p ≤ 0.001) and VO2max in ml / kg / min. with improvement margin 4.46 ml/kg / min. (p ≤ 0.005). **CONCLUSION:** In conclusion, the above changes with a growing improvement in aerobic level indicators are a good basis for the continuation of physical preparation (motors) in general and technical results of the race.

### P57 Gross motor coordination in relation to weight status in 6-year old Serbian children

#### Boris Popovic^1^, Nebojsa Trajkovic^1^, Danilo Radanovic^1^, Dejan Madic^1^, Aleksandra Spasic^1^, Dusan Stupar^2^

##### ^1^Faculty of Sport and Physical Education, University of Novi Sad, Serbia; ^2^Faculty of Sport and Tourism, Educons University, Novi Sad, Serbia

###### **Correspondence:** Boris Popovic (borispopovic0803@gmail.com)

Fundamental motor skills are an important determinant of children’s general development. Considering the increasing prevalence of childhood overweight and obesity across the world, great attention should be paid to the coordination level in children with an excessive body mass index (BMI). **PURPOSE**: The aim of this study was to investigate differences in gross motor coordination of 6 years old children with different weight status. **METHODS**: Using a cross-sectional design, data were collected in 182 Serbian preschool children (95 boys, 87 girls) aged 6 (SD ± 0.89) years. Weight status (normal-weight, overweight, obese) was defined according to the International Obesity Task Force BMI cut-off points for children. Gross motor coordination was assessed by means of the Körperkoordinationstest für Kinder (KTK). **RESULTS**: ANOVA showed a significant difference between groups in all tested variables (p<0.01). Obese children were found to result in poorer KTK performances (p<0.01) compared to normal weight children. Comparing normal weight and overweight children, differences were not significant only for test moving sideways (p>0.05). **CONCLUSION**: Childhood overweight and particularly obesity were found to result in poorer KTK performances in 6-year old Serbian children. The most pronounced effect of BMI on gross motor performance was observed for one-legged hopping over a foam obstacle.

### P58 Motivation and physical self-concept as the predictors of physical activity in physical education classes

#### Slobodan Pavlovic^1^, Dragan Marinkovic^2^, Visnja Djordjic^2^, Eric Brymer^3^

##### ^1^Teacher Training Faculty in Uzice, University of Kragujevac, Serbia; ^2^Faculty of Sport and Physical Education, University of Novi Sad, Serbia; ^3^Leeds Beckett University, UK

###### **Correspondence:** Dragan Marinkovic (marinkovic@uns.ac.rs)

Physical activity of students in a physical education class is a significant indicator of motivation and the quality of teaching. **PURPOSE** The research project examined the extent to which motivational orientation of students and physical self-concept contribute to the prediction of the volume and intensity of student physical activity in a physical education class. **METHODS**: The survey included 236 pupils (aged 10±1.4 years; 127 boys and 109 girls). Physical activity was estimated by the volume using the Coach Gear pedometer and intensity using the Suunto pulse meter. Students’ motivational orientation, a modified Self-Regulation Questionnaire was used and for the assessment of the physical self-concept Self-perception Profile for Children were used. **RESULTS:** The obtained results indicate that both models of the predictor are statistically significant (p<0.01), in boys for both criterion variables (volume and intensity of the physical activity), whereas the prediction was absent in girls in both cases. At the level of individual variables, Intrinsic Motivation and Sports Competence have the greatest predictive power. **CONCLUSION:** Physical education classes should be planned to encourage inner motivation and to support basic psychological needs for autonomy, competence, and connection with others.

### P59 Medical rehabilitation and adapted sports activities: case report of a patient with paraplegia

#### Slobodan Pantelinac^1,2^, Ksenija Bosković^1,2^, Snezana Tomasević-Todorovic^1,2^, Snezana Mikulic-Gutman^2^, Aleksandar Knezevic^1,2^, Tijana Spasojevic^2^

##### ^1^Faculty of Medicine, University of Novi Sad, Serbia; ^2^Medical Rehabilitation Clinic, Clinical Centre of Vojvodina, Novi Sad, Serbia

###### **Correspondence:** Slobodan Pantelinac

**PURPOSE:** Persons with disabilities need a well-planned rehabilitation approach both in the professional sense and in resocialization. The aim of the paper was to show that the injuries of the thoracic part of the spine with the spinal cord lesion that resulted in paraplegia, do not necessarily lead to a decrease in the quality of life. **CASE PRESENTATION:** At the age of 42, the patient was admitted to the Medical Rehabilitation Clinic in 2014, two months after surgically treated Th8-Th12 fractures and injuries sustained in a traffic accident in November 2013, in which he participated as a motorcycle driver. On reception, mobile with wheelchairs that he actively used. In the lower extremities, there were no active voluntary movements. A specific exercise was conducted, with the evaluation of the clinical psychologist and supportive advisory work. After the hospital treatment, there are still plegia of the lower extremities. The patient continued the implementation of rehabilitation treatment in ambulatory and home conditions. The patient applied for the Provincial sports games of paraplegia and quadriplegia, where he won three bronze medals. **CONCLUSION:** This clinical case emphasizes the importance of a complex and multidisciplinary approach, both in the early, in the chronic phase of rehabilitation and reintegration in society.

**CONSENT**

Written informed consent was obtained from the patient for publication of this case report. A copy of the written consent is available for review from the Editors of this journal supplement issue.

### P60 The relationship between anxiety and aggression among Hungarian football players

#### Agnes Palvolgyi, Kata Morvay-Sey, Melinda Trpkovici, Pongrac Acs, Andras Olah, Jozsef Betlehem

##### Faculty of Health Sciences, University of Pécs, Hungary

###### **Correspondence:** Agnes Palvolgyi (agipalvolgyi@gmail.com)

During a football game, many situations can generate inner tension. This stress can turn into aggressiveness, which impairs performance. **PURPOSE:** The aim of the study was to investigate the relationship between anxiety (trait and state) and the trait of aggression among football players. **METHODS**: A total of 95 Hungarian football players were tested (age between 13-19) with the Spielberger Trait- State Anxiety Inventory (STAI, 1983), The Anger Expression Scale (1985), and Buss-Perry Aggression Questionnaire (BPAQ, 1992). The Hungarian version of STAI has children (under 16) and adult (above 16) versions. We accepted the results as significant if p<0.05. **RESULTS:** We used Pearson Correlation and found positive correlation between STAI TRAIT and BPAQ TRAIT (r=0.51, p=0.00) and between all the subscales of BPAQ (anger r=0.4, p=0.001; physical aggression r= 0.42, p=0.00; verbal aggression r=0.251, p=0.036; hostility r=0.516, p= 0.00) among children. We also found positive correlation between STAI STATE and hostility (r=0.470, p=0.018) and anger (r=0.398, p=0.049) among adults. **CONCLUSION:** Results showed a positive correlation between trait anxiety and trait aggression, which prove the connection of aggression and trait and state anxiety among football players.

**ACKNOWLEDGMENTS**

The research was carried out with the support of the Human Resource Development Operational Program, EFOP-3.6.2-16-2017-00003: "Creating a Research Network for Recreational and Health Cooperation".

### P61 Special Eurobarometer on sport and physical activity versus International Physical Activity Questionnaire, comparison of two different survey methods

#### Alexandra Makai, Viktoria Premusz, Jozsef Betlehem, Andras Olah, Csaba Melczer, Csilla Filo, Kinga Lampek, Maria Figler, Pongrac Acs

##### Faculty of Health Sciences, University of Pecs, Hungary

###### **Correspondence:** Alexandra Makai

The monitoring the physical activity of the healthy adults has great importance; they face a significantly lower risk of various chronic diseases compared to those with a sedentary lifestyle. **PURPOSE:** The differences among Special Eurobarometer on Sport and Physical activity (EB) and International Physical Activity Questionnaire (IPAQ-L) compared to accelerometer data were examined. **METHODS:** Data were obtained from cross-sectional research among healthy adults in 2018 (N=113), from Hungary. Spearman's rank correlation was used to examine the difference between the EB and IPAQ questionnaires compared to accelerometer data. **RESULTS:** 62 women and 51 men have participated with age of 21.5±1.8 years and BMI 22.0± 5.7 kg/m^2^. The total MET of the EB was 2012.7±1368.3, while in IPAQ questionnaire that was 3445.1±2150.8. The correlation between the two variables was 0.582 (p<0.001), and the correlation coefficient of EB versus accelerometer was 0.312 (p<0.001) and the IPAQ versus accelerometer data was 0.362 (p<0.001). **CONCLUSION:** Comparing the two different survey methods we found that all of them is appropriate to measure physical activity level, but they showed different mean values which need further investigation.

**ACKNOWLEDGMENTS**

The research was carried out with the support of the Human Resource Development Operational Program, EFOP-3.6.2-16-2017-00003: "Creating a Research Network for Recreational and Health Cooperation".

### P62 The effect of exercising on sexual functions in post-menopausal women

#### Annamaria Pakai, Andras Olah, Jozsef Betlehem

##### Faculty of Health Sciences, University of Pecs, Hungary

###### **Correspondence:** Annamaria Pakai (annamaria.pakai@etk.pte.hu)

Problems with the quality of life (QoL) could form quickly after menopause, which could become permanent after this period. **PURPOSE:** The aim of our study is to assess the symptoms originated from the lack of exercise and the presence of sexual dysfunctions among women in postmenopausal age. **METHODS**: Our cross-sectional, descriptive study was carried out in Pecs, Hungary between August 2017 and January 2018. Women aged 40-65 were selected, who reached post menopause, sexually active and agreed to participate in the study (N=102). During non-probability, convenience sampling we used questionnaires to collect data (FSFI, MENQOL, Godin-Shephard leisure). We calculated descriptive statistics, correlation and regression analysis, χ2test and independent t-test (p<0.05). Our results are presented with confidence intervals as well. **RESULTS:** Mean age was 50.56±4.35 years, 55.88% of women reached menopause. 47.06% of the respondents do sports actively, 47.06% of them showed sexual dysfunction according to FSFI results. FSFI results were higher among those who exercise regularly, and the presence of sexual dysfunctions were lower (p<0.05). Exercising regularly and occurrence of dysfunctions (p<0.001), alongside with the number of symptoms occurred and FSFI results showed significant differences (p<0.001). **CONCLUSION:** Increasing physical activity and reducing the number of symptoms helps to prevent the development of sexual dysfunctions. A study using a control group could point out more complex results on the effect of exercising in post-menopause.

### P63 Characterization of the physical activity of patients with heart failure based on actigraph and CRT device data

#### Csaba Melczer^1^, Laszlo Melczer^2^, Bence Laszlo Raposa^1^, Andras Olah^1^, Pongrac Acs^1^, Jozsef Betlehem^1^

##### ^1^Faculty of Health Sciences, University of Pecs, Hungary; ^2^Heart Institute, University of Pecs, Hungary

###### **Correspondence:** Csaba Melczer

The effect of regular physical activity on health is widely recognized. Several studies have proved its key importance for heart patients. However, patients may not be assuming adequate amounts of movement. The 6-minute walk test is used to check the physical status of the patients in the clinic. **PURPOSE:** This study aimed to estimate the 6-minute walk test result based on Actigraph and CRT device’s telemetry data. **METHODS:** Total of 42 patients suffering from heart disease were recruited from the Heart Clinic of Pécs into the study. Actigraph and built-in CRT device sets of values were used to describing physical performance. The Patient Activity % value was used to estimate the distance traveled over a 6-minute walk test. **RESULTS:** On further data analysis, we have concluded that the distance walked during the six-minute-long test may be measured by PA% from the data of the CRT device. **CONCLUSION:** With our method, the changes in patients' physical status could be monitored telemetrically with assistance from the implanted electronic device.

**ACKNOWLEDGMENTS**

The research was carried out with the support of the Human Resource Development Operational Program, EFOP-3.6.2-16-2017-00003: "Creating a Research Network for Recreational and Health Cooperation".

### P64 Effect of physical activity to economic activity

#### Csilla Filo, Pongrac Acs, Andras Olah, Jozsef Betlehem

##### Faculty of Health Sciences, University of Pecs, Hungary

###### **Correspondence:** Csilla Filo

More studies and research activities have analyzed the relationships between physical activity and economy activity in the past decades. Vitality is a basic prerequisite for labor market success. **PURPOSE:** The research action and study aimed to present the member of society sport and recreational activity effect on the situation of the labor market. In addition, look for the significant relationship between physical activity and economic activity. **METHODS**: On-line questionnaire was completed (n=651) on a regionally representative sample in South-Danubian Region. After processing the database, the hypotheses were verified by independent t-tests, correlation and Cramer’s analysis (p<0,05). **RESULTS:** More studies have revealed a relationship between physical activity and labor market activity. I based my research on this fact in the South-Transdanubian region. Confirmed that the long-term preservation of workers' health becomes an increasingly important priority. Physically active workers have shown better social and economic indicators than inactive staff. The effect of physical activity also increases in income levels. **CONCLUSION:** There appears to be, therefore, a link between physical activity and the structure of the labor market connected to initial access to employment and then higher income opportunities with aging that are associated with a career ladder.

**ACKNOWLEDGMENTS**

The research was carried out with the support of the Human Resource Development Operational Program, EFOP-3.6.2-16-2017-00003: "Creating a Research Network for Recreational and Health Cooperation".

### P65 Stressors and burnout in the diagnostic medical departments

#### D Sipos^1,2,3^, A Pandur^1^, A Kedves^2,3^, V Varga^1^, M Csima^2^, I Repa^1,3^, A Kovacs^1,2,3^, A Olah^1^, J Betlehem^1^

##### ^1^Doctoral School, Faculty of Health Sciences, University of Pecs, Hungary; ^2^Department of Medical Imaging, Faculty of Health Sciences, University of Pecs, Hungary; ^3^Somogy County Kaposi Moricz Teaching Hospital Dr. Jozsef Baka Diagnostic, Radiation Oncology, Research and Teaching Center, Kaposvar, Hungary

###### **Correspondence:** D Sipos

Burnout is common among health care professionals. It may have a negative impact on the personal, professional life also on the effectiveness of the healthcare system. **PURPOSE:** Our aim was to measure occupational burnout levels among radiology department workers in Hungary. **METHODS**: The Maslach Burnout Inventory (MBI) was used to measure burnout levels alongside our self-made questionnaire. All data were analyzed using SPSS V 24.0. Descriptive statistics, independent samples t-test and one-way ANOVA with Kruskal-Wallis analysis were performed to examine the relationship between given demographic characteristics and the three dimensions of burnout (p<0.05). **RESULTS:** 404 (n=404) radiology department workers participated in the survey. The sample had a high mean burnout score for emotional exhaustion (34,28; SD12,98) depersonalization (12,81; SD6,62) and personal achievement (41,03; SD8,70) compared to MBI norms. Age, modalities and years spent in the health care system had significant influence for all three dimensions of burnout (p<0.05). Educational level affected personal achievement significantly (p=0,001). **CONCLUSION:** According to our results a high number of radiology department workers are experiencing occupational burnout at emotional exhaustion and depersonalization dimension of burnout.

### P66 Circulating adrenal steroid levels in response to extreme physical stress in male athletes

#### Eva Csondor^1^, Gellert Karvaly^2^, Roland Ligetvari^1^, Gabriella Far^1^, Barna Vasarhelyi^2^, Viktor Toth Miklos^3^, Miklos Toth^1,2,4^, Timea Stromajer-Racz^1^, Jozsef Betlehem^1^, Andras Olah^1^, Pongrac Acs^1^

##### ^1^Faculty of Health Sciences, University of Pecs, Hungary; ^2^Semmelweis University, Budapest, Hungary; ^3^Eötvös Lóránd University, Budapest, Hungary; ^4^University of Physical Education, Budapest, Hungary; ^5^International Training Centre, Budapest, Hungary

###### **Correspondence:** Eva Csondor

Adrenal steroid molecules play a significant role in the regulation of cardiovascular, metabolic and other functions in response to acute stress. **PURPOSE:** To prove that as a result of stress the concentration of steroid hormones in the adrenal cortex increases in blood, probably in this way they help the body adapt. **METHODS**: We investigated the plasma levels of 14 different steroid molecules in a model of extreme acute physical and mental stress in male athletes. Steroid levels were measured with a combination of liquid chromatography-tandem mass spectrometry technique. All values were measured at baseline, at maximum stress situation and thirty minutes later. We used a paired t-test and descriptive statistic. **RESULTS:** Steroid metabolites were elevated only in the physical stress model. Nine steroid molecules were elevated in the restitution phase, and we recorded the changes of some steroid for the first time in this model. **CONCLUSION:** Steroid metabolites of the adrenal cortex clearly respond to stress. Mental stress did not have a significant effect on steroid levels. Clarification of the physiological roles of the changes in steroid levels needs further investigations.

### P67 Survey of trait aggression among athletes and non-athletes

#### Kata Morvay-Sey^1^, Eva Tekus^2^, Andras Olah ^1,^ Antal Kovacs^1^, Agnes Palvolgyi^1,^ Agnes Kerner^1^, Pongrac Acs^1^, Jozsef Betlehem^1^

##### ^1^Faculty of Health Sciences, University of Pecs, Hungary; ^2^Faculty of Sciences, University of Pecs, Hungary

###### **Correspondence:** Kata Morvay-Sey (kata.sey@etk.pte.hu)

**PURPOSE**: The aim of the study is to investigate the trait aggression of athletes (a) and non-athletes (na) with a questionnaire. **METHODS**: 14-18 years old respondents (N=280) filled the standardized Buss-Perry Aggression Questionnaire completed with other (demographic, sports habits) questions. Athlete group (n=140) participated regularly in training for at least 3 years. Athletes (n=140) were divided into subgroups: non-contact (aerobic n=20), sport games (handball n=20, basketball n=20), combat sport (wrestling n=20, judo n=20, karate n=20) and cyclic sport (n=20). **RESULTS**: Significant difference was found between athletes and non-athletes in anger (a:14.91±3.68, n.a.: 18.75 ±10.45); hostility (a:18.76±4.7, n.a.:22.36±12.89) physical aggression (a:18.08±5.39, n.a.:25.03±5.9, and trait aggression (a:65.75±12.88, n.a.:80.57±27.1). The non-athlete group had significantly (p≤0.05) higher trait aggression score (80.57±27.09) than the athletes divided into further subgroups according to the tactical division of sport (non-contact: 62.77±11.34; cyclic sport: 64.1±13.18; sports games: 63.42±14.78; combat sports: 68.13±12.46). If the sports examined separately, we found significant (p≤0.05) differences in the total score of trait-aggression between the non-athletes (80.57±27.09) paired with aerobics (62.77±11.34), basketball (61.46±10.41), judo (66.43±12.1) and swimming (64.1±13.18). **CONCLUSION**: Based on our results the positive effect of sport on trait aggression is visible.

**ACKNOWLEDGMENTS**

The research was carried out with the support of the HRDOP EFOP-3.6.2-16-2017-00003: "Creating a Research Network for Recreational and Health Cooperation".

### P68 Examination of the balancing ability and it’s subjective opinion reflected on body composition in patients with osteoporosis

#### Peter Tardi, Brigitta Szilagyi, Pongrac Acs, Marta Hock, Andras Olah, Jozsef Betlehem

##### Faculty of Health Sciences, University of Pecs, Hungary

###### **Correspondence:** Peter Tardi

**PURPOSE:** The number of falls/fractures in the postmenopausal population is a public health problem that can be caused by age-related body and physical changes. **METHODS**: Randomized sampling characterized our cross-sectional survey. The sample consisted of volunteer postmenopausal women (n=104). We examined body composition, static balancing ability, dynamic balancing ability, and activities-specific balance confidence. For statistical analysis, we used SPSS 20 program to characterize the sample, as well as dissimilarity tests (one-sample t-test) and correlation tests (Pearson correlation). **RESULTS:** Our sample (68.24±7.41years; 161.15±6.7cm; 71.73±12.69kg; 27.53±3.94kg/m2) bone density was -2.68±0.42 on the lumbar spine. High body fat percentage (40.8±6.84) was found. We found a significant (p=0.001) correlation between muscle mass and bone density. Significant (p<0.001) relationship was found between muscle and body fat percentage. We found no significant (p=0.55) relationship between activities-specific balance confidence and dynamic balancing ability (r=0.06). **CONCLUSION:** Based on international studies and the results of this study, we concluded that the bone density of the postmenopausal population is related to body composition parameters. Reduced muscle mass and balancing ability are typical, which is not reflected on the balance confidence.

**ACKNOWLEDGMENTS**

The research was carried out with the support of the Human Resource Development Operational Program, EFOP-3.6.2-16-2017-00003: "Creating a Research Network for Recreational and Health Cooperation".

### P69 The national economic burden of physical inactivation in Hungary

#### Pongrac Acs^1^, Miklos Stocker^2^, Jozsef Betlehem^1^, Andras Olah^1^, Antal Kovacs^1^, David Paar^1^, Mark Hoffbauer^1^

##### ^1^Faculty of Health Sciences, University of Pecs, Hungary; ^2^Corvinus University of Budapest, Hungary

###### **Correspondence:** Pongrac Acs (pongracz.acs@etk.pte.hu)

**PURPOSE:** The aim is to obtain reliable quantitative information on the effects of physical inactivity on the national economy (savings from reducing physical inactivity). **METHODS**: The research was to quantify the types and complications of diseases stemming from physical inactivity using data from the National Health Insurance Fund. Research conducted on this subject in Hungary and abroad confirms that the improvement of public health through physical activity is one of the most cost-effective tools. PAR indicator was used to give an estimate of the mortality or morbidity rate that occurred due to the risk factors identified in this study. **RESULTS:** The national economic burden of certain types of illnesses related to physical inactivity is 330 billion HUF (2017). The budgetary savings that can be made by a 10 percent reduction in inactivity level is 9,3 billion HUF. The research also touched on the impact of physical inactivity on sickness benefit expenses (1,7 billion HUF could be saved). **CONCLUSION:** Our research confirms that in Hungary, an attempt to increase physical activity can be considered a long-term investment (sports centers and health programs).

**ACKNOWLEDGMENTS**

The research was carried out with the support of the Human Resource Development Operational Program, EFOP-3.6.2-16-2017-00003: "Creating a Research Network for Recreational and Health Cooperation".

### P70 Increased levels of serum endothelin-1 in response to acute extreme physical stress with preserved left ventricular function in male Hungarian athletes

#### Roland Ligetvari^1^, Pongrac Acs^1^, Gabriella Far^1^, Eva Csondor^1^, Zsolt Komka^3,4^, Istvan Szokodi^1^, Miklos Viktor Toth^2^, Miklos Tooth^1,3,4^, Timea Stromajer-Racz^1^, Jozsef Betlehem^1^, Andras Olah^1^

##### ^1^University of Pecs, Hungary; ^2^Eotvos Lorand University, Budapest, Hungary; ^3^Semmelweis University, Budapest, Hungary; ^4^University of Physical Education, Budapest, Hungary

###### **Correspondence:** Roland Ligetvari

Endothelin-1 (ET-1) has not only a vasoconstrictor and positive inotropic effect but direct arrhythmogenic potency. Therefore, it is a strong candidate for playing a role in sudden cardiac death. **PURPOSE:** To characterize the ET-1 levels upon extreme physical and mental stress in professional handball players. **METHODS:** We investigated the response of 62 athletes (mean age=23) to extreme physical (vita maxima treadmill test) and mental stress (simulated military combat model). Circulating peptide levels were analyzed with ELISA systems. Circulatory, metabolic and gas exchange values were monitored. All values were measured at baseline, at maximum stress situation, and in the restitution phase. **RESULTS:** ET-1 levels were unchanged in mental stress but showed elevated values at the peak of physical stimulation returning to basal levels 30 minutes later (baseline: 5.5±3.7; peak: 6.82±4.6; recovery: 6.04±5.5 pg/ml). Subjects showed differential ET-1 answers in the physical stress model: 85% of the individuals showed elevated, whereas 15% showed lower levels after the treadmill test. **CONCLUSION:** Our observation of elevated ET-1 levels indicates that plasma ET-1 levels are worthy to monitor upon extreme physical load.

**ACKNOWLEDGMENTS**

Supported by GINOP-2.3.2-15-2016-00047, Szechenyi 2020.

### P71 Comparison of the global physical activity questionnaire results with accelerometer data among healthy young adults

#### Viktoria Premusz, Alexandra Makai, Jozsef Betlehem, Andras Olah, Csaba Melczer, Csilla Filo, Kinga Lampek, Pongrac Acs

##### Faculty of Health Sciences, University of Pecs, Hungary

###### **Correspondence:** Viktoria Premusz

General positive effects of regular physical activity (PA) on health conditions are well known. However self-reported data on mode, frequency, duration, and intensity of PA differs. **PURPOSE:** The aim of the study was to compare subjective and objectively measured physical activity (PA) patterns of young adults for a further population-based application. **METHODS**: A cross-sectional survey with convenience sampling was conducted during February-April of 2018 to examine the socio-demographic, anthropometric, and PA data (GPAQ, Actigraph GT3X) of 113 healthy students (age 21.5±1.8 years, BMI 22.0± 5.7 kg/m^2^) of the University of Pécs, Hungary. **RESULTS:** Respondents spent in average five hours (298.5±314.0 min/wk) with active transportation, almost two hours (111,5±126.5 min/wk) with moderate recreation weekly and 6.5 hours (397.7.46±186.4 min/day) with sitting daily according to the GPAQ database. Moderate to vigorous physical activity (MVPA) was 450.5±466.0 min/week with GPAQ, only 344.4±141.2 min/week with GT3X (p<0.001). We found a moderate correlation between GPAQ and accelerometer MVPA data them (R=0.403, p<0.001). **CONCLUSION:** Despite the fact, that objective measures show moderate correlation with accelerometer data, they are applicable to large population samples. Distressing inactivity of university student needs urgent interventions.

**ACKNOWLEDGMENTS**

The research was carried out with the support of the Human Resource Development Operational Program, EFOP-3.6.2-16-2017-00003: "Creating a Research Network for Recreational and Health Cooperation".

### P72 Examination of work-related stress and coping strategies among ground- and air-ambulance workers

#### Bence Schiszler, Balint Banfai, Attila Pandur, Balazs Toth, Henrietta Csonka, Jozsef Betlehem, Balazs Radnai

##### Institute of Emergency Care and Pedagogy of Health Faculty of Health Sciences, University of Pecs, Hungary

###### **Correspondence:** Bence Schiszler (schiszlerbence89@gmail.com)

**PURPOSE:** Based on the scientific literature, a high level of stress is present among Hungarian health sector workers, which can affect the individuals' life. This study aims to discover major risk factors and its extent of work-related stress among ground- and air ambulance personnel and to identify their positive and negative coping strategies. **METHODS**: a national survey was conducted among Hungarian rescue workers between June and October 2015. Data were collected with a self-designed questionnaire using Holmes and Rahe (2000) Stress and coping validated short questionnaire online form. A total of 141 people took part in the survey. Data were analyzed with MS Office Excel, SPSS 20.0 programs, descriptive statistical analysis, Chi-square test, two-sample T-test. **RESULTS:** Among the ground rescue workers increased work-related stress effects are detectable (p <0.01), and they are exposed to a much greater variety of physical and psychological symptoms (p <0.05). Based on Global Stress and Coping Index more effective coping mechanisms can be observed among air rescue workers (p <0.01). **CONCLUSION:** It is important to provide regular professional theoretical and practical training for strengthening coping strategies among ambulance workers. Occupational stress reduction needs to be an essential part of the job of human resource management.

### P73 Energy demands of top-level Croatian aesthetic sports athletes: a case study

#### Marijo Moznik, Tamara Despot, Stipo Dajakovic

##### Faculty of Kinesiology, University of Zagreb, Croatia

###### **Correspondence:** Stipo Dajakovic (stipo.dajakovic@kif.hr)

Being an athlete in aesthetic sport does not just mean the beauty of movement, technical perfection, and choreographic routine, but also great energy consumption and thus the development of all energy capacities. **PURPOSE:** In order to adequately develop the aesthetic athletes’ energy capacities, it is necessary to set the criteria for the evaluation of the parameters themselves. **METHODS:** The subject sample consisted of two best Croatian male aesthetic sports athletes (dancesport and artistic gymnastics) with recognized results in European and World Championships in the last five years of competing. They performed an incremental treadmill test for estimating aerobic capacity. Variables that are measured was peak velocity attained in test (F1vmax), the velocity at anaerobic threshold (F1vVT), maximum heart rate attained (F1HRmax), heart rate at anaerobic threshold (F1HRVT) and maximum oxygen uptake (F1VO2; F1RVO2). **RESULTS:** Dancesport athlete (F1vmax 17.5 km/h; F1vVT 14.5 km/h; F1HRmax 183 bmp/min; F1HRVT 168 bmp/min; F1VO2 4.27 lO_2_/min; F1RVO2 54.78 mlO_2_/kg/min); Artistic gymnastics athlete (F1vmax 17 km/h; F1vVT 12.5 km/h; F1HRmax 187 bmp/min; F1HRVT 169 bmp/min; F1VO2 4.57 lO_2_/min; F1RVO2 56.9 mlO_2_/kg/min) **CONCLUSION:** Top-level dancers and artistic gymnasts differ from average population according to the best technical performance, which can be shown exclusively if the energy capacities are on a top level. These two subjects can be models in the development of new dancers and gymnasts in Croatia.

### P74 Use of technology in physical education classes - a perspective of PE teachers

#### Ilija Klincarov^1^, Biljana Popeska^2^, Orce Mitevski^1^, Goran Nikovski^1^, Katerina Mitevska Petrusheva^3^

##### ^1^Faculty of Physical Education, Sport and Health, Ss. Cyril and Methodius University, Skopje, Macedonia; ^2^Faculty of Educational Sciences, Goce Delcev University, Stip, Macedonia; ^3^Faculty of Education, International Balkan University, Skopje, Macedonia

###### **Correspondence:** Ilija Klincarov

Technology implemented in PE teaching process can increase the level of PA, motivates children to move and could facilitate the process of teaching and learning. **PURPOSE:** To determine the attitudes of PE teachers regarding the implementation of technology in PE teaching process as well as to explore different approaches in its application. **METHODS**: A total of 40 PE specialist teachers from 15 different schools in Macedonia were interviewed using a specially designed questionnaire. **RESULTS:** PE teachers share the opinion that technology in general decreases the level of PA (64%), children are not interested to use it at PE classes (52%) but it is well implemented in PE classes, it could motivate children to be more active (39%). PE teachers mainly use technology as personal support in preparation for classes (60%) and during the classes when demonstrating new skills. Youtube videos and different mobile applications are the most applied forms. **CONCLUSION:** Technology-supported teaching and learning could be very effective and motivating for students. Balanced and well-planned use of technology at PE classes could increase children interest in participation.

### P75 Academic motivation of students in physical education and sport at Sofia University “St. Kliment Ohridski”

#### Georgi Ignatov^1^, Iliana Petkova^2^

##### ^1^Sport Department, Sofia University “St. Kliment Ohridski”, Macedonia; ^2^Faculty of Education, Sofia University “St. Kliment Ohridski”, Macedonia

###### **Correspondence:** Georgi Ignatov

The quality teaching process depends not only on the teacher but also on the academic motivation of students participating as equal partners in the educational process. The **PURPOSE** of this article is to represent the results from a study aimed at establishing the degree of academic motivation of students. Totally 45 participants, students at the second and third year of bachelor study in specialty Physical Education and Sport at Sofia University participated in the study. **METHODS:** Angel Velichkov's questionnaire was used to evaluate the level of academic motivation in which are set the factors that favor or impede the formation of high academic motivation and allows to trace its development. **RESULTS:** Results are aimed to prove the assumptions that students in Physical Education and Sports specialty should have: 1) active attitude towards the learning process; 2) internal self-discipline and 3) striving to complement and extend the knowledge obtained. The analysis will be comparative, based on the gender and year of study which will allow us to determine whether these two criteria also influence the degree of academic motivation. **CONCLUSION:** The establishment of differences in the levels of these three criteria and overall level of the students' academic motivation will enable teachers to optimize the teaching process and make it more personal-oriented.

### P76 Fundamental locomotor movements in elementary school children in urban areas in Skopje

#### Aleksandar Aceski, Ilija Klincarov, Martin Andonovski, Jelena Aleksic

##### Faculty of Physical Education, Sport and Health, Ss. Cyril and Methodius University, Skopje, Macedonia

###### **Correspondence:** Aleksandar Aceski

Regular physical activity in children is important for overall child development including all developmental aspects: motor, cognitive and socio-emotional. Through movements, children explore and learn about their environment. This process of moving and learning enhance children cognitive functions and their cognitive development. Early childhood is a period when fundamental motor movements start to manifest. These movements are the essential building blocks and a precondition for learning other complex and specialized movements in different sports. Fundamental movements are also the main and essential part of all physical education curricula for children from an early age. **PURPOSE:** The main purpose of this study is to estimate the level of acquisition of locomotor movement manifested in elementary school children from the urban area of city Skopje, Republic of Macedonia. **METHOD:** Children`s motor skills were estimated using the protocol “Test of Gross Motor Development (TGMD2)”. **RESULTS:** Obtained results suggest no differences in total scores for all estimated locomotor movements as well as differences in personal scores for each of the analyzed skills regarded the age and gender of children. **CONCLUSION:** Although the study was done on a small and selected sample, obtained results could be used as a good foundation for designing PE curricula that will be based on real and scientifically proven results.

### P77 Who can be reached in media-based physical activity promotion campaigns? Preliminary study

#### Danijel Jurakic^1^, Zrinka Greblo Jurakic^2^

##### ^1^Faculty of Kinesiology, University of Zagreb, Croatia; ^2^University Department of Croatian Studies, University of Zagreb, Croatia

###### **Correspondence:** Danijel Jurakic (danijel.jurakic@kif.hr)

TV program with the aim to promote physical activity and healthy eating was created and broadcasted on Croatian national television. A total number of 16 episodes were aired once per week from October 2016 to January 2017. Each episode was dedicated to a single recreational sport or recreational exercise presented from the view of exercise beginner, exercise expert, nutritional expert and personal story of an experienced exerciser. **PURPOSE:** To determine the characteristics of the audience reached by the above-mentioned TV program. **METHODS**: Descriptive analysis was conducted using following parameters: AMR (average minute rating) which represents an average number of viewers per minute, AMR% (the average of the audience per minute divided by the total of a population). **RESULTS:** The average AMR of all episodes was 62.294 viewers i.e. 1.54% of the whole population. TV program attracted more females (AMR 37.416, AMR% 1.76) than males (AMR 26.651, AMR% 1.30). According to income, most of the viewers are categorized as "middle income" (AMR 35.088, AMR% 1.48) and least as "higher income" (AMR 3.719, AMR% 1.18). Majority of the viewers belonged to middle-aged group 55-59 (AMR 8.593, AMR% 2.77) as opposed to young adults 35-39 (AMR 2.542, AMR% 0.90). **CONCLUSION:** TV program oriented toward the promotion of physical activity and healthy eating has the potential to engage 1.5% of the population, particularly middle-aged women with middle-income.

### P78 Frequency foot deformity in the first-grade primary

#### Ivan Vasiljevic, Jovan Gardasevic

##### Faculty for Sport and Physical Education, University of Montenegro, Montenegro

###### **Correspondence:** Ivan Vasiljevic (vasiljevic.ivan301@gmail.com)

To determine the moment of occurrence of postural disorders, regardless of the cause of its creation, this problem must be examined from the moment of entry of children into the new environment, i.e. in kindergarten or school. In developed countries, there are institutes for preventive work with children that are equipped with modern diagnostic instruments whose task is to timely detect deformities and developing therapeutic and preventive programs. **PURPOSE**: Estimate possible foot deformities in elementary school students. **METHODS**: The survey was conducted in two primary schools in Niksic in Montenegro, with a sample of 85 students. To determine the status of the foot is applied on the basis of the orthopedic method that looks at the plantar side of the foot. **RESULTS**: Mentioned research indicated very worrying data, where over 70% of children who were tested have a certain deformation of the foot in the said level. **CONCLUSION**: Prevention and treatment should begin as early as possible in order to enforce complex exercises to increase the elasticity of the foot and plantar extensor muscle strength.

### P79 The effects of 14-day bed rest and recovery on skeletal muscle in younger and older subjects

#### Katja Koren^1^, Bostjan Simunic^1^, Stefano Lazzer^2^, Enrico Rejc^3^, Rado Pisot^1^

##### ^1^Science and Research Centre of Koper, Slovenia; ^2^Department of Medicine, University of Udine, Italy; ^3^Kentucky Spinal Cord Injury Research Center, University of Louisville, USA

###### **Correspondence:** Katja Koren (Sasa.Pisot@zrs-kp.si)

Bed rest is a recognized ground-based model to study the effects of physical inactivity and microgravity. **PURPOSE:** To evaluate the effects of 14-day bed rest and recovery on skeletal muscle architecture, force, and power in young and older. **METHODS**: Sixteen older and seven younger male subjects were exposed to 14-day horizontal bed rest, followed by rehabilitation. Before (BDC) and after (BR14) the bed rest and after 14th (R+14) day of rehabilitation we measured vastus lateralis (VL) architecture, knee extensors maximal voluntary isometric contraction (MVC) and maximal jumping explosive power (EP). **RESULTS:** VL architecture, MVC and EP reduced at BR14 only in older. The thickness and pennation angle decreased at BR14 for -6% (P=.050) and -13% (P=.001), respectively, and remained reduced even at R+14. Similarly, MVC and EP decreased for -13% (P=.001) and -15% (P=.001), respectively, and remained decreased at R+21. **CONCLUSION:** Our study demonstrated more pronounced responses to bed rest in older. Several important muscle parameters did not recover in older that has an important clinical value to avoid prolong bed rest and/or to develop interventions. Registered at ClinicalTrials.gov (NCT02694471).

**ACKNOWLEDGMENTS**

Co-financed under the Cross-border Cooperation Program Slovenia-Italy 2007-2013 by the European Regional Development Fund and national funds.

### P80 Reducing physical activity and screen time behaviors among children and young adolescents in Kazakhstan

#### Shynar Abdrakhmanova^1^, Kwok Ng^2,3^, and Assel Adayeva^1^

##### ^1^National Center of Public Health, Almaty, Kazakhstan; ^2^Department of Educational Sciences and Psychology, University of Eastern Finland, Finland; ^3^Department of Physical Education and Sport Sciences, University of Limerick, Ireland

###### **Correspondence:** Kwok Ng (kwok.ng@hbsc.org)

**PURPOSE**: To examine the associations between PA and screen time behaviors (STB) among children and young adolescents in Kazakhstan. **METHODS**: Data were pooled from the 2015/16 Childhood Obesity Surveillance Initiative (COSI) study (n=4932, 49.8% girls, Mean_age_=8.77 SD_age_=0.68) and the 2017/18 Health Behavior in School-aged Children (HBSC) study (n=4153, 49.3% girls, Mean _age_=12.93 SD_age_=1.64). Parental proxy reporting of children's PA levels and weekday STB time in the COSI study. In the HBSC study, young adolescents self-reported their PA levels in the past week and the number of STB hours including TV viewing during weekdays. **RESULTS**: The number of children and young adolescents who met the PA recommendations reduced among children aged 9y (68.1%), to young adolescents aged 11y (35%), 13y (37%) and 15y (31%). Similarly, there was a reduction in the proportion of adolescents who met the STB recommendations between the ages of 9y (75%), 11y (60%), 13y (53%), and 15y (47%). **CONCLUSION**: Despite some limitations in the methodologies between the COSI and HBSC surveys, and self-reporting of behaviors, there was a pattern of lower health-promoting activities as children transition into young adolescents.

### P81 The effect of 15-minute active recess on children’s physical activity

#### Tadeja Volmut (Sasa.Pisot@zrs-kp.si)

##### Department for School Education, Faculty of Education, University of Primorska, Koper, Slovenia

Physical education lessons and the recess represent two main contexts in which all children have the opportunity to be physically active. **PURPOSE:** The purpose of the study was to determine the contribution of free play and semi-structured 15-minute active recess to children’s physical activity levels. **METHODS**: The sample included 92 children (47 boys), aged 6 to 8 years. Children were selected in the control (CG; 32 children) and the experimental group (EG; 60 children). EG have available sports equipment and have to spend recess outdoors, whereas the control group didn't. Physical activity was measured with accelerometer (ActiGraph, Pensacola, Florida) during a single day. The accelerometer was placed around the child's waist (right hip) with an elastic belt. The children wore the accelerometers all the time except when sleeping, swimming or taking a shower or bath. We carefully recorded the time of the active recess. **RESULTS:** The recess period lasted 14.2 ± 2.5 minutes. The experimental group was more active during 15-minute recess for 336 % (P=0.001) and that was reflected also in higher daily PA for 8 % (P=0.064). The experimental group achieved also higher time spent in the moderate-to-vigorous intensity of physical activity, being 84.7 ± 27.5 minutes in the experimental group and 73.3 ± 23.8 in the control group (P=0.025). **CONCLUSION:** The study shows the importance of active recess on achieving daily physical activity levels.

### P82 The relationship between physical activity and quality of life among adolescents and orphans

#### Lucija Maglica^1^, Gordan Drasinac^2^, Ana Penjak,^1^ Hrvoje Karnincic^1^

##### ^1^Faculty of Kinesiology, University of Split, Croatia; ^2^University of Split, Croatia

###### **Correspondence:** Lucija Maglica

**PURPOSE**: To determine the relationship between physical activity (PA) and the quality of life in secondary school pupils and in orphans. The second aim was to determine differences in PA habits among groups. **METHODS**: 91 participants (age 15.4±1.2) divided into two subsamples: secondary school pupils (n=75) and orphans from the Split region (n=16). The variable sample consisted of nine variables out of which four variables that questioned the quality of life by means of the WHO Quality of Life questionnaire and five variables that questioned subjective assessment of weekly PA by means of The International Physical Activity Questionnaire. Differences between groups were tested by the Mann-Whitney U Test. Correlation among the quality of life self-assessment variable and weekly PA habits on total sample and for both groups separately was established by Spearman coefficient rank. **RESULTS**: Differences between groups were found in the variable of light PA intensity level (U=376.5; Z=-2,3; p=0.02). Housekeeping activity in orphan’s institution is performed by employees, not by the children themselves. Intensive PA, among orphans it significantly correlates with social aspect (ρ=0.58) and surroundings aspect of quality of life (ρ=0.62). **CONCLUSIONS**: Orphans share a similar amount of PA with the other pupils but sport for them has a particular meaning. The more sports they do, the more satisfied-regarding the social domain of quality of life-they feel.

### P83 Psychological characteristics of senior female volleyball players

#### Milena Aljinovic, Nika Stanovic, Mirjana Milic

##### Faculty of Kinesiology, University of Split, Croatia

###### **Correspondence:** Milena Aljinovic

**PURPOSE:** The aim of this study was to determine the psychological characteristics of senior female volleyball players, as well as potential differences regarding their chronological age, playing experience and player position. **METHODS**: The subject sample included 62 senior Croatian female volleyball players (average chronological age 18.29±3.37 years), competing in the Premier Croatian volleyball league, divided into 5 subgroups according to their player position in: technicians (22.58%), diagonal players (9.68%) receivers-hitters (30.65%), middle blockers (20.97%) and libero players (16.13%).The sample of variables was represented by PSIS-Y and CSAI-2 questionnaire variables, in total nine psychological dimensions were used: *mental preparation*, *motivation*, *concentration*, *self-confidence*, *relation with the team* and *anxiety control*, as well as two dimensions of *anxiety* state (*cognitive* and *somatic component*) and *self-confidence*. **RESULTS:** Metric characteristics of the validated PSIS-Y and CSAI-2 questionnaire scales had high values, confirming the high quality of the measuring instruments used in measuring psychological characteristics of volleyball players. Also, previous findings regarding the psychological functioning of senior female volleyball players were additionally confirmed. Significant differences were determined between groups of players from different clubs in three psychological characteristics defining anxiety (cognitive and somatic anxiety and PSIS-Y questionnaire anxiety scale). **CONCLUSION:** The obtained results indirectly show that psychological characteristics are equally important in playing all the positions in volleyball and that Croatian volleyball teams do not pay enough attention to the perfection of these skills.

### P84 Doping factors and correlates of potential doping behavior in team sports coaches: a gender-specific analysis

#### Dora Maric^1,2^, Sime Versic^2^, Antonino Bianco^1^

##### ^1^SPPF Department, University of Palermo, Palermo, Italy; ^2^Faculty of Kinesiology, University of Split, Croatia

###### **Correspondence:** Dora Maric (do.maric@gmail.com)

Coaches are found to be the most influential persons in athletes' behavior. Studies reported that athletes perceived them as a credible source of information, and have high trust in their opinion regarding doping. **PURPOSE**: To determine doping attitudes (positive vs. negative opinion about potential doping behavior in athletes [PDB]), and factors influencing PDB in sports coaches involved in team sports. **METHODS**: 113 coaches (16 females; age: 41.5±5.1 years) involved in four team sports (soccer, basketball, volleyball, and handball). Variables were collected by previously validated questionnaires, which included questions on sociodemographics, sport-factors, and doping-related factors. **RESULTS**: There was a significant difference between sports in coaches' opinion about doping presence in their sport (KW: 10.44, p < 0.05). No significant difference was found between genders in opinion about doping presence in their sport (KW: 2.79, p = 0.09), and PDB (KW: 6.81, p = 0.08) while female coaches are more oriented toward more rigid penalties for doping offenders than males (KW: 11.01, p = 0.02). Logistic regression identified coaches’ opinion about doping presence in their sport as predictive for PDB (OR: 1.45, 95%CI: 1.31-2.01). **CONCLUSION**: Coaches strong impact on athletes’ attitudes and behaviors makes them important agents in the fight against doping.

### P85 Aikido in Croatian society: a case study

#### Ana Sabolic, Suncica Bartoluci

##### Faculty of Kinesiology, University of Zagreb, Croatia

###### **Correspondence:** Ana Sabolic

Aikido is a contemporary Japanese martial art whose popularity in Croatian society has been increasing for past years. Since its arrival to the West in the 1950s, until today, aikido has been modified and its original principles and ideas have been interpreted and applied differently depending on the school, tradition and wider socio-political context. Today, unlike its original militaristic orientation, aikido is focused on achieving physical well-being and psychophysical development of an individual, but basic orientation differs from school to school. **PURPOSE**: To determine the role and meaning of aikido in Croatian society on the case of the Varaždin Aikido club where Aikikai style of aikido is practiced. **METHODS**: This paper concerns a six months period of ethnographic research. We also made 10 semi-structured interviews with trainees and teachers. **RESULTS**: There is a very close connection between Eastern culture and a specific way of life bonded with practicing aikido and martial arts in general, but in Croatian society, these are "modified". **CONCLUSION**: Due to different cultures and lifestyles and the distance from its source, the modern way of life and the Western aspiration for competing, the heart of aikido is being changed, simplified, and put in frames and shapes that are closer and more similar to the customs and culture of Croatian society.

### P86 Evaluation of active breaks in toddlers group

#### Ivan Holik (ivholik1@gmail.com)

##### Faculty of Kinesiology, University of Zagreb, Croatia

Active breaks can be used with toddlers and they are an excellent example of physical activity in the institution of early and pre-school education. **PURPOSE:** To determine the effectiveness and efficiency of performing 25 different physical activity breaks with toddlers. **METHODS:** 33 toddlers (aged 1.8 to 3 years) participated in this trial. Activities were recorded with a video camera and the observation method was used for the qualitative analysis results. A sample of qualitative variables was created based on the observation of toddlers while performing various types of motor content. Motor contents were analyzed by the following components that make the variables: feasibility, interest, quality of performance, involvement, usability, duration and efficiency in relation to the goal. Variables were rated: BAD (less than 50% of toddlers), GOOD (more than 50% of toddlers, but not all) or EXCELLENT (all or almost all), depending on the estimate. **RESULTS:** Stories which included physical activity, kinesiological elementary games, and multimedia content showed very good and optimal contents for performing physically active breaks with toddlers, while general preparatory exercises proved to be the worst accepted exercises. **CONCLUSION:** Physically active breaks can be applied in the toddlers’ group and they represent an interactive way to engage early age children in physical activity. The content must be appropriate to the age of the children, their possibilities and interests, and their implementation is influenced by the professional competences of the educators.

### P87 Reliability of the Croatian version of the Ryff´s Psychological Wellbeing (PWB) scale

#### Maja Ban^1^, V Mijoc^2^, T Fehervari^3^

##### ^1^Allegra Fitness & Wellness Centar, Zagreb, Croatia; ^2^Catholic University of Croatia, Zagreb, Croatia; ^3^Karlovac University of Applied Sciences, Croatia

###### **Correspondence:** Maja Ban (maja.ban@student.kif.hr)

Psychological well-being is a multidimensional construct that consists of six different aspects of human actualization. Each dimension is different challenges that an individual face in the process of developing a person. **PURPOSE**: Overviewing the literature, no previous study reported data on the reliability of the Croatian version of Ryff's Psychological Wellbeing scale. The aim of the study was to determine the test-retest reliability of the Croatian version of Ryff's Psychological Wellbeing scale. **METHODS**: The sample consisted of 50 students of the University of Karlovac (52% men, 48% woman; mean age 19.38 years; SD 1.827;). The study was conducted on two occasions, and there were two weeks between two measurements. The 42-item Psychological Wellbeing (PWB) Scale was used in the study. **RESULTS**: Data analyze reported Spearman's rank correlation coefficient between test and retest data. The correlation between the first and the second measurements is statistically significant (Rho = 598; p <0.01). According to the internal reliability coefficient (Cronbach´s Alpha), results in the first measurement, i.e. Alpha is, 521, and in the second is 842. **CONCLUSION**: The results of the Croatian version of Ryff’s Psychological Wellbeing scale showed satisfactory reliability for measuring six aspects of wellbeing and happiness.

### P88 Reliability of the Croatian version of the sedentary behavior questionnaire

#### Vanja Blazun^1^, Vesna Mijoc^2^, Tamara Fehervari^3^

##### ^1^OVB Allfinanz Croatia, Zagreb, Croatia; ^2^Catholic University of Croatia, Zagreb, Croatia; ^3^Karlovac University of Applied Sciences, Croatia

###### **Correspondence:** Vanja Blazun (vanja_blazun@hotmail.com)

The Sedentary Behavior Questionnaire (SBQ) was designed to assess the amount of time spent doing 9 behaviors (watching television, playing computer games, sitting while listening to music, talking on the phone, doing paperwork/office work, sitting/reading, playing a musical instrument, doing arts, sitting and driving/riding in a car/bus/ train). The 9 items were completed separately for weekdays and weekend. **PURPOSE**: Overviewing the literature, no previous study reported data on the reliability of the Croatian version of the SBQ. The aim of this study was to determine the test-retest reliability of the Croatian version of SBQ on students. **METHODS**: The sample consisted of 53 students of University of Karlovac (52% men, 48% woman; mean age 19.38 years, SD 1.827). The study was conducted on two occasions. There were two weeks between measurements. Two scales were measured, one scale was the measure for the weekend and the other for the week. The Croatian version of Sedentary Behavior Questionnaire was used in the study. **RESULTS**: The correlation between first and second measurements for subscale week reported with Spearman’s rank correlation coefficient is statistically significant (Rho=.577; p<0.05). The correlation between first and second measurement for subscale weekend is statistically significant (Rho=.719; p<0.05). **CONCLUSION**: The results of the Croatian version of SBQ showed satisfactory reliability for measuring sedentary behavior during week and weekend on student population.

### P89 Differences in physical activities of two high school programs

#### Janja Ricov^1^, Franjo Rozijan^2^

##### ^1^Zagreb Sports Association, Croatia; ^2^Secondary School Zabok, Croatia

###### **Correspondence:** Janja Ricov (janja.ricov@student.kif.hr)

Development of modern technologies, lifestyle habits, especially of younger, have been significantly changed, resulting in spending their free time, consisting primarily of sitting-position activities. **PURPOSE**: Therefore, the goal of this research was to find the existence of significant differences in the level and intensity type of PA of High School students per various categories of PA. **METHODS**: Primary data for this research was collected on the bases of international IPAQ questioner for evaluation of daily activities. Questions were related to 7 days with the following activities: Free-leisure time, Household chores and Work and communication to work. The research was conducted on the group of 24 boys-computer technicians of Vocational-School and 25 girls-students of classic High-School in the city of Zabok. For the establishment of differences between student groups PA, non-parametric "Mann-Whitney U-test" was utilized. **RESULTS**: From this research, it could be concluded, that, there were statistically significant differences in PA between males and females, obtained in the following variables: Extremely difficult: days per week, hours and, minutes per day; Moderately difficult: days in the week; Activities in sitting position: hours-daily, minutes-daily. **CONCLUSION**: In all variables with significant differences among gender participants, higher averages were registered for boys. That means that boys have higher PA than girls. But boys also spend more time in sitting position-activities, then girls, while, in all other PA there are no significant differences among participating groups (Asymp.Sig>5%).

### P90 Differences in breathing patterns of male and female 400m runners

#### Marina Banovic^1,2^, Vlatko Vucetic^2^

##### ^1^Department of Anesthesiology, Resuscitation and Intensive care, Clinical Hospital “Sveti Duh”, Zagreb, Croatia; ^2^Faculty of Kinesiology, University of Zagreb, Croatia

###### **Correspondence:** Marina Banovic (marinabanovic11@gmail.com)

Breathing economics is important in sports in order to obtain the least possible energy consumption with optimal gas exchange. **PURPOSE:** To determine the differences among male and female athletes breathing patterns at maximum load. **METHODS:** 28 healthy Croatian 400m runners (14 M and 14 W) of similar age (mean M 19.96+/-3.60 vs W 19.13+/-3.20 years, p=0.53) conducted spiroergometric testing. **RESULTS:** There was no difference concerning breathing frequency, Rfmax (mean M 62.30+/-8.27 vs W 58.23+/-9.08/min, p=0.23). Men achieved higher values ​​in maximum minute ventilation, VEmax (mean M 161.34+/-24.70 vs W 104.67+/-13.72L, p<0.01) and one breath volume, VTmax (mean M 2.70+/-0.31 vs W 1.93+/-0.35L, p<0.01). A new variable, %VT/FVC, showing the percentage of their fortified vital capacity athletes breathe at maximum load, showed no significant difference among groups (mean M 44.01+/-4.08 vs W 47.69+/-7.49%, p=0.12). Correlation coefficient 0.48 among VEmax and Rfmax pointed that minute ventilation did not increase only by increasing frequency but also with breathing depth. A negative correlation exists between %VT/ FVC and Rfmax. **CONCLUSION:** Establishing better control of breathing frequency and depth at maximum effort could result in higher values ​​of %VT/FVC and greater breathing economics. Further research is needed to determine whether breathing training should become a regular part of the training process with the aim of achieving the optimal breathing pattern.

### P91 Comparative analysis of motor and functional abilities of athletes of different sport directions

#### Marina Vukotic (marina.vukotic82@gmail.com)

##### Faculty for Sport and Physical Education, University of Montenegro, Montenegro

**PURPOSE**: The main goal of this research is to determine if there are statistically significant differences in the motor and functional abilities in football, basketball and volleyball players, or to determine differences in quantitative and qualitative changes in motor and functional abilities in these athletes. **METHODS**: In accordance with the set-up goal, a transversal study was conducted in which the empirical and statistical methods were applied, and the research technique is testing. The research was conducted on a sample of 75 subjects, male sex divided into three subsamples, with an age range of 13 to 15 years. The selected variables in this study hypothetically cover the space of motor skills (9) and functional abilities (4). An analysis of ANOVA variance, multivariate analysis of MANOVA variance, LSD-test between all combinations and discriminatory analysis between all three subsamples were used to determine differences within groups. **RESULTS**: A statistically significant canonical discriminatory factor with a characteristic root of 0.24. According to the values of the centroid of the groups, a statistically significant difference within the groups was observed on the discriminator function. **CONCLUSION**: Based on the obtained results, it can be concluded that there are statistically significant differences in motor and functional abilities in athletes. Trainers can use this research in order to achieve better results with athletes.

### P92 *In Silico* evaluation of ADMET features of silybin and its derivatives by chemometric tools

#### Vanja Seregelj, Strahinja Kovacevic, Milica Karadzic Banjac, Sanja Podunavac-Kuzmanovic, Lidija Jevric

##### Faculty of Technology, University of Novi Sad, Serbia

###### **Correspondence:** Vanja Seregelj (vanjaseregelj@tf.uns.ac.rs)

Silybin or silibinin is a major active constituent of silymarin. It is used as a liver-protective drug in the form of supplements. Its short half-life in the organism and limited absorption is one of the main problems that restrict its effectiveness, so structural modifications and synthesis of new silybin-based derivatives are welcome. **PURPOSE**: To evaluate the ADMET (absorption, distribution, metabolism, excretion, and toxicity) features of new silybin derivatives compared to the features of silybin and to reveal potential similarities among them based on ADMET properties. **METHODS**: Seven compounds, available in the PDB database, were analyzed by vNN and PreADMET software, including silybin, quercetin, 2,3-dehydrosilybin, and four silybin glycoconjugates. ADMET features (liver toxicity, enzyme inhibition, membrane transporters inhibition, etc.) were predicted by using the models based on a restricted applicability domain. Chemometric tools were used in classification analysis for the detection of similarities among the studied compounds applying Statistica software. **RESULTS**: The results show that some of the newly synthesized silybin derivatives have favorable ADMET characteristics and low toxicity and some of the studied compounds are CYP inhibitors with binding affinity to membrane transporters (P-gp inhibitors or substrates). **CONCLUSION**: The studied compounds could be considered a good basis for further investigations of their hepatoprotective effects due to favorable ADMET features, and afterward their application in the treatment of liver diseases could be eventually considered.

### P93 Physical activity and quality of life in students aged 19 to 22

#### Nada Arseni, Hans Eric Reitmayer

##### Faculty of Physical Education and Sport, University Babes Bolyai, Cluj Napoca, Romania

###### **Correspondence:** Nada Arseni (arseni.nada@e-uvt.ro)

Nowadays due to the process of industrialization and the excessive technologization modern man leads an increasingly motionless life. With technology being more accessible, actions that required physical activity are now only a click away. In the search to make our life easier, we often neglect the benefits of physical activity to our health and to our wellbeing. **PURPOSE**: To examine the association between physical activity and quality of life in students aged between 19 and 22 years old. **METHODS**: Data were collected via an anonymous questionnaire from N=400 (200 female and 200 male) undergraduate students. Physical activity was assessed with the Occupational sitting and physical activity questionnaire (OSPAQ), and quality of life was assessed by the Questionnaire on Quality of Life CEEX 71/2006. **RESULTS**: Most of the students tend to have a sedentary lifestyle. Only a small percentage of them are involved in activities that are physically demanding. The quality of life is directly proportional to physical activity and does not depend on BMI. **CONCLUSION**: The physical activity level among university students is medium to low. Female students have a lower percentage of physical activity than male students. All of them should be encouraged to do more activities which involve movement during their spare time to increase their levels of physical activity and the quality of life.

### P94 Report of individual changes in anthropological status after finishing Croatian long-distance trail

#### Jere Gulin, Vlatko Vucetic, Stipo Dajakovic

##### Faculty of Kinesiology, University of Zagreb, Croatia

###### **Correspondence:** Jere Gulin (jere.gulin@kif.hr)

**PURPOSE**: Croatian long-distance trail (CLDT) is a first ultra-long hiking trail in Croatia which was created and finished for the first time in 2018. A subject of this research was one of the founders, and the first person to complete the route in a whole. Level of subject's aerobic endurance, as well as basic morphological parameters before and after the CLDT, are reported. **CASE PRESENTATION**: Subject, a 38 years old male, had started with the trail in April of 2018 and finished in the August of 2018. There was a total of 103 days on the trail. The total distance that was covered is 2281 km. His body weight was reduced by around 8 % (Initial: 70.5 kg, Final 65.0 kg), his body fat was reduced by 21% (I: 16.8%, F: 13.2%). Level of his relative VO2max, measured by progressive incremental walking test on a treadmill was increased by around 15% (I: 44.15 mlO2/kg/min, F: 50.83 mlO2/kg/min). His maximal incline on a treadmill test was increased by 18% (I: 20°, F: 24°) **CONCLUSION**: Significant improvements in the overall level of fitness was noted. Subject reported no injuries during the hike which had also attributed to these results. Long distance hiking can cause significant changes and improvements in aerobic energetic capacities and morphological characteristics.

**CONSENT**

Written informed consent was obtained from the participant for publication of this case report. A copy of the written consent is available for review from the Editors of this journal supplement issue.

### P95 Effects of two types of volleyball training programs on the development of motor skills

#### Mateja Deranja, Josip Deranja

##### Faculty of Kinesiology, University of Zagreb, Croatia

###### **Correspondence:** Mateja Deranja (mateja.krmpotic@gmail.com)

Motor development largely depends on the variety of stimuli which have effects on the development of motor skills. Nowadays, girls are involved very early in the training process in volleyball. In order to follow their normal distribution of motor development, it is necessary that those training processes include exercises for basic motor development. Training processes are often oriented to the early sports specialization and achievement of greatest results at the earliest age. **PURPOSE**: To evaluate the effects of two types of volleyball training processes on motor development. **METHODS**: Seventy-six girls, average age of 8.6 ± 0.8 years, from two different volleyball schools participated in this study. Both volleyball groups had training 2 times per week for 19 weeks. Control group conducted program containing only volleyball elements exercises. The experimental group conducted a program containing volleyball elements exercises and 30-minute polygon with basic motor development exercises. **RESULTS**: T-test showed significant differences between initial and final results in the experimental group (jumping on one leg, standing on one leg – eyes closed, dribbling a tennis ball, running 10x4). In the control group, a significant difference was shown only in one test (dribbling a tennis ball). **CONCLUSION**: The results of this study showed that programs based on the multilaterally oriented physical activity must be included in sport specific training program for children’s normal motor development. They show certain advantages over the more specific program of volleyball school.

### P96 Comparative analysis of anthropometric parameters as obesity indicators for seven-year-old children of different resident status

#### Milena Mitrovic, Katarina Dragutinovic

##### Faculty of Sport and Physical Education, University of Montenegro, Montenegro

###### **Correspondence:** Milena Mitrovic (mitrovic.m@ac.me)

One of the major medical problems of today in the world is the obesity of children. Obesity is a chronic disease that is exacerbated by excessive fat accumulation in the body and increased body weight. **PURPOSE**: To determine the nutrition of seven-year-old children in the urban (Podgorica) and rural area (Zupa) of Montenegro and to determine whether there is a statistically significant difference in nutrition among them. **METHOD**: The sample of respondents consisted of 66 children seven-years-old from Podgorica and Zupa (Niksic) divided into 4 sub-samples according to the criteria of gender and place of residence. Each respondent was calculated BMI and WHR, which were shown numerically and in percentages, and the differences were determined using the HI square test. **RESULTS**: The results of the study showed that there are statistically significant differences in overweight and obesity among children of both genders from Podgorica and Zupa, where the children from Podgorica had a significantly higher percentage of children from Zupa. **CONCLUSION**: Children from rural areas are less obese than children in urban areas. The reason for this should be found in the fact that children in the rural area are more physically active and healthier foods, which are the main reasons for preventing obesity.

### P97 Morphological characteristics and body composition of soccer players in Montenegro and Bosnia and Herzegovina

#### Jovan Gardasevic^1^, Dusko Bjelica^1^, Zoran Milosevic^2^, Marin Corluka^3^

##### ^1^Faculty for Sport and Physical Education, University of Montenegro, Montenegro; ^2^Faculty of Sport and Physical Education, University of Novi Sad, Serbia; ^3^Faculty of Mathematics and Science Education, University of Mostar, Bosnia and Herzegovina

###### **Correspondence:** Jovan Gardasevic (jovan@ucg.ac.me)

**PURPOSE**: The aim was to determine the differences among the top soccer players of the Montenegro and Bosnia and Herzegovina, in the anthropometric measures and body composition. **METHODS**: The first sub-sample of the examinees consisted of 23 players of FC Sutjeska-Niksic (age 21.69±4.30 years), the champions of Montenegro Cup in 2016/17, while the other sub-sample consisted of 28 players of CSC Zrinjski Mostar (24.36±4.14 age), the champions of the Bosnia and Herzegovina in 2016/17. Soccer players were tested immediately after the end of the competition season 2016/17. Anthropometric measures and the body composition were evaluated by a battery of 10 variables: body height, body weight, waist size, triceps skin set, biceps skin set, back skin set, abdominal skin set, body mass index, fat percentage, and muscle mass. The significance of the differences in the anthropometric measures and variables for assessing body composition was determined by a t-test for independent samples. **RESULTS**: It was found that the soccer players of the two mentioned clubs don’t have statistically significant differences by the variables. **CONCLUSION**: It can be concluded that the soccer players of these two teams have very similar anthropometric characteristics.

### P98 Comparison of hand and electronic timing during 20 and 30 m sprints in primary school

#### Spela Bogataj^1^, Miloš Ignjatovic^2^

##### ^1^Faculty of Sport, University of Ljubljana, Slovenia; ^2^Elementary school “Kralj Aleksandar I”, Gornji Milanovac, Serbia

###### **Correspondence:** Spela Bogataj (sspelabogataj@gmail.com)

Sprinting speed is an important physical attribute in sport and physical education and therefore is often trained and tested. Although the ideal option would be to use photoelectric cells, the most commonly used measurement tool is a stopwatch because it is much cheaper and feasible in certain settings, such as schools. **PURPOSE**: The aim of this study was to compare hand timing and electronic timing during the 20-meter and 30-meter sprints. **METHODS**: Subjects included 40 primary school children (10.2 ± 0.40 years). The test sprints were simultaneously evaluated with hand and electronic timing. **RESULTS**: A paired samples t-test revealed that hand timing resulted in faster sprint times than electronic timing for both the 20 m (p ≤ 0.05) and 30 m sprints (p ≤ 0.05). Compared to the hand timing, manual timing yielded times were an average of 0.21 seconds faster in the 20 m sprint. Mean hand timing sprint times during the 30 m sprint were an average of 0.28 seconds faster than the electronic timing condition. **CONCLUSION**: Electronic timing is the gold standard for measuring speed, and using the hand timing produces consistently but significantly faster times. Results of this study provide practitioners with information that allows accurate interpretation of differences between hand and electronic timing methods.

### P99 Body mass index and blood pressure-to-height ratio in predicting the incidence of hypertension in Children

#### Valerija Puskas, Tatjana Pavlica, Rada Rakic

##### Department for Biology and Ecology, Faculty of Sciences, University of Novi Sad, Serbia

###### **Correspondence:** Tatjana Pavlica (tatjana.pavlica@dbe.uns.ac.rs)

Recently a new method using blood pressure-to-height ratio for diagnosing hypertension in children has been introduced. **PURPOSE**: To compare Body Mass Index (BMI) and blood pressure-to-height ratio (BPHR) in predicting the incidence of hypertension. **METHODS**: The surveys included 1133 boys and 1154 girls aged 7 - 15. The following equations for BPHR were used: systolic BPHR (SBPHR)=SBP (mm Hg)/height (cm) and diastolic BPHR (DBPHR)=DBP (mm Hg)/height (cm). BMI was calculated as weight (kg) divided by height squared (m^2^). Receiver-operating characteristic curve analyses were performed to assess the accuracy of SBPHR, DBPHR, and BMI as diagnostic tests for elevated blood pressure. The sensitivity and specificity of BPHR and BMI as indicators of hypertension were determined with cut-off values. **RESULTS**: The BMI-for-age Z-score has a modest ability to identify children with pre-hypertension and AUC values ranging from 0.625 to 0.723 with quite low sensitivity rates from 62% to 72.5% and specificities from 58.2% to 67, 3%. BPHR has a great predictive ability to identify pre-hypertension and hypertension with AUC values of 0.836 to 0.949 for SBP and from 0.777 to 0.904 for DBP. The sensitivity ranged from 78.5% to 95.7%, and the specificity from 73.9% to 87.6%. **CONCLUSION**: BPHR is a simple and accurate index for detecting hypertension in children aged 7 to 15 years and can be used for early screening.

### P100 Predictors of sports and recreation activity in youth in Serbia

#### Tanja Tomasevic^1,2^, Vesna Mijatovic Jovanovic^1,2^, Snezana Ukropina^1,2^, Sonja Susnjevic^1,2^, Dragana Milijasevic^1,2^, Natasa Dragnic^1,2^

##### ^1^Faculty of Medicine, University of Novi Sad, Novi Sad, Serbia; ^2^Institute of Public Health of Vojvodina, Novi Sad, Serbia

###### **Correspondence:** Tanja Tomasevic (tanja.tomasevic@izjzv.org.rs)

According to the World Health Organization recommended a level of physical activity for youth aged 18-26 years is at least 150 minutes of moderate-intensity aerobic physical activity throughout the week. **PURPOSE**: To examine the role of sociodemographic factors and risk behaviors (smoking and alcohol consumption) as predictors of sports and recreation in youth. **METHODS**: Data from the National Health Survey in Serbia 2013 was analyzed on the sample of 1540 youth aged 18-26. **RESULTS**: In Serbia, only 18.0% of youth had a recommended level of physical activity, 13.7% exercised 60-149 minutes, and 68.3% exercised less than 60 minutes per week or did not exercise. Young men (OR=4.3; 95% CI=2.6-7.2) compared to girls and youth aged 18-20 (OR=3.8; 95% CI=2.1-6.7) compared to youth aged 24-26, were more likely to have physical activity 150 and more minutes per week. Youth who belong to the category of rich (OR=3.2; 95% CI=1.7-5.9) compared to the poor category as well as non-smokers (OR=2.8; 95% CI=1.7-4.7) compared to smokers, were more likely to have a preferred level of physical activity. **CONCLUSION**: More than 2/3 of youth did not achieve the recommended level of physical activity for ensuring good health. Results point to the importance of the development of specific programs and strategies for increasing physical activity.

### P101 Correlation between attack contact time and the height and strength of the jump in volleyball

#### Cosmin Strava^1,2^, S Gradinaru^1^

##### ^1^Faculty of Sports and Physical Education, West University of Timisoara, Romania; ^2^Babeş-Bolyai University of Cluj, Romania

###### **Correspondence:** Cosmin Strava (strava_cosmin@yahoo.com)

Volleyball is a complex sports game that requires a high volume of motor, technical and tactical skills. In order to gain success in the competition, the coaches focus more and more on the offensive force of the team concretized by the spike. **PURPOSE**: To determine how the strength of the lower limbs (L.L) and the height of the jump are influenced by the contact time on the ground during the last two steps of the impulse. **METHODS**: 16 cadets (14-16 years of age, SMM 28.39±3.3, PBF 20.58%±2,478, BMI 21.01±3,982) from a volleyball club in Timisoara were tested for this study. We used InBody720 to evaluate the body composition and Optojump Next System using the "Drop Jump" test to assess the attack spike. **RESULTS**: After the statistical analysis, a reasonable (r=-0.4520) but statistically insignificant (p=0.07) correlation was obtained, between the contact time (TC) on the ground in the last two steps and the height of the jump, as well a high correlation (r=-0.7996) and a statistically significant (p=0.0002) between TC and the power emitted by L.L. **CONCLUSIONS**: It appears that when T.C. is shorter, the player gets a higher power in L.L which results in a higher jump.

### P102 Anginose symptoms and/or positive ECG stress test in recreational and professional athletes

#### Drazen Lovric^1^, Z Babic^2,3^, L Pavic^4^, M Jukic^5^

##### ^1^Special Hospital for Orthopaedic Surgery, Zagreb, Croatia; ^2^University Clinical Hospital Center “Sestre Milosrdnice”, Zagreb, Croatia; ^3^Faculty of Kinesiology, University of Zagreb, Croatia; ^4^Self-employed; ^5^Sunce Clinics, Zagreb, Croatia

###### **Correspondence:** Drazen Lovric (drazenlovric3@gmail.com)

Myocardial bridging (MB) is a congenital anomaly that is present when a segment of a major coronary artery runs intramurally. It usually has benign prognosis but also shows highly variable clinical manifestations, especially in young athlete population, from clinically silent, asymptomatic athletes to those who are presented with positive ECG stress test or chest pain symptoms that can be associated with ischemic clinical syndromes. **PURPOSE**: To evaluate whether MB alone is one of the most common causes of anginose disorders in that age and population group. **METHODS**: This study is a part of a larger retrograde study in which we have analyzed the rate of recreational and professional athletes with MB under the age of 36 with positive ECG stress test or anginose symptoms. All patients have undergone detailed clinical examination, ECG stress testing, and CCTA. **RESULTS**: Young athletes with positive ECG stress test or anginose symptoms have the highest positive prediction for having myocardial bridging. **CONCLUSION**: Despite the most common benign nature of MB, and that the described symptoms in young athletes are most often associated with the presence of MB, we are of the opinion that such symptoms always require full cardiological evaluation.

### P103 Association between physical activity and quality of life in college students

#### Lucijan Supljika Gabelica^1^, Bruno Lazinica^2^

##### ^1^Department of Croatian Studies, University of Zagreb, Croatia; ^2^Faculty of Education, Josip Juraj Strossmayer University of Osijek, Croatia

###### **Correspondence:** Lucijan Supljika Gabelica (lucijan.s.g@gmail.com)

**PURPOSE:** To determine if participation in physical activities (PA) have an impact on students’ quality of life. **METHODS**: 577 students (273 female, 304 male) of four different faculties of the University of Zagreb responded to a survey questionnaire. The first part of the survey was used to determine the PA level (IPAQ) and the second part for evaluation of life quality (WHOQOL-BREF). **RESULTS:** 366 participants perform vigorous PA at least once a week, out of which 49,5% perform 1-2 days, 35,8% 3-4 days, 7,7% 5 days and 7,1% of them perform 6-7 days. 416 participants perform moderate PA, out of which 65,4% perform 1-3 days, 22,3% 4-5 days and 12,2% of them perform 6-7 days. 58% of all participants walk every day for at least 10 minutes. 59,4% of participants exercise weekly and 16,5% of them exercise on a daily basis. Of those who exercise weekly, 34,7% is in the range of 1-2 hours and 13,3% exercise more than 5 hours weekly. 8% perceive their life as very poor or poor, 27,9% neither poor nor good, 47,9% as good and 16,3% as very good. Analysis of data indicates a high positive correlation (r=0,97) between the time of PAs and quality of life. **CONCLUSION:** Students who are more physically active perceive their quality of life as higher compared to students who are less active.

### P104 My Jump 2 APP for measuring different jump variables in college athletes

#### Jagoda Zecevic^1^, Amelia A. Ornato^2^, Nebojsa Trajkovic^1^, Tatjana Jezdimirovic^1^, Sasa Semeredi^1^, Valdemar Stajer^1^, Sergej M. Ostojic^1^

##### ^1^Faculty of Sport and Physical Education, University of Novi Sad, Serbia; ^2^Health Science Department, Stetson University, Deland, Florida, USA

###### **Correspondence:** Valdemar Stajer (stajervaldemar@yahoo.com)

The use of smartphone applications estimating vertical jump outcomes has recently emerged as a simple, cheap and practical tool to evaluate the performance capabilities of athletes and physical fitness of non-athlete individuals. **PURPOSE**: To evaluate the reliability and validity of My Jump 2 App for the measurement of vertical jump performance. **METHODS**: Forty-five college athletes (22 women and 23 men; BMI 22.7 ± 2.5 / 23.2 ± 2.3 kg/m2) performed the following jumps: squat jump (SJ), countermovement jump (CMJ), vertical jump (VJ) and single-leg CMJ (CMJ L/R), evaluated via contact mat and My Jump 2 App. **RESULTS**: The Pearson correlation between the contact mat and My Jump 2 App revealed a high correlation ranging from r = 0.92-0.99. The parameters of reliability for different methods of jump evaluation ranged from 0.91 to 0.99 for Cronbach's alpha coefficients, and from 0.96 to 0.99 for the intraclass correlation coefficient. The coefficient of variation also declares good reliability (CV% < 5) for SJ, CMJ, and VJ but single-leg CMJs were beyond the reliability criterion (CV% > 5). **CONCLUSION**: My Jump 2 App appears to be a valid and reliable tool for evaluation of vertical jump performance in college athletes.

### P105 The influence of dance training on motor coordination of younger school age girls

#### Aleksandra Spasic, Boris Popovic, Dejan Madic, Nebojsa Trajkovic, Danilo Radanovic

##### Faculty of Sport and Physical Education, University of Novi Sad, Serbia

###### **Correspondence:** Aleksandra Spasic (play.dance.studio@gmail.com)

In everyday life, an adequate motor coordination level in children is important for their general development, but also there is a consensus that good motor coordination is important for the health and well-being of children. **PURPOSE**: The aim of this research was to determine the differences in motor coordination (MC) among the respondents who are engaged in modern dance and their peers who do not deal with any type of physical activity. **METHODS**: A total of 173 girls aged 7 to 11 years, divided into 2 groups (experimental group/control group), were measured for MC using Körperkoordinationstest für Kinder (KTK). All girls performed four subtests: walking backward (WB), moving sideways (MS), hopping for height (HH) and jumping sideways (JS). Raw scores for each subtest were transformed into gender and age-specific motor quotients (MQ) values. **RESULTS**: The results of multivariate analysis ANOVA display that there are differences between divided groups in all of the examined KTK subtest, on the level of statistical significance of p=0.05. **CONCLUSION**: Based on the results of this research, the authors came to the conclusion that the motor coordination of girls who are engaged in modern dance is much more and better developed and therefore, that the dance is positively affecting the transformation of the motor coordination of young girls.

### P106 Physical activity and nutrition habits of primary school students

#### Angelina Matic, Lidija Markovic, Visnja Djordjic

##### Faculty of Sport and Physical Education, University of Novi Sad, Serbia

###### **Correspondence:** Lidija Markovic (markoviclidija169@gmail.com)

Decreased physical activity and poor nutrition habits can have a negative influence on youth’s health, both short- and long-term. The lack of comparable data on physical activity and diet of Serbian youth is evident. **PURPOSE**: To examine the physical activity and nutrition habits of primary school students from Bajina Basta. **METHODS**: The sample consisted of 331 participants (178 males and 153 females; 11-15 years old) from primary schools in Bajina Basta (Serbia). Physical activity and nutrition habits were assessed by respective subscales of a short version of Health-Promoting Lifestyle II. Data were analyzed by descriptive statistics and multivariate analysis of variance. **RESULTS**: Mean scores for physical activity and nutrition were 2.54 and 2.79, respectively. Boys and girls do not differ significantly in physical activity (p=.529) and nutrition indicators (p=.089). In addition, no age-related differences were identified in physical activity behavior (p=.981) and nutrition (p=.887). The most favorable physical activity behavior referred to participation at a planned exercise program (54.7%). As for nutrition habits, the highest percentage reported regularly eating breakfast (91.2%). However, some health-promoting behaviors were underrepresented. **CONCLUSIONS**: Primary school students’ physical activity and nutrition lifestyle habits are not gender or age-related. A tailored local health promotion intervention can be designed according to results obtained.

### P107 What Do future physical and health education teachers think of grades

#### Lidija Markovic, Visnja Djordjic

##### Faculty of Sport and Physical Education, University of Novi Sad, Serbia

###### **Correspondence:** Lidija Markovic (markoviclidija169@gmail.com)

Grading is the process of assigning marks based on a formal assessment of changes in student behavior. It is usually thought that grades can increase students' motivation by informing them about their achievement and progress. However, grades were criticized by some because of their controlling aspect. **PURPOSE:** To examine future PE and health teachers’ attitudes on grades in physical education. **METHODS:** The sample comprised of 363 3th-year- students of Faculty of Sport and Physical Education in Novi Sad (189 males, 87 females). Data on GPA was collected and the attitudes on grades were assessed by Questionnaire on Grades in Physical Education. Data were analyzed by descriptive statistics, independent Samples T-test, and One-way ANOVA. **RESULTS:** The future PE and health teachers reported a slightly positive attitude toward grades in physical education (M = 3.40, SD =.59). No significant gender (p=.334) or GPA-related (p=.207) differences were identified in students' attitudes toward grades in PE. In comparison to a similar study conducted twenty years ago, significant differences were detected in favor of previous students in gender (p=.0394) and GPA (p=.0154). **CONCLUSIONS:** By showing a slightly positive attitude toward grades in physical education, future teachers seem to rely on traditional functions of grades in the educational process.

### P108 Vertical jump performance of female volleyball players of different competition level

#### Dusko Cvijovic, Roberto Roklicer, Suncica Pocek

##### Faculty of Sport and Physical Education, University of Novi Sad, Serbia

###### **Correspondence:** Suncica Pocek (suncicapocekfsfv@gmail.com)

Vertical jumps are performed frequently by volleyball players during practices and games. The question of whether vertical jump performance differs between more proficient players and less proficient players is of importance. **PURPOSE**: To examine vertical jump performance of three different competition level teams. **METHODS**: Thirty-four volleyball players were assessed for height, mass, spike reach, block reach and vertical jump performance – countermovement jump with arms swing (CMJa), spike (SJ) and block jump (BJ). MANOVA with LSD post hoc test was applied in order to examine whether there were statistically significant differences between Team A, B, and C (17.93±1.33, 8.58±2.84; 16.59±0.93, 5.29±2.06; 14.41±0.61, 3.87±1.30 age and training years respectively), in vertical jumping performance. **RESULTS:** The MANOVA revealed a significant difference for competition level (F=2.05; p<0.05; partη^2^=0.27). The subsequent analysis have revealed that both absolute CMJa 268.58±5.45, 264.43±10.44, 258.27±9.23; SJ 272.92±6.40, 268.71±13.41, 261.93±8.82 and BJ 258.92±4.64, 258.00±9.88, 250.80±8.22 and relative CMJa 40.25±2.38, 37.71±5.82, 34.40±5.30, SJ 44.58±3.96, 42.00±8.93, 38.07±6.13 and BJ 33.75±2.90, 33.86±6.23, 29.80±4.81 values of vertical jump performance were better in Team A than B and C volleyball players. Based on LSD post hoc test, statistically significant differences were observed only between Team A and Team C. **CONCLUSION:** Observed differences in jumping performance of volleyball players according to competition level could’ve been a consequence of different age, training years and skill level.

### P109 Evaluation of human resources in the sports system of Bosnia and Herzegovina

#### Dejana Planincic, Said Fazlagic, Izet Radjo

##### Olympic Committee of Bosnia and Herzegovina, Bosnia and Herzegovina

###### **Correspondence:** Dejana Planincic (okbih@okbih.ba)

Given the fact that the human resources are an essential element in every organization one can conclude that by improving the competences of human resources within an organization you are strengthening the capacity of the organization itself, therefore an evaluation of the human resource capacities of the National Sport Federations of Bosnia and Herzegovina has been conducted. The results will be used for the current state analysis and will lead to a possible recommendation for the improvement of this segment. **PURPOSE**: To evaluate human resources as a crucial factor in the effectiveness and efficiency of every sports system. **METHODS:** The National Sport Federations of Bosnia and Herzegovina participated in the evaluation. The UMAP tool (understanding, managing, assessing and planning) was used for the evaluation of the capacities of the Bosnian Sport Federations. **RESULTS**: The results indicate that human resources are more effective and efficient if they possess specific education and adequate experience. **CONCLUSION**: Based on conducted analyses and the obtained results there is a basic need for informal education model to be developed.

